# PAH/PAH(CF_3_)_*n*_ Donor/Acceptor Charge‐Transfer Complexes in Solution and in Solid‐State Co‐Crystals

**DOI:** 10.1002/chem.201902712

**Published:** 2019-10-07

**Authors:** Karlee P. Castro, Eric V. Bukovsky, Igor V. Kuvychko, Nicholas J. DeWeerd, Yu‐Sheng Chen, Shihu H. M. Deng, Xue‐Bin Wang, Alexey A. Popov, Steven H. Strauss, Olga V. Boltalina

**Affiliations:** ^1^ Department of Chemistry Colorado State University Fort Collins CO 80523 USA; ^2^ ChemMatCARS University of Chicago, Advanced Photon Source Argonne IL 60439 USA; ^3^ Physical Sciences Division Pacific Northwest National Laboratory, MS K8 88 P.O. Box 999 Richland Washington 99352 USA; ^4^ Leibniz Institute for Solid State and Materials Research Dresden 01069 Germany

**Keywords:** charge transfer, density functional calculations, polycyclic aromatic hydrocarbons, trifluoromethyl substituents, X-ray diffraction

## Abstract

A solution, solid‐state, and computational study is reported of polycyclic aromatic hydrocarbon PAH/PAH(CF_3_)_*n*_ donor/acceptor (D/A) charge‐transfer complexes that involve six PAH(CF_3_)_*n*_ acceptors with known gas‐phase electron affinities that range from 2.11(2) to 2.805(15) eV and four PAH donors, including seven CT co‐crystal X‐ray structures that exhibit hexagonal arrays of mixed π‐stacks with 1/1, 1/2, or 2/1 D/A stoichiometries (PAH=anthracene, azulene, coronene, perylene, pyrene, triphenylene; *n=*5, 6). These are the first D/A CT complexes with PAH(CF_3_)_*n*_ acceptors to be studied in detail. The nine D/A combinations were chosen to allow several structural and electronic comparisons to be made, providing new insights about controlling D/A interactions and the structures of CT co‐crystals. The comparisons include, among others, CT complexes of the same PAH(CF_3_)_*n*_ acceptor with four PAH donors and CT complexes of the same donor with four PAH(CF_3_)_*n*_ acceptors. All nine CT complexes exhibit charge‐transfer bands in solution with *λ*_max_ between 467 and 600 nm. A plot of *E*(*λ*_max_) versus [*IE*(donor)−*EA*(acceptor)] for the nine CT complexes studied is linear with a slope of 0.72±0.03 eV eV^−1^. This plot is the first of its kind for CT complexes with structurally related donors and acceptors for which precise experimental gas‐phase *IE*s and *EA*s are known. It demonstrates that conclusions based on the common assumption that the slope of a CT *E*(*λ*_max_) versus [*IE*−*EA*] plot is unity may be incorrect in at least some cases and should be reconsidered.

## Graphical abstract

**Accept the charge**: Comprehensive solution/solid‐state/computational study of polycyclic aromatic hydrocarbon PAH/PAH(CF_3_)_*n*_ donor/acceptor charge‐transfer complexes involving PAH(CF_3_)_*n*_ acceptors with electron affinities from 2.11 to 2.85 eV and four PAH donors with ionization energies from 6.96 to 7.44 eV, including seven X‐ray crystal structures exhibiting hexagonal arrays of mixed π‐stacks with 1/1, 1/2, or 2/1 D/A stoichiometries.

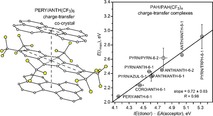

## Introduction

Organic co‐crystalline materials containing electronically‐ and structurally‐tuneable aromatic donors and acceptors exhibit a wide range of physicochemical properties that have found, or are expected to find, use in molecular electronic applications.^1^, ^2^, ^3^, ^4^, ^5^, ^6^, ^7^, ^8^, ^9^, ^10^ Of particular interest are the ways that strong electron‐withdrawing groups can affect not only the electronic coupling of donors and acceptors but the degree of π–π overlap and one‐dimensional stacking.^11^, ^12^, ^13^ In particular, F atoms^14^, ^15^, ^16^, ^17^, ^18^, ^19^ and perfluoroalkyl (R_F_) groups^20^, ^21^, ^22^, ^23^ can have desirable electronic and structural effects. For example, R_F_ substitution can raise the electron affinities (*EA*s) of polycyclic aromatic hydrocarbons (PAHs) by 0.3–0.5 eV per R_F_ group^22^, ^24^, ^25^, ^26^ and strengthen parallel π–π interactions while suppressing T‐shaped C−H⋅⋅⋅π interactions.^27^, ^28^ Fluorination, even partial fluorination, can also increase PAH electron affinities,^29^, ^30^ improve air stability,^24^, ^31^, ^32^ affect PAH charge mobility^33^ and photophysics,^34^ convert p‐type into n‐type semiconductors^24^, ^31^, ^32^, ^35^ (e.g., perfluoropentacene^31^, ^35^), and convert PAH herringbone structures (e.g., anthracene (ANTH)^36^) into π‐stacked structures (e.g., 1,2,3,4‐ANTH(F)_4_ 37).^38^, ^39^ However, CF_3_ is a much stronger electron‐withdrawing group (EWG) than F.^22^, ^40^, ^41^ For example, the DFT‐predicted *EA*s of perfluoroanthracene (ANTH(F)_10_) and ANTH(CF_3_)_10_ are 1.84^29^, ^30^ and 4.01 eV,^24^ respectively. With only six CF_3_ groups, the experimental *EA* of 2,3,6,7,9,10‐ANTH(CF_3_)_6_ (ANTH‐6‐1), at 2.81(2) eV,^26^ is more than 1 eV higher than ANTH(F)_10_ and is the same as the 2.78(6) eV *EA* of the common charge‐transfer electron acceptor chloranil (2,3,4,5‐tetrachlorobenzoquinone; Cl_4_BQ).^42^ Another difference between CF_3_ and F substituents on PAHs is exemplified by the order of photostability of 9,10‐ANTH(X)_2_ derivatives in aerated cyclohexane: CF_3_>H>F.^43^ Although PAH/PAH(F)_*n*_ co‐crystals have been studied for many years (e.g., co‐crystals of perfluoronaphthalene with pyrene^44^ and fluorene^45^), to our knowledge there are no reports of PAH/PAH(CF_3_)_*n*_ co‐crystals other than our brief communication of the structures of co‐crystals containing pyrene and either 1,2,3,5,7‐azulene(CF_3_)_5_ (AZUL‐5‐1)^46^ or ANTH‐6‐1^47^ (both of which will be discussed in greater detail in this paper) and the co‐crystal structure of two complementary π‐bowls, corannulene/1,3,5,7,9‐corannulene(CF_3_)_5_.^48^

We have studied the synthesis and physicochemical properties of 53 PAH(CF_3_)_*n*_ derivatives prepared by substituting H atoms in twelve unsubstituted PAHs using CF_3_ radicals generated from CF_3_I at high temperature (*n=*1–8).^22^, ^23^, ^25^, ^26^, ^46^, ^49^, ^50^, ^51^ In addition to their spectroscopic and electrochemical characterization, nearly two‐thirds of them have also been studied by single‐crystal X‐ray diffraction and have had their *EA*s determined by low‐temperature gas‐phase photoelectron spectroscopy. These strong electron acceptors, with *EA*s that range from 1.8 to 3.2 eV, are listed in Supporting Information Table S1. This table also includes information about the 16 other PAH(CF_3_)_*n*_ derivatives reported by others that were prepared by more conventional, multi‐step synthetic methods.^28^, ^52^, ^53^, ^54^, ^55^, ^56^, ^57^, ^58^, ^59^, ^60^, ^61^, ^62^, ^63^

**Figure 1:**
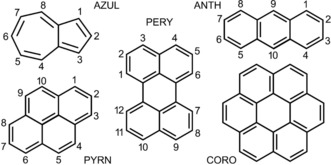
Drawings of the five polycyclic aromatic hydrocarbons (PAHs) used in this work: anthracene (ANTH); azulene (AZUL); coronene (CORO); perylene (PERY); pyrene (PYRN).

**Figure 2:**
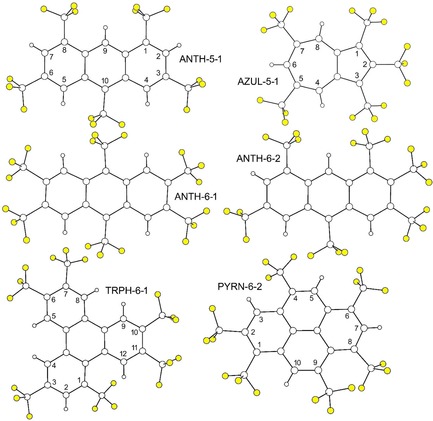
Drawings of the six PAH(CF_3_)_*n*_ electron acceptors used in this work: 1,3,6,8,10‐anthracene(CF_3_)_5_ (ANTH‐5‐1); 2,3,6,7,9,10‐anthracene(CF_3_)_6_ (ANTH‐6‐1); 1,2,3,6,8,10‐anthracene(CF_3_)_6_ (ANTH‐6‐2); 1,2,3,5,7‐azulene(CF_3_)_5_ (AZUL‐5‐1); 1,2,4,6,8,9‐pyrene(CF_3_)_6_ (PYRN‐6‐2); and 1,3,6,7,10,11‐triphenylene(CF_3_)_6_ (TRPH‐6‐1). Thermal ellipsoid plots of charge‐transfer co‐crystal X‐ray structures are shown in the Supporting Information.

We herein report a solution, solid‐state, and computational study of nine (PAH)_*x*_/(PAH(CF_3_)_*n*_)_*y*_ donor/acceptor (D/A) charge‐transfer (CT) complexes that involve the four PAH donors and six PAH(CF_3_)_*n*_ acceptors shown in Figure 1 and Figure 2, respectively. Their abbreviations, ionization energies (*IE*s), *EA*s, *E*_1/2_(+/0) or *E*_1/2_(0/−) electrochemical potentials, and longest wavelength CT *λ*_max_ values are listed in Table 1.^64^, ^65^, ^66^ These are the first D/A CT complexes with PAH(CF_3_)_*n*_ acceptors to be studied in detail. The nine D/A combinations were chosen to allow several comparisons to be made in order to provide new insights about controlling PAH D/A interactions and the structures of CT co‐crystals. The comparisons include combinations of the same PAH(CF_3_)_*n*_ acceptor (ANTH‐6‐1) with four different PAH donors (ANTH, CORO, PERY, and PYRN), the same PAH donor (ANTH) with three structurally‐similar PAH(CF_3_)_*n*_ acceptors (ANTH‐5‐1, ANTH‐6‐1, and ANTH‐6‐2), and the same PAH donor (PYRN) with four structurally‐different PAH(CF_3_)_*n*_ acceptors (AZUL‐5‐1, ANTH‐6‐1, PYRN‐6‐2, and TRPH‐6‐1). Five of the nine CT co‐crystals exhibit hexagonal arrays of alternating D/A/D/A π‐stacks, one exhibits D/D/A/D/D/A π‐stacks, two exhibit D/A/A/D/A/A π‐stacks, and one co‐crystal structure has discrete π‐stacked A/D/A triplets that are not stacked parallel to their nearest neighbour triplets. The D/A interplanar separation in all nine structures, 3.55±0.06 Å, is highly unusual for mixed‐stack CT complexes of donors and acceptors that have planar aromatic cores and comparable *IE*s and *EA*s, respectively (these typically have D/A interplanar separations of 3.32±0.06 Å^44^, ^67^, ^68^, ^69^, ^70^, ^71^, ^72^, ^73^, ^74^, ^75^). Thus, our study has afforded the rare opportunity to determine, both experimentally and with DFT theory, the effect of significantly increasing D/A interplanar separation on the degree of D/A π–π overlap, D/A relative orientations, and D/A CT energies.

**Table 1:** Electron Affinity (*EA*), Ionization Energy (*IE*), Electrochemical, and Spectroscopic Data.^[a]^

compound, solution, mixture in	abbr.	gas‐phase	gas‐phase	*E*_1/2_ [V vs. Fe(Cp)_2_^+/0^]		gas‐phase Δ(*IE*/*EA*)	longest wavelength *λ*_max_ value, nm [eV]^[d]^	
solution, thin film or co‐crystal		*IE* [eV]^[b]^	*EA* [eV]^[b]^	+/0 couple	0/− couple^[e]^	[eV]^[c]^	1,2‐C_2_H_4_Cl_2_ solution	thin film
anthracene (C_14_H_10_)	ANTH	7.439(6)	0.53(2)	0.71;^[f]^ 0.90^[g,h]^	−2.52	–	<400 [>3.11]	<400 [>3.11]
azulene (C_10_H_8_)	AZUL	7.42(2)	0.790(8)	0.33^[f]^	−2.14	–	579 [2.14]	–
coronene (C_24_H_12_)	CORO	7.26(5)	0.47(9)	0.85^[f]^	−2.61^[d]^	–	451 [2.75]	–
perylene (C_20_H_12_)	PERY	6.960(1)	0.973(5)	0.47;^[f]^ 0.59^[g,i]^	−2.23	–	523 [2.37]	467 [2.67]
pyrene (C_14_H_10_)	PYRN	7.426(1)	0.41(1)	0.78;^[f]^ 0.87^[g,h]^	−2.65	–	<400 [>3.11]	<400 [>3.11]
1,3,6,8,10‐ANTH(CF_3_)_5_	ANTH‐5‐1	–	2.40(2)^[e]^	–	−1.24	–	<400 [>3.11]	<400 [>3.11]
2,3,6,7,9,10‐ANTH(CF_3_)_6_	ANTH‐6‐1	–	2.81(2)^[e]^	–	−0.92	–	<400 [>3.11]	<400 [>3.11]
1,2,3,6,8,10‐ANTH(CF_3_)_6_	ANTH‐6‐2	–	2.68(2)^[d]^	–	−0.98^[d]^	–	<400 [>3.11]	–
1,2,3,5,7‐AZUL(CF_3_)_5_	AZUL‐5‐1	–	2.850(15)^[e,j]^	–	−0.73	–	536 [2.31]	–
1,3,4,6,8,9‐PYRN(CF_3_)_6_	PYRN‐6‐1	–	2.71(2)^[e]^	–	−1.01	–	<400 [>3.11]	–
1,2,4,6,8,9‐PYRN(CF_3_)_6_	PYRN‐6‐2	–	2.6–2.7^[d]^	–	−1.05^[d]^	–	<400 [>3.11]	–
1,3,6,7,10,11‐TRPH(CF_3_)_2_	TRPH‐6‐1	–	2.11(2)^[e]^	–	−1.73	–	<400 [>3.11]	–
ANTH/ANTH‐5‐1	–	–	–	–	–	5.04(2)	466 [2.66]	456 [2.72]
ANTH/ANTH‐6‐1	–	–	–	–	–	4.63(2)	523 [2.37]	530 [2.34]
ANTH/ANTH‐6‐2	–	–	–	–	–	4.76(2)	506 [2.45]	–
CORO/ANTH‐6‐1	–	–	–	–	–	4.45(5)	554 [2.24]^[k]^	–
PERY/ANTH‐6‐1	–	–	–	–	–	4.15(2)	600 [2.07]^[l]^	655 [1.90]
PYRN/ANTH‐6‐1	–	–	–	–	–	4.63(2)	510 [2.43]^[m]^	494 [2.51]
PYRN/AZUL‐5‐1	–	–	–	–	–	4.58(2)	510‐540 [2.43–2.30]	–
PYRN/PRYN‐6‐2	–	–	–	–	–	4.73–4.83	450‐500 [2.76–2.48]	–
PYRN/TRPH‐6‐1	–	–	–	–	–	5.32(2)	400‐450 [3.10–2.76]	–

[a] Least significant digit uncertainties shown in parentheses. *E*_1/2_ uncertainties are ±0.01 V. [b] Adiabatic values from NIST Webbook (ref. 64) unless otherwise indicated. [c] Δ(*IE*/*EA*)=*IE*(PAH)−*EA*(PAH(CF_3_)_*n*_. [d] This work. [e] From ref. 26 unless otherwise indicated; *E*_1/2_ measured in DME/0.1 m TBA^+^ClO_4_^−^. [f] Ref. 65; in CH_3_CN/2 m NaClO_4_ at 25.0(1)°. [g] Ref. 66. [h] In liquid SO_2_ at −51 °C. [i] In CH_2_Cl_2_ at −40 °C. [j] Erroneously reported as 2.890(15) in ref. 26. [k] Corrected from 532 nm [2.33 eV] in toluene; see text. [l] Longest wavelength CT band *λ*_max_ value. [m] Most intense CT band *λ*_max_ value.

## Experimental Section

### Materials

See Table 1 for a list of abbreviations for the PAH and PAH(CF_3_)_*n*_ compounds studied in this work. The generic abbreviation PAH(CF_3_)_*n*_ denotes a compound with *n* H atoms replaced by *n* CF_3_ groups (e.g., the composition of ANTH(CF_3_)_6_ is C_14_H_4_(CF_3_)_6_, not C_14_H_10_(CF_3_)_6_). The PAH electron donors ANTH (TCI America, 94 %), CORO (TCI America, 95.0 %), PERY (Sigma–Aldrich, 99 %), and PYRN (Alfa Aesar, 98 %) were used as received. Spectroscopy grade toluene (Burdick & Jackson) and ethyl acetate (Sigma–Aldrich), ACS grade dichloromethane (CH_2_Cl_2_) and 1,2‐dichloroethane (1,2‐C_2_H_4_Cl_2_, Fisher Scientific), HPLC grade heptane, acetonitrile (CH_3_CN), and methanol (Fisher Scientific), anhydrous dimethoxyethane (DME, Sigma–Aldrich, inhibitor free), NMR grade hexafluorobenzene (C_6_F_6_, Sigma–Aldrich, 99.5+%), trifluoroiodomethane (CF_3_I, Synquest Laboratories, 99 %), chloroform‐*d* (CDCl_3_, Cambridge Isotopes), and ferrocene (Fe(Cp)_2_, Strem Chemical) were used as received. Electrochemical analysis grade tetrabutylammonium perchlorate (N(*n*Bu)_4_ClO_4_, Sigma–Aldrich, 99+%) was recrystallized from ethyl acetate. The PAH(CF_3_)_*n*_ electron acceptors ANTH‐5‐1, ANTH‐6‐1, AZUL‐5‐1, and TRPH‐6‐1 were prepared as previously described.^26^, ^46^

### Synthesis of new compounds

**ANTH‐6‐2**: A mixture of ANTH (178 mg; 1.00 mmol) and CF_3_I (2.35 g; 12.0 mmol) at −196 °C were sealed under vacuum in a thick‐walled glass ampoule (internal volume 250 mL) and heated at 360 °C for 24 h as previously described.^26^ [CAUTION: The pressure in the ampoule was ca. 2.5 atm at 360 °C; the burst pressure for a glass ampoule of this type is determined in part by the quality of seal, so care must be taken during the sealing step. Safety shields, face shields, and heavy‐duty personal protection clothing must be used at all times, and only experienced personnel should perform this operation.] The ampoule was cooled to 22 °C and opened, and the soluble contents were removed using CH_2_Cl_2_. Rotary evaporation of the CH_2_Cl_2_ rinses also removed the I_2_ by‐product and left a white residue. The compounds ANTH‐5‐1 and ANTH‐6‐1 were separated by HPLC as previously described, using a Shimadzu Prominence HPLC system.^26^ A fraction that eluted between 8.6 and 10.0 min on a Cosmosil Buckyprep preparative column (Nacalai USA; 20 mm i.d.×200 mm; 100 % CH_3_CN eluent; 20 mL min^−1^ flow rate) contained ANTH‐6‐2 and an unidentified ANTH(CF_3_)_*n*_ compound. Further purification was performed using a FluoroFlash column (Fluorous Technologies, 4.6 mm i.d.×150 mm; in 95:5 CH_3_CN:H_2_O eluent; 2 mL min^−1^ flow rate). The compound ANTH‐6‐2 was collected in a fraction between 15.2 and 16.8 min, which was dried to a white powder by rotary evaporation. Yield: ca. 20 mg (3.5 % based on ANTH). The ^1^H and ^19^F NMR spectra of ANTH‐6‐2 (Figure S1 in Supporting Information) demonstrated that the purity of ANTH‐6‐2 was 98+ mol %.

**PYRN‐6‐2**: A mixture of PYRN (20 mg, 0.10 mmol) and CF_3_I (5.9 g, 3.0 mmol) at −196 °C were sealed under vacuum in a thick‐walled glass ampoule (internal volume 50 mL) and heated at 360 °C for 24 h as previously described.^26^ The ampoule was cooled to 22 °C and opened, and the soluble contents were removed using CH_2_Cl_2_. Rotary evaporation of the CH_2_Cl_2_ rinses also removed the I_2_ by‐product and left a yellow residue. The compound PYRN‐6‐2 was separated by three stages of HPLC purification. The first stage involved a Cosmosil Buckyprep preparative column (see above; 100 % CH_3_CN eluent; 20 mL min^−1^ flow rate). The fraction that eluted between 28.2 and 34.6 min was reduced in volume and injected onto a FluoroFlash column (see above; 100 % CH_3_CN eluent; 2 mL min^−1^ flow rate). The fraction that eluted between 6.1 and 6.7 min was dried to a yellow residue by rotary evaporation, re‐dissolved in toluene, and injected onto a Cosmosil Buckyprep semi‐preparative column (Nacalai USA, 10 mm i.d.×200 mm; 60:40 toluene:heptane eluent; 5 mL min^−1^ flow rate). The compound PYRN‐6‐2 was collected in a fraction that eluted between 3.5 and 3.8 min, which was dried to a yellow powder by rotary evaporation. Yield: ca. 6 mg (10 % based on PYRN). The ^1^H and ^19^F NMR spectra (Figure S2) demonstrated that the purity of PYRN‐6‐2 was 98+ mol %.

### Electrochemical and spectroscopic measurements

Cyclic voltammograms were recorded with a three‐electrode cell in a purified dinitrogen‐filled glovebox at Colorado State University (CSU) as previously described.^26^ The solutions were ca. 1–2 mm analyte in deoxygenated DME containing 0.10 m N(*n*Bu)_4_ClO_4_. The ^1^H (400 MHz) and ^19^F (376 MHz) NMR spectra of CDCl_3_ solutions of ANTH‐6‐2 and PYRN‐6‐2 were recorded using a Varian Inova 400 spectrometer. Chemical shifts were referenced to internal residual CHCl_3_ (*δ*=7.27) or added C_6_F_6_ (δ=−164.9). UV/Vis spectra of CH_2_Cl_2_, 1,2‐C_2_H_4_Cl_2_, or toluene solutions of PAH and PAH(CF_3_)_*n*_ compounds and PAH/PAH(CF_3_)_*n*_ mixtures were recorded using a Cary 500 spectrophotometer. Equimolar solutions of various PAH/PAH(CF_3_)_*n*_ combinations were drop‐cast onto quartz microscope slides in order to record solid‐state thin‐film UV/Vis spectra. The adiabatic electron affinity (*EA*) of ANTH‐6‐2 was determined at Pacific Northwest National Laboratory as previously described,^22^ using a low temperature (10–12 K) apparatus that couples an electrospray ionization source and a temperature‐controlled ion trap to a magnetic‐bottle time‐of‐flight photoelectron spectrometer previously described in detail.^76^

### Single‐crystal X‐ray diffraction

The nine (PAH)_*x*_/(PAH(CF_3_)_*n*_)_*y*_ CT complex co‐crystals discussed in detail in this work were grown by slow evaporation of CH_2_Cl_2_ solutions (the structures of PYRN/AZUL‐5‐1^46^ and PYRN/(ANTH‐6‐1)_2_ 47 were reported previously, but were not described in detail). Diffraction data were collected at Colorado State University using a Bruker APEX‐II CCD diffractometer with a Mo_Kα_ X‐ray tube source and graphite monochromator (*λ*=0.71073 Å) or on beamline 15ID‐B at the Advanced Photon Source at Argonne National Laboratory, Argonne IL, using synchrotron radiation with a diamond (1 1 1) or graphite monochromator (*λ*=0.41328 Å). Unit cell parameters were obtained from least‐squares fits to the angular coordinates of all reflections, and intensities were integrated from a series of frames (ω and ϕ rotation) covering more than a hemisphere of reciprocal space. Absorption and other corrections were applied using SADABS.^77^ Each structure was solved using direct methods and refined (on *F* ^2^, using all data) by a full‐matrix, weighted least‐squares process. Standard Bruker control and integration software (APEX II) was employed,^78^ and Bruker SHELXTL software was used with Olex 2 for structure solution, refinement, and molecular graphics.^79^, ^80^

CCDC https://www.ccdc.cam.ac.uk/services/structures?id=doi:10.1002/chem.201902712 contain the supplementary crystallographic data for this paper. These data are provided free of charge by http://www.ccdc.cam.ac.uk/.

### DFT calculations

The atomic coordinates of all studied PAH, PAH(CF_3_)_*n*_, and PAH/PAH(CF_3_)_*n*_ charge‐transfer complexes were optimized at the B3LYP‐D3/def2‐TZVPP level^81^, ^82^, ^83^ using the NWChem code (version 6.5).^84^ Starting coordinates for D/A complexes were taken from the single‐crystal X‐ray structures. Solvation energies were computed with the COSMO solvation model,^85^, ^86^ as implemented in NWChem. CT excitation energies were computed using a constrained DFT (C‐DFT) approach,^87^ which enables calculation of the energy of a state in which the net Mulliken charges of the acceptor and donor are fixed to −1 and +1, respectively. The energies of such CT states were then compared to the ground‐state energies of the same complexes obtained by conventional DFT calculations with the same functional and basis set. As C‐DFT calculations experienced convergence problems when using the def2‐TZVPP basis set, the values reported in this manuscript were computed with the less extended 6–311G* basis. Comparison with def2‐TZVPP results (when the latter did converge) showed that the CT energies vary with the change of basis set by 0.12–0.29 eV (the 6–311G* values were systematically higher than their def2‐TZVPP counterparts).

## Results

### General comments

The crystal structures of the recently reported donor/acceptor (D/A) co‐crystals of PYRN with AZUL‐5‐1^46^ and PYRN with ANTH‐6‐1 (the 1/2 structure PYRN/(ANTH‐6‐1)_2_),^47^ and the seven X‐ray D/A co‐crystal structures we report here represent combinations of four PAH donors, ANTH, CORO, PERY, and PYRN, and six PAH(CF_3_)_*n*_ acceptors, ANTH‐5‐1, ANTH‐6‐1, ANTH‐6‐2, AZUL‐5‐1, PYRN‐6‐2, and TRPH‐6‐1. Table 1 lists their formulas (with IUPAC locants), ionization energies (*IE*s) and/or electron affinities (*EA*s), *E*_1/2_(+/0) and/or *E*_1/2_(0/−) values, and, for D/A mixtures, charge‐transfer (CT) *λ*_max_ and *E*(*λ*_max_) values. Table S1 in Supporting Information lists all PAH(CF_3_)_*n*_ compounds reported in the literature to date (i.e., compounds with at least two fused aromatic rings with 0, 1, or 2 N atoms, one or more CF_3_ groups, and no other substituents), including the new electron acceptors ANTH‐6‐2 and PYRN‐6‐2 reported here for the first time. The ^1^H and ^19^F NMR spectra of ANTH‐6‐2 and PYRN‐6‐2 are shown in Figures S1 and S2, respectively. The cyclic voltammograms of ANTH‐6‐2, PYRN‐6‐2, and CORO are shown in Figure S3. The recently published UV spectra of ANTH and ANTH‐6‐1 are shown in Figure S4.^47^ The similarity in *λ*_max_ values for ANTH and ANTH‐6‐1 shows that the HOMO–LUMO gaps for these two compounds must be similar, even though they have *EA*s that differ by nearly 2.3 eV (0.53(2) eV for ANTH^88^ and 2.81(2) eV for ANTH‐6‐1^26^).

### Spectroscopic characterization of PAH/PAH(CF_3_)_*n*_ CT complexes

**Figure 3:**
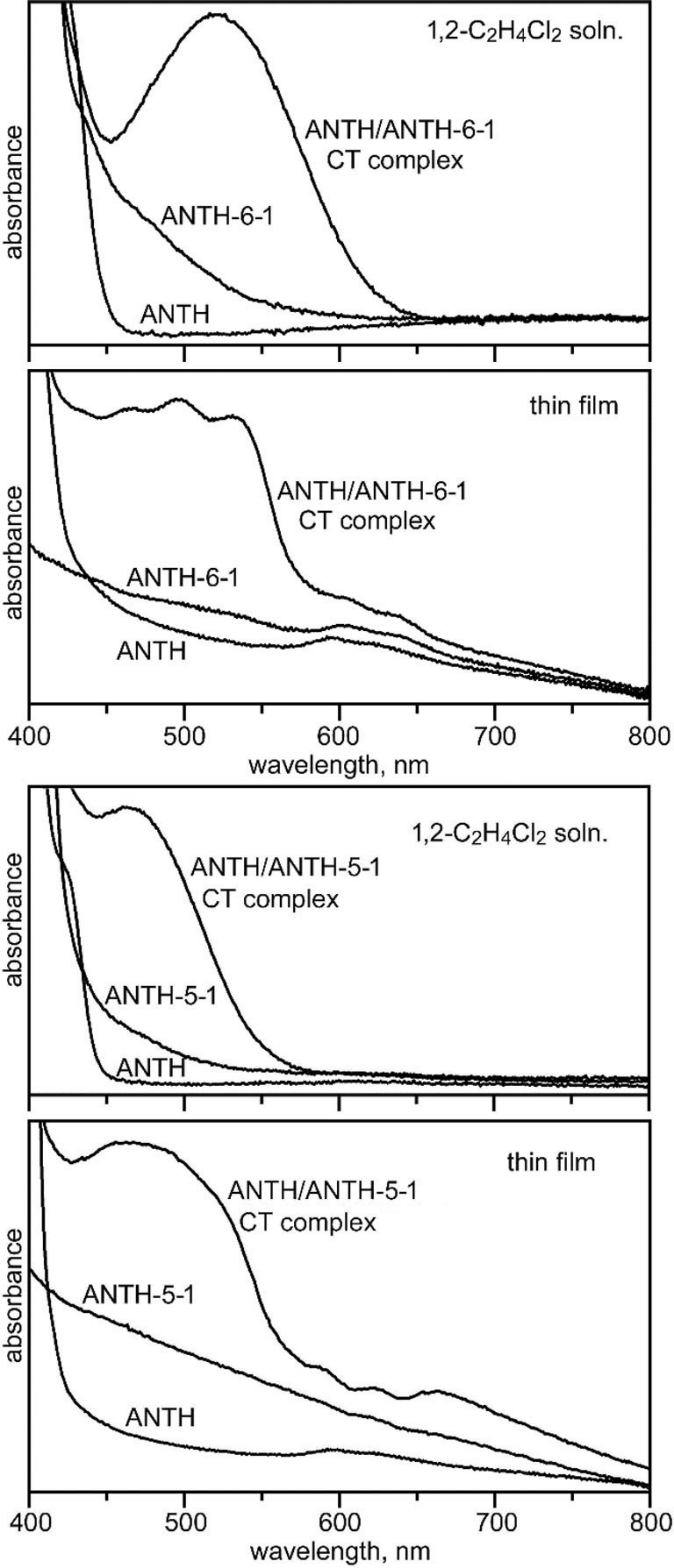
Solution and solid‐state (thin film) electronic spectra of ANTH, ANTH‐6‐1, and a mixture of them containing the 1/1 ANTH/ANTH‐6‐1 CT complex (top two panels) and ANTH, ANTH‐5‐1, and a mixture of them containing the 1/1 ANTH/ANTH‐5‐1 CT complex (bottom two panels). See Table 1 for *λ*_max_ values. The concentrations of samples for the solution spectra were ca. 5 mm.

**Figure 4:**
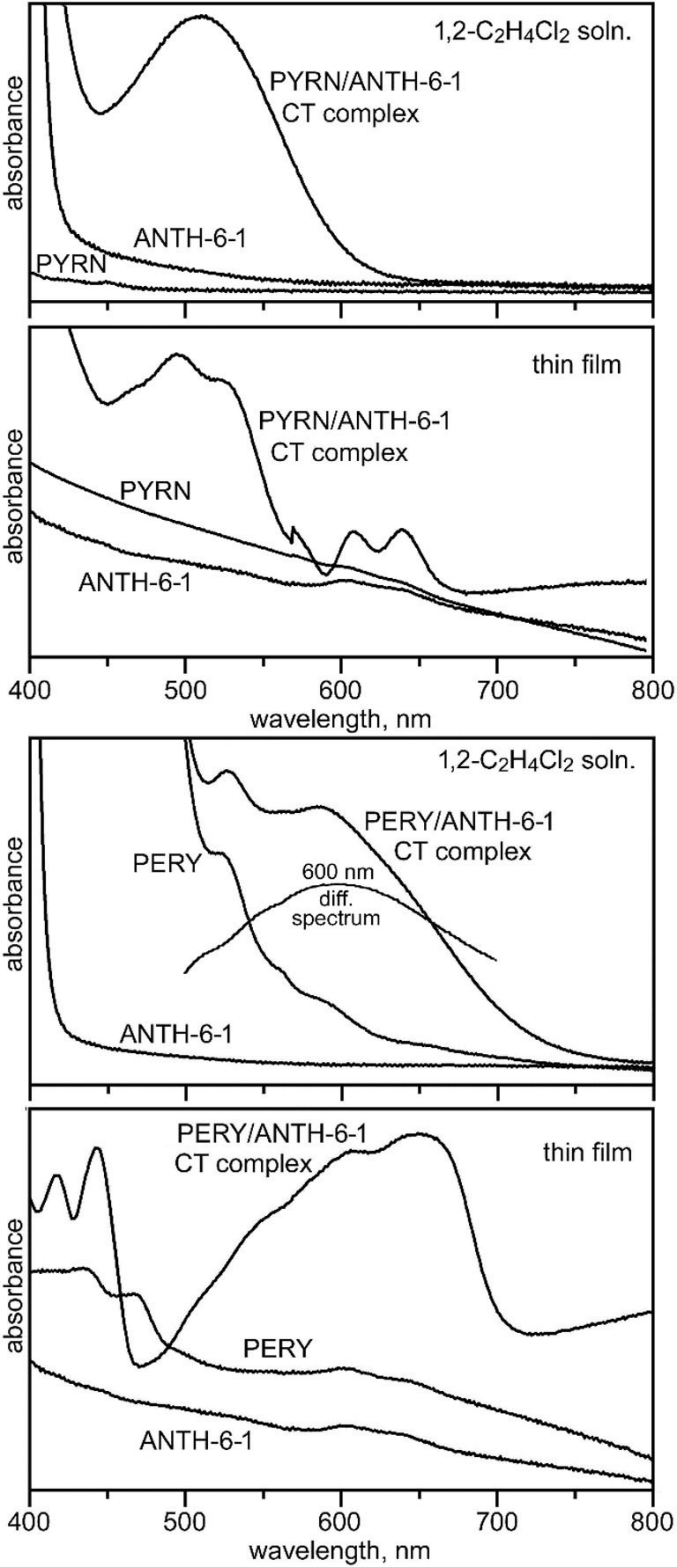
Solution and solid‐state (thin film) electronic spectra of PYRN, ANTH‐6‐1, and a mixture of them containing the 1/1 PYRN/ANTH‐6‐1 CT complex (top two panels) and PERY, ANTH‐6‐1, and a mixture of them containing the 1/1 PERY/ANTH‐6‐1 CT complex (bottom two panels). Part of the solution difference spectrum (PERY/ANTH‐6‐1 minus PERY) is also shown, with *λ*_max_=600 nm. The absorbance of the difference spectrum is not to scale. See Table 1 for *λ*_max_ values. The concentrations of samples for the solution spectra were ca. 5 mm.

Solution and thin‐film electronic spectra of the ANTH/ANTH‐5‐1, ANTH/ANTH‐6‐1, PYRN/ANTH‐6‐1, and PERY/ANTH‐6‐1 CT complexes are shown in Figures 3 and Figure 4. Solutions, thin‐films, and crystals of the mixtures of PAH donors and PAH(CF_3_)_*n*_ acceptors studied in this work were highly coloured. Photographs of CH_2_Cl_2_ solutions of ANTH/ANTH‐5‐1 and ANTH/ANTH‐6‐1 are shown in Figure S5. Photographs of crystalline ANTH/ANTH‐6‐1, PERY/ANTH‐6‐1, and PERY are also shown in Figure S5. Some colours appeared to be different for the CT complexes in solution and in the solid state. Solutions of ANTH/ANTH‐5‐1 were golden yellow, whereas crystals isolated from these solutions were orange. Solutions of ANTH/ANTH‐6‐1 and ANTH/ANTH‐6‐2 were pink, whereas the crystals were red‐orange and red, respectively. Solutions of CORO/ANTH‐6‐1 were red‐orange, whereas the crystals were violet. The D/A combinations PYRN/ANTH‐6‐1 and PYRN/(TRPH‐6‐1)_2_ were pink both in solution and as crystals, and the D/A complex PYRN/PYRN‐6‐2 was orange both in solution and as crystals. The most pronounced colour change was for a mixture of PERY and ANTH‐6‐1, which was yellow‐green in solution, similar to the colour of PERY, and blue‐green as a solid. A mixture of PYRN and AZUL‐5‐1 formed red‐purple crystals.^46^

**Figure 5:**
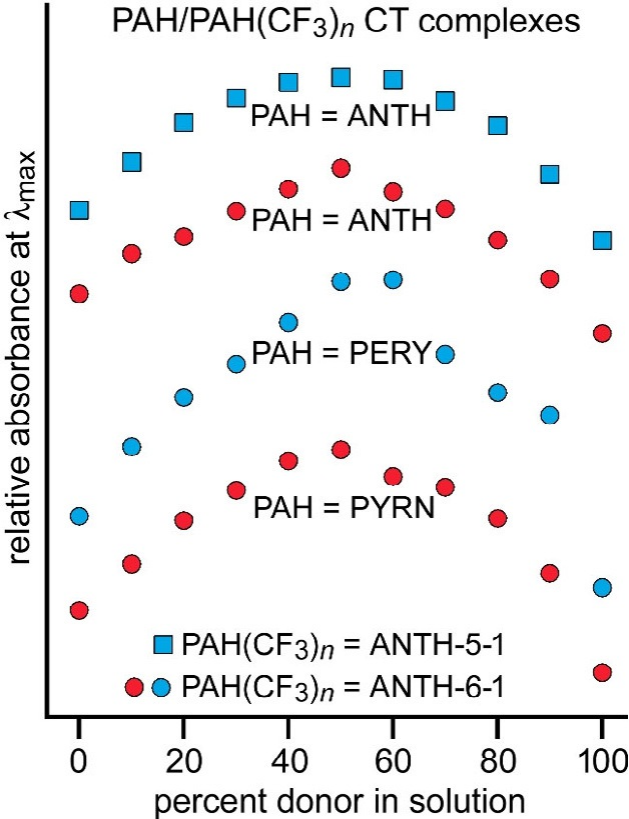
Job plots of percent PAH donor vs. PAH/ANTH(CF_3_)_*n*_ charge‐transfer band *λ*_max_ absorbance for 1,2‐C_2_H_4_Cl_2_ solutions containing mixtures of ANTH, PERY, or PYRN with ANTH‐6‐1 or ANTH‐5‐1. The plots indicate the formation of 1/1 donor/acceptor CT complexes in all four cases under the conditions of the experiments (22 °C; [PAH]+[ANTH(CF_3_)_5,6_]=10 mm). See Table 1 for *λ*_max_ values.

Solution and thin‐film electronic spectra of four of the nine D/A combinations studied in 1,2‐dichloroethane solution and as evaporated drop‐cast thin‐films are shown in Figures 3 and 4. The four combinations are ANTH/ANTH‐5‐1, ANTH/ANTH‐6‐1, PYRN/ANTH‐6‐1, and PERY/ANTH‐6‐1. They are superimposed on the spectra of the individual components. The thin‐film spectra are clearly more complicated, have broader peaks, and in general have different *λ*_max_ values than the solution spectra, behaviour that is similar to other PAH CT complexes with acceptors such as 4‐nitrobenzodifuroxan (NBDF) and tetracyanoethylene (TCNE), as shown in Figure S6. Job plots^89^ for these four combinations of PAH and PAH(CF_3_)_*n*_ are shown in Figure 5, demonstrating that all four combinations formed 1/1 D/A CT complexes in solution. Scott plots^90^ and Seal plots^91^ for titrations of 5.0 mm 1,2‐C_2_H_4_Cl_2_ solutions of ANTH‐5‐1 and ANTH‐6‐1 with concentrations of ANTH that varied from 10 to 70 mm are shown in Figure S7. The CT complex *K*_eq_ and CT band *λ*_max_ extinction coefficients (determined from the slopes of these plots) and the CT band *λ*_max_ values (see also Table 1) are: 1.7(1) M^−1^, 7.7(4) × 10^2^ cm^−1^ m ^−1^, and 467 nm, respectively, for ANTH/ANTH‐5‐1; and 2.8(1) M^−1^, 6.3(3) × 10^2^ cm^−1^ m ^−1^, and 523 nm, respectively, for ANTH/ANTH‐6‐1. Solution electronic spectra of the ANTH/ANTH‐6‐2, CORO/ANTH‐6‐1, PYRN/AZUL‐5‐1, PYRN/PYRN‐6‐2, and PYRN/TRPH‐6‐1 CT complexes are shown in Figure S8. All of the solution electronic spectra were recorded out to 1200 nm. They are shown plotted out to 800 nm in Figures 3, 4, and Figure S8 for simplicity, because no bands were observed between 800 and 1200 nm. As examples, the spectra of PERY/ANTH‐6‐1 and PYRN/ANTH‐6‐1 are also plotted out to 1200 nm in Figure S8.

The CT bands for PYRN/AZUL‐5‐1 and PYRN/PYRN‐6‐2 were masked to a significant extent by other spectral bands, and consequently their *λ*_max_ values are only known approximately (510–540 nm for PYRN/AZUL‐5‐1 and 450–500 nm for PYRN/PYRN‐6‐2; see Table 1). The absence of an obvious charge‐transfer band has also been observed with other azulene charge‐transfer complexes.^92^ In addition to the CT band *λ*_max_ values for all nine (PAH)_*x*_/(PAH(CF_3_)_*n*_)_*y*_ CT complexes, the energy changes that correspond to the *λ*_max_ values (i.e., *E*(*λ*_max_)) are also listed in Table 1. The electronic spectrum of CORO/ANTH‐6‐1 was recorded in toluene because of the limited solubility of CORO in CH_2_Cl_2_ and 1,2‐C_2_H_4_Cl_2_. Based on the CT band *λ*_max_ value solvent effect for piperidine/chloranil and PYRN/chloranil (see Tables S2 and S3),^93^, ^94^ the values listed in Table 1 for CORO/ANTH‐6‐1 are adjusted to 554 nm/2.24 eV instead of the experimental toluene values of 532 nm/2.33 eV in Figure S8.

**Figure 6:**
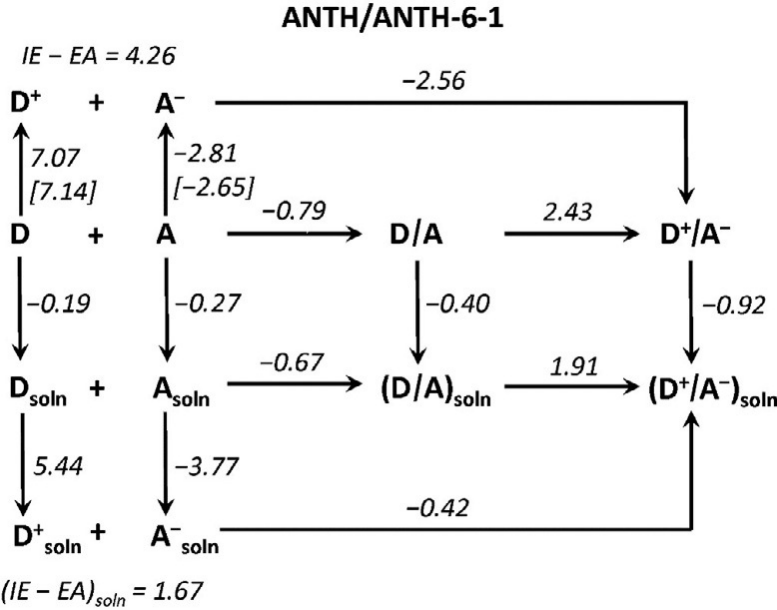
Graphical representation of DFT‐predicted energy changes (eV) for reactions of the ANTH electron donor (D) and ANTH‐6‐1 electron acceptor (A) and their D/A CT complex. The species are either in the gas‐phase (not labelled) or in solution (labelled soln) with a dielectric continuum equivalent to 1,2‐dichloroethane (*ϵ*=10.4). All calculations were B3LYP‐D3/def2‐TZVPP except D/A→D^+^/A^−^ and (D/A)_soln_→(D^+^/A^−^)_soln_, which were B3LYP‐D3/6–311G* because of convergence problems when using the def2‐TZVPP basis set. The *IE* and *EA* values in square brackets are for vertical processes.

DFT calculations were performed to gauge the differences between gas‐phase and solution energy changes for the following transformations (D=PAH; A=PAH(CF_3_)_*n*_; DA=PAH/PAH(CF_3_)_*n*_): D+A→DA; DA→D^+^A^−^; D→D_soln_; A→A_soln_; D_soln_+A_soln_→(DA)_soln_; and (DA)_soln_→(D^+^A^−^)_soln_. A dielectric continuum equivalent to the 10.4 dielectric constant of 1,2‐C_2_H_4_Cl_2_ was used (the dielectric constant of CH_2_Cl_2_ is similar, 9.08). These and additional computational results are listed in Table S4 for all nine PAH/PAH(CF_3_)_*n*_ CT complexes and are shown graphically for ANTH/ANTH‐6‐1 in Figure 6. Similar figures for the other PAH/PAH(CF_3_)_*n*_ CT complexes are shown in Figure S9. A subset of the calculations for all nine CT complexes are listed in Table 2.

**Table 2:** Selected DFT Computational Results.^[a]^

CT complex	D+A→D/A	D/A→D^+^A^‐^	D→D_soln_	A→A_soln_	D_soln_+A_soln_→(D/A)_soln_	(D/A)_soln_→(D^+^/A^−^)_soln_
ANTH/ANTH‐5‐1	−0.74	2.70^[b]^	−0.19	−0.27	−0.63	2.16^[b]^
ANTH/ANTH‐6‐1	−0.79	2.43^[b]^	−0.19	−0.33	−0.67	1.91^[b]^
ANTH/ANTH‐6‐2	−0.82	2.52^[b]^	−0.19	−0.29	−0.72	1.96^[b]^
CORO/ANTH‐6‐1	−1.03	2.57^[b]^	−0.25	−0.33	−0.87	2.11^[b]^
PERY/ANTH‐6‐1	−0.97	2.09^[b]^	−0.26	−0.33	−0.79	1.59^[b]^
PYRN/ANTH‐6‐1	−0.83	2.54^[b]^	−0.20	−0.33	−0.67	2.03^[b]^
PYRN/AZUL‐5‐1	−0.78	2.68^[b]^	−0.20	−0.29	−0.68	2.15^[b]^
PYRN/PYRN‐6‐2	−0.82	2.73^[b]^	−0.20	−0.30	−0.69	2.20^[b]^
PYRN/TRPH‐6‐1	−0.91	3.30^[b]^	−0.20	−0.41	−0.78	2.82^[b]^

[a] All energies in eV. All calculations at the B3LYP‐D3/def2‐TZVPP level of theory unless otherwise indicated. [b] Calculations at the B3LYP‐D3/6‐311G* level of theory because of convergence problems when using the def2‐TZVPP basis set. All of the DFT results are listed in Table S4 (in which the generic acronyms D/A and D^+^/A^‐^ are abbreviated DA and D^+^A^‐^).

### Structures of (PAH)_*x*_/(PAH(CF_3_)_*n*_)_*y*_ CT complex co‐crystals

Data collection and final refinement parameters for the single‐crystal X‐ray structures of the seven (PAH)_*x*_/(PAH(CF_3_)_*n*_)_*y*_ CT complex co‐crystals reported in this work are listed in Table 3. The ratio of donors to acceptors, *x*/*y*, is 1/1 for four of the structures, 1/2 for two structures, and 2/1 for one structure. Relevant geometric parameters for the D_*x*_/A_*y*_ structures are listed in Table 4, including, for comparison, the corresponding parameters for the previously reported 1/1 structures of PYRN/AZUL‐5‐1^46^ and ANTH/NAPH(F)_8_,^44^ and the 1/2 structure of PYRN/(ANTH‐6‐1)_2_.^47^

**Table 3:** Crystal Data and Final Refinement Parameters for the (PAH)_*x*_/(PAH(CF_3_)_*n*_)_*y*_ Co‐crystal X‐ray Diffraction Structures.^[a]^

compound^[a]^	ANTH/(ANTH‐5‐1)_2_	ANTH/ANTH‐6‐1	ANTH/ANTH‐6‐2	PERY/ANTH‐6‐1	PYRN/(TRPH‐6‐1)_2_	PYRN/PYRN‐6‐2	(CORO)_2_/ANTH‐6‐1
formula	C_26_H_10_F_15_	C_34_H_14_F_18_	C_34_H_14_F_18_	C_40_H_16_F_18_	C_32_H_11_F_18_	C_38_H_14_F_18_	C_68_H_28_F_18_
formula wt. [g mol^−1^]	607.34	764.45	764.45	838.53	737.41	812.49	1186.90
colour	orange	red‐orange	red	blue‐green	pink	orange	violet
X‐ray wavelength [Å]	0.71073	0.71073	0.41328	0.41328	0.41328	0.71073	0.41328
crystal system	triclinic	triclinic	monoclinic	monoclinic	triclinic	monoclinic	triclinic
space group, *Z*	*P* $\bar{1}$ , 2	*P* $\bar{1}$ , 1	*P*2_1_/*n*, 4	*P*2_1_/*n*, 2	*P* $\bar{1}$ , 2	*P*2_1_/*n*, 8	*P* $\bar{1}$ , 1
*a* [Å]	10.4208(8)	7.3205(3)	7.3334(4)	7.5458(3)	10.5246(3)	14.2327(6)	10.6233(6)
*b* [Å]	11.2016(9)	9.5075(3)	22.6954(11)	9.9934(3)	11.6583(4)	18.7253(8)	10.6247(6)
*c* [Å]	12.4222(10)	10.6112(4)	17.674(1)	20.5503(7)	11.9258(4)	23.9391(11)	11.5482(7)
α [°]	91.813(4)	80.751(2)	90	90	95.673(1)	90	96.506(1)
β [°]	114.299(4)	88.391(2)	101.947(1)	94.108(1)	93.026(1)	107.200 (2)	109.548(1)
γ [°]	117.018(4)	78.520(2)	90	90	113.015(1)	90	101.923(1)
*V* [Å^3^]	1133.89(17)	714.35(5)	2877.9(3)	1545.68(9)	1333.52(8)	6094.7(5)	1178.05(12)
*ρ*_calc_ [g cm^−3^]	1.779	1.777	1.764	1.802	1.836	1.771	1.673
*T* [K]	120(1)	120(1)	100(1)	100(1)	100(1)	120(1)	100(1)
min/max e density [e^−^ Å^−3^]	−0.447/0.511	−0.266/0.524	−0.259/0.351	−0.232/0.374	−0.246/0.390	−0.300/0.374	−0.281/0.416
total/unique reflections	4903/3725	5364/4350	6448/4487	3670/3204	7403/6394	14 327/9377	8309/6936
parameters/restraints	370/0	235/0	469/444	262/210	507/66	1125/72	388/375
*R*(*F*) (*I*>2*σ*(*I*))^[b]^	0.0532	0.0417	0.0570	0.0359	0.0359	0.0423	0.0378
*wR*(*F* ^*2*^) [all data]^[b]^	0.1382	0.1181	0.1373	0.1108	0.1016	0.1036	0.1143
GOF	1.030	1.048	0.995	1.102	1.023	1.013	1.031
CCDC number	1922642	1922643	1922648	1922645	1922647	1922646	1922644

[a] Abbreviations: ANTH=anthracene; CORO=coronene; PERY=perylene; PYRN=pyrene; ANTH‐5‐1=1,3,6,8,10‐ANTH(CF_3_)_5_; ANTH‐6‐1=2,3,6,7,9,10‐ANTH(CF_3_)_6_; ANTH‐6‐2=1,2,3,6,8,10‐ ANTH(CF_3_)_6_; PYRN‐6‐2=1,2,4,6,8,9‐PYRN(CF_3_)_6_; TRPH‐6‐1=1,3,6,7,10,11‐TRPH(CF_3_)_6_. [b] *R*(*F*)=Σ∥*F*_o_|−|F_c_∥/ Σ|*F*_o_|; *wR*(*F* ^*2*^)=(Σ[*w*(*F*_o_^2^−*F*_c_^2^)^2^]/ Σ[*w*(*F*_o_^2^)^2^])^1/2^.

**Table 4:** Crystal Structure Geometric Parameters for (PAH)_*x*_/(PAH(CF_3_)_*n*_)_*y*_ Co‐crystal X‐ray Diffraction Structures.^[a]^

structure	major axis, Å; ∡ to ⊙⋅⋅⋅⊙	minor axis, Å; ∡ to ⊙⋅⋅⋅⊙	D/A LSP dihedral ∡	A→D(LSP) OOPs (i.e., π–π overlap), Å^[b]^	rotation of D/A major axes	D major axis slip, Å [%]	D minor axis slip, Å [%]
ANTH/(ANTH‐5‐1)_2_	D: 7.296; 14.8°	D: 2.784; 7.6°	2.2°	3.43–3.54 [7: 3.49]	17.1°	2.42 [33]	1.07 [38]
	A: 7.301; 13.4°	A: 2.800; 11.2°					
ANTH/ANTH‐6‐1	D: 7.296; 17.5°	D: 2.809; 3.0°	2.4°	3.47–3.66 [5: 3.55]	20.7°	2.20 [30]	0.44 [16]
	A: 7.344; 16.2°	A: 2.832; 5.9°					
ANTH/ANTH‐6‐2	D: 7.301; 17.7°	D: 2.804; 3.6°	1.9°	3.45–3.69 [6: 3.52]	14.8°	2.24 [31]	0.49 [18]
	A: 7.342; 16.2°	A: 2.806; 0.7°		3.49–3.62 [6: 3.54]			
(CORO)_2_/ANTH‐6‐1	D: 7.354; 3.5°	D: 5.682; 22.4°	4.9°	3.43–3.75 [11: 3.61]	2.6°	0.10 [1.3]^[c]^	3.09 [54]^[c]^
	A: 7.353; 2.2°	A: 2.844; 21.8°				0.81 [11]^[d]^	1.17 [21]^[d]^
PERY/ANTH‐6‐1	D: 5.727; 7.2°	D: 2.493; 18.9°	1.0°	3.48–3.66 [9: 3.55]	23.2°	0.89 [16]	2.42 [97]
	A: 7.353; 0.7°	A: 2.849; 15.1°					
PYRN/(ANTH‐6‐1)_2_^e^	D: 7.006; 6.7°	D: 4.919; 3.4°	1.6°	3.36–3.73 [10: 3.56]	33.0°	–^[e]^	–^[e]^
	A: 7.300; 11.6°	A: 2.816; 0.5°					
PYRN/AZUL‐5‐1^[f]^	D: 7.027; 2.3°	D: 4.917; 3.9°	0.8°	3.58–3.65 [7: 3.61]	3.9°	0.29 [4.1]	0.50 [10]
	A: 5.236; 2.3°	A: 3.164; 4.4°					
PYRN/PYRN‐6‐2	D: 7.009; 0.1°	D: 4.904; 0.6°	3.1°;	3.41–3.59 [10: 3.50]	22.8°;	–^[g]^	–^[g]^
	A: 7.041; 0.2°	A: 4.900; 4.4°	3.1°	3.47–3.71 [9: 3.58]	85.2°		
PYRN/(TRPH‐6‐1)_2_	D: 7.020; 3.7°	D: 4.914; 29.9°	4.5°	3.37–3.73 [8: 3.54]	–^[h]^	0.78 [11]	5.96 [121]
	–^[h]^	–^[h]^					
ANTH/NAPH(F)_8_^i^	D: 7.296; 14.8°	D: 2.784; 7.6°	2.7°	3.31–3.40 [6: 3.35]	19.9°	D: 0.26 [3.5]	D: 1.07 [38]
	A: 7.301; 9.3°	A: 2.800; 12.9°				A: 0.12 [2.5]	A: 1.08 [26]

[a] All data from this work unless otherwise indicated. [b] The range of out‐of‐plane displacements (OOPs), the number of OOPs, and average OOP for acceptor C(sp^2^) atoms from the least‐squares plane of donor C(sp^2^) atoms. [c] For CORO molecules on either side of the ANTH‐6‐1 acceptor. [d] For the CORO pair between two ANTH‐6‐1 acceptors. [e] Ref. 47. Structure contains acceptor‐donor‐acceptor isolated triads; donor‐to‐donor major/minor axis slips are not defined. [f] Ref. 46. The AZUL‐5‐1 acceptor minor axis is taken as longest distance across 7‐membered ring perpendicular to major axis. [g] Major and minor axis slip dimensions are not defined in this case because the PYRN donor molecules on either side of the acceptor are rotated so that their major axes are almost perpendicular (86.1°). [h] The TRPH‐6‐1 acceptor does not have clearly‐defined major and minor axes. [i] Ref. 44.

**Figure 7:**
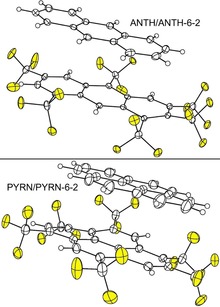
Thermal ellipsoid plots of a single donor/acceptor pair in the CT complex co‐crystal structures of ANTH/ANTH‐6‐2 (top) and PYRN/PYRN‐6‐2 (bottom; 50 % probability ellipsoids except for H atoms; F atoms shaded yellow). Both structures exhibit hexagonal arrays of infinite alternating D/A/D/A. stacks of nearly‐parallel donors and acceptors.

**Figure 8:**
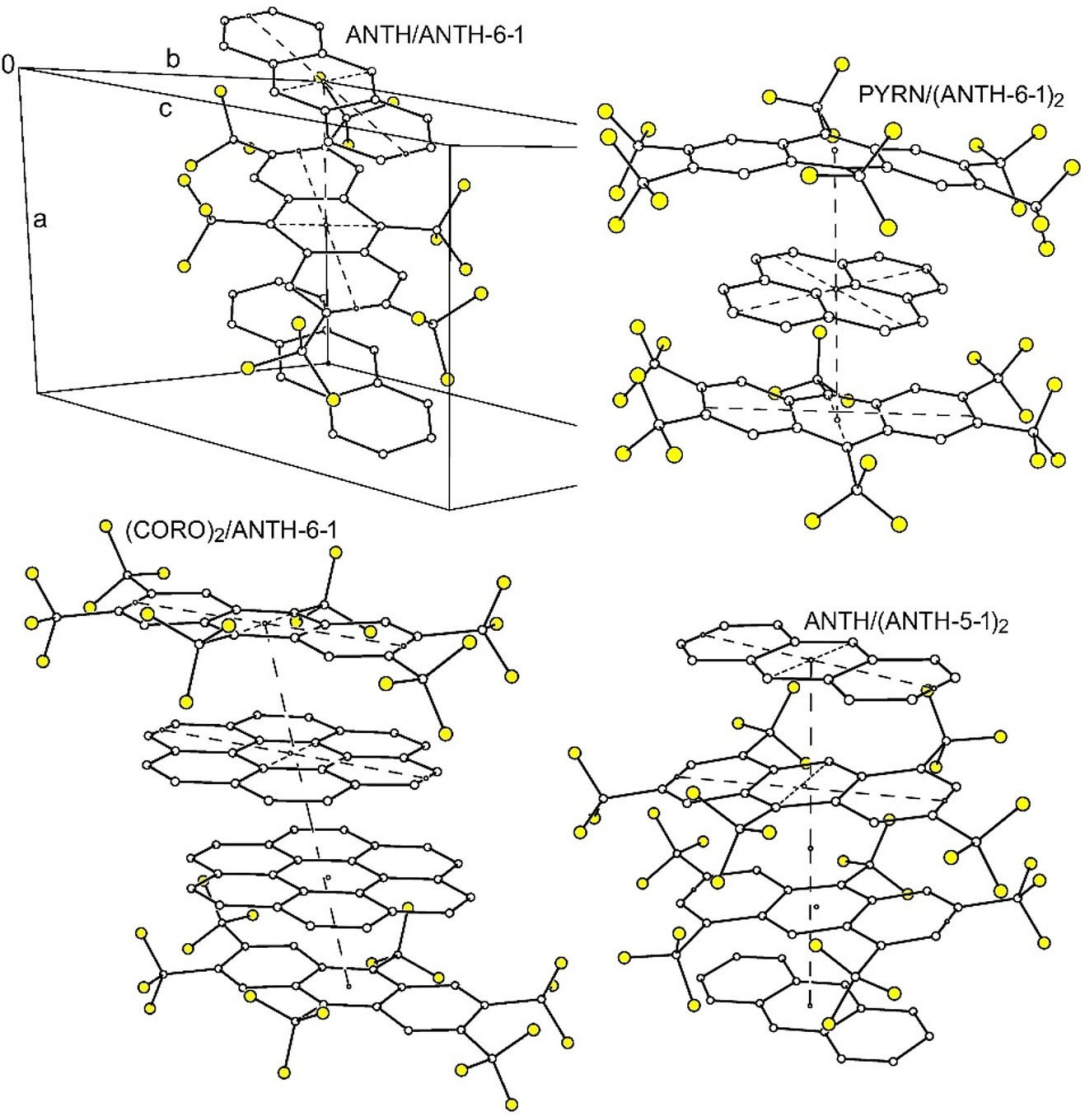
Drawings of portions of the structures of (clockwise from top left) ANTH/ANTH‐6‐1, PYRN/(ANTH‐6‐1)_2_ (first reported in ref. 47), ANTH/(ANTH‐5‐1)_2_, and (CORO)_2_/ANTH‐6‐1 showing the nearly‐parallel donors and acceptors (H atoms omitted for clarity; F atoms shaded yellow). The smallest circles represent either bond or hexagon centroids. The dihedral angles between the least‐squares planes of the aromatic cores of each donor/acceptor pair of molecules are 2.4°, 1.6°, 2.2°, and 4.9°, respectively. The major and minor axes are shown as dashed lines. The rotations of the donor major axes with respect to the acceptor major axes are 20.7°, 33.0°, 17.1°, and 2.6°, respectively.

Thermal ellipsoid plots for one D/A pair in ANTH/ANTH‐6‐2 and PYRN/PYRN‐6‐2, the CT complex co‐crystal structures with the two new PAH(CF_3_)_6_ acceptors, are shown in Figure 7. Thermal ellipsoid plots for the other structures are shown in Figures S10–S12. All of the structures exhibit nearly‐parallel donor and acceptor nearest neighbours with significant π–π overlap. This is shown for ANTH/(ANTH‐5‐1)_2_, ANTH/ANTH‐6‐1, PYRN/(ANTH‐6‐1)_2_, and (CORO)_2_/ANTH‐6‐1 in Figure 8. Similar drawings for the other five (PAH)_*x*_/(PAH(CF_3_)_*n*_)_*y*_ co‐crystal structures are shown in Figure S13.

**Figure 9:**
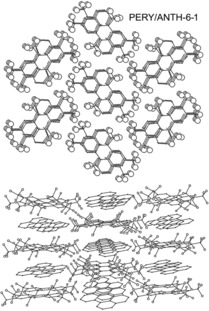
Two perpendicular views of the hexagonal array of infinite alternating D/A/D/A. stacks of PERY (D) and ANTH‐6‐1 (A) molecules in the CT complex co‐crystal structure of PERY/ANTH‐6‐1 (H atoms omitted for clarity; the larger spheres in both drawings are F atoms).

Eight of the nine (PAH)_*x*_/(PAH(CF_3_)_*n*_)_*y*_ structures consist of hexagonal arrays of infinite stacks of nearly‐parallel donors and acceptors (the largest dihedral angle between the donor and acceptor least‐squares planes of C(sp^2^) atoms is 4.9° in (CORO)_2_/ANTH‐6‐1 and the smallest is 0.8° in PYRN/AZUL‐5‐1). Two perpendicular views of this packing motif for PERY/ANTH‐6‐1 are shown in Figure 9. Similar pairs of drawings for the seven other structures with this packing motif are shown in Figures S14–S20. In all but one of these eight structures, molecules in neighbouring stacks are parallel (i.e., the least‐squares planes of the PAH donors in all neighbouring stacks are parallel to one another, as are the least‐squares planes of the PAH(CF_3_)_*n*_ acceptors in all neighbouring stacks). The one departure from this motif is the structure of ANTH/ANTH‐6‐2, in which molecules in half of the stacks are tilted 34.6° relative to the molecules in the other stacks. The stacking direction is the crystallographic *a*‐axis in ANTH/ANTH‐6‐1, ANTH/ANTH‐6‐2, PERY/ANTH‐6‐1, PYRN/AZUL‐5‐1, and PYRN/PYRN‐6‐2, the crystallographic *c*‐axis in (CORO)_2_/ANTH‐6‐1 and PYRN(TRPH‐6‐1)_2_, and the (1 1 0) direction in ANTH/(ANTH‐5‐1)_2_. The donor and acceptor C(sp^2^) least‐squares planes in the eight structures are not perpendicular to the stacking direction. Instead, both the major and minor axes of the donors and the acceptors are tilted with respect to the stacking direction, by as little as 0.1° or as much as 29.9°. These angles are listed in Table 4.

One of the nine (PAH)_*x*_/(PAH(CF_3_)_*n*_)_*y*_ structures, PYRN/(ANTH‐6‐1)_2_,^47^ consists of discrete, centrosymmetric {ANTH‐6‐1/PYRN/ANTH‐6‐1} ({A/D/A}) triplet complexes of nearly parallel molecules (the donor/acceptor least‐squares‐planes dihedral angle is 1.6°). The {A/D/A} complex is shown in Figure 8 and will be compared to the other structures in a later section.

**Figure 10:**
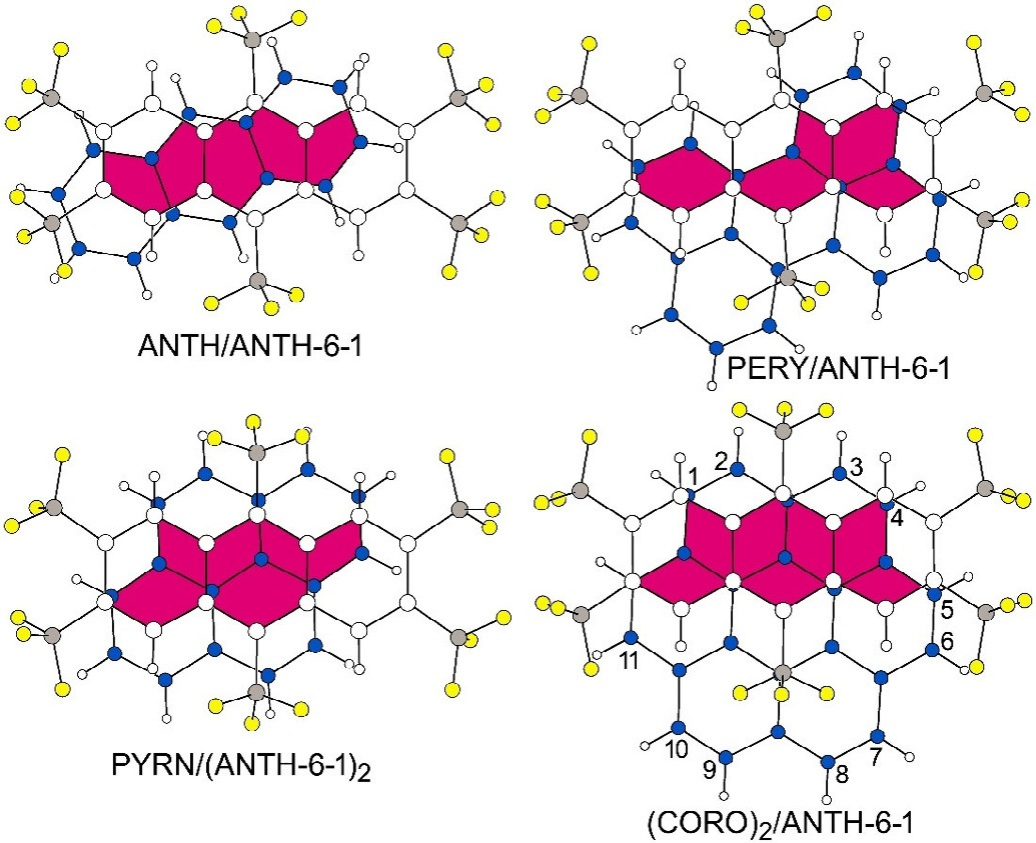
Parallel projection drawings of the π–π overlap (shaded magenta) of neighbouring pairs of PAH donors and an ANTH‐6‐1 acceptor in the co‐crystal structures of (clockwise from upper left) ANTH/ANTH‐6‐1, PERY/ANTH‐6‐1, (CORO)_2_/ANTH‐6‐1, and PYRN/(ANTH‐6‐1)_2_. In all drawings F atoms are shaded yellow, ANTH‐6‐1 C(sp^3^) atoms are shaded grey, PAH donor C(sp^2^) atoms are shaded blue, and the least squares planes of the PAH donor C(sp^2^) atoms are in the plane of the page. Integers 1–11 in the (CORO)_2_/ANTH‐6‐1 drawing indicate the C(sp^2^) locants (C12 is blocked from view).

There is significant π–π overlap between the nearly‐parallel PAH donors and PAH(CF_3_)_*n*_ acceptors in each stack, as well as between the three molecules in the {ANTH‐6‐1/PYRN/ANTH‐6‐1} triplet complexes, the CORO/CORO nearest neighbours in (CORO)_2_/ANTH‐6‐1, the ANTH‐5‐1/ANTH‐5‐1 nearest neighbours in ANTH/(ANTH‐5‐1)_2_, and the TRPH‐6‐1/TRPH‐6‐1 nearest neighbours in PYRN/(TRPH‐6‐1)_2_. This is shown in Figure 10 for one D/A nearest‐neighbour pair in the crystal structures of ANTH/ANTH‐6‐1, (CORO)_2_/ANTH‐6‐1, PERY/ANTH‐6‐1, and PYRN/(ANTH‐6‐1)_2_. Similar drawings for the other CT complex co‐crystal structures are shown in Figures S21–S24. A comparison of the D/A π–π overlap for the four structures with PYRN as the donor is shown in Figure S25. A comparison of the A/A π–π overlap for the (TRPH‐6‐1)_2_ pairs in the structure of PYRN/(TRPH‐6‐1)_2_ and in the structure of TRPH‐6‐1^26^ is shown in Figure S26.

## Discussion

### Synthesis and characterization of ANTH‐6‐2 and PYRN‐6‐2

The new PAH(CF_3_)_6_ derivatives were prepared by heating ANTH or PYRN in a sealed glass ampoule in the presence of CF_3_ radicals, formed by dissociation of gaseous CF_3_I at 360 °C. This procedure results in a mixture of ANTH(CF_3_)_*n*_ or PYRN(CF_3_)_*n*_ derivatives in which the predominant products are isomers with *n=*5 and 6 that can be separated by HPLC.^26^

The purity of the samples of ANTH‐6‐2 and PYRN‐6‐2 used in this work were judged to be 98+ mol % based on their ^1^H and ^19^F NMR spectra. The 16±1 Hz ^5^ *J*_FF_ and ^6^ *J*_FF_ quartets or quartets‐of‐quartets observed in the ^19^F spectra are due to through‐space Fermi contact coupling of proximate, rapidly rotating CF_3_ groups with F⋅⋅⋅F distances ≤3 Å.^95^, ^96^, ^97^, ^98^, ^99^, ^100^ There are several 2.5–2.8 Å F⋅⋅⋅F distances between the CF_3_ groups attached to C1, C2, and C3 in the crystal structure of ANTH/ANTH‐6‐2 and between the CF_3_ groups attached to C1 and C2 and attached to C8 and C9 in the crystal structure of PYRN/PYRN‐6‐2 (see Figure 1 for locant positions).

Of particular importance in the discussion that follows are *IE*s and *E*_1/2_(+/0) values of PAH electron donors, *EA*s and *E*_1/2_(0/−) values of PAH(CF_3_)_*n*_ and other electron acceptors, the energies of D/A complex charge transfer in solution (i.e., the energy of the electronic spectrum CT band *λ*_max_, which is abbreviated *E*(*λ*_max_)), and the quantity [*IE*(donor)−*EA*(acceptor)], which is abbreviated Δ(*IE*/*EA*). Cyclic voltammetry *E*_1/2_(0/−) values were determined for the new acceptors ANTH‐6‐2 and PYRN‐6‐2 (−0.98 and −1.05 V vs. Fe(Cp)_2_^+/0^_,_ respectively). The *EA* of ANTH‐6‐2 was determined by LT‐PES to be 2.68(2) eV, but technical difficulties prevented the determination of the *EA* of PYRN‐6‐2. Based on the differences between the *EA*s of ANTH‐6‐1 and ANTH‐6‐2 and their *E*_1/2_(0/−) values, as well as the *EA*s and *E*_1/2_(0/− values for other PAH(CF_3_)_*n*_ isomers listed in Table S1, we estimate, with a generous margin of uncertainty, that the *EA* of PYRN‐6‐2 is between 2.6 and 2.7 eV, and this is the *EA* range listed for PYRN‐6‐2 in Table 1.

### Correlations of *IE*s and *EA*s with *E*_1/2_ values

**Figure 11:**
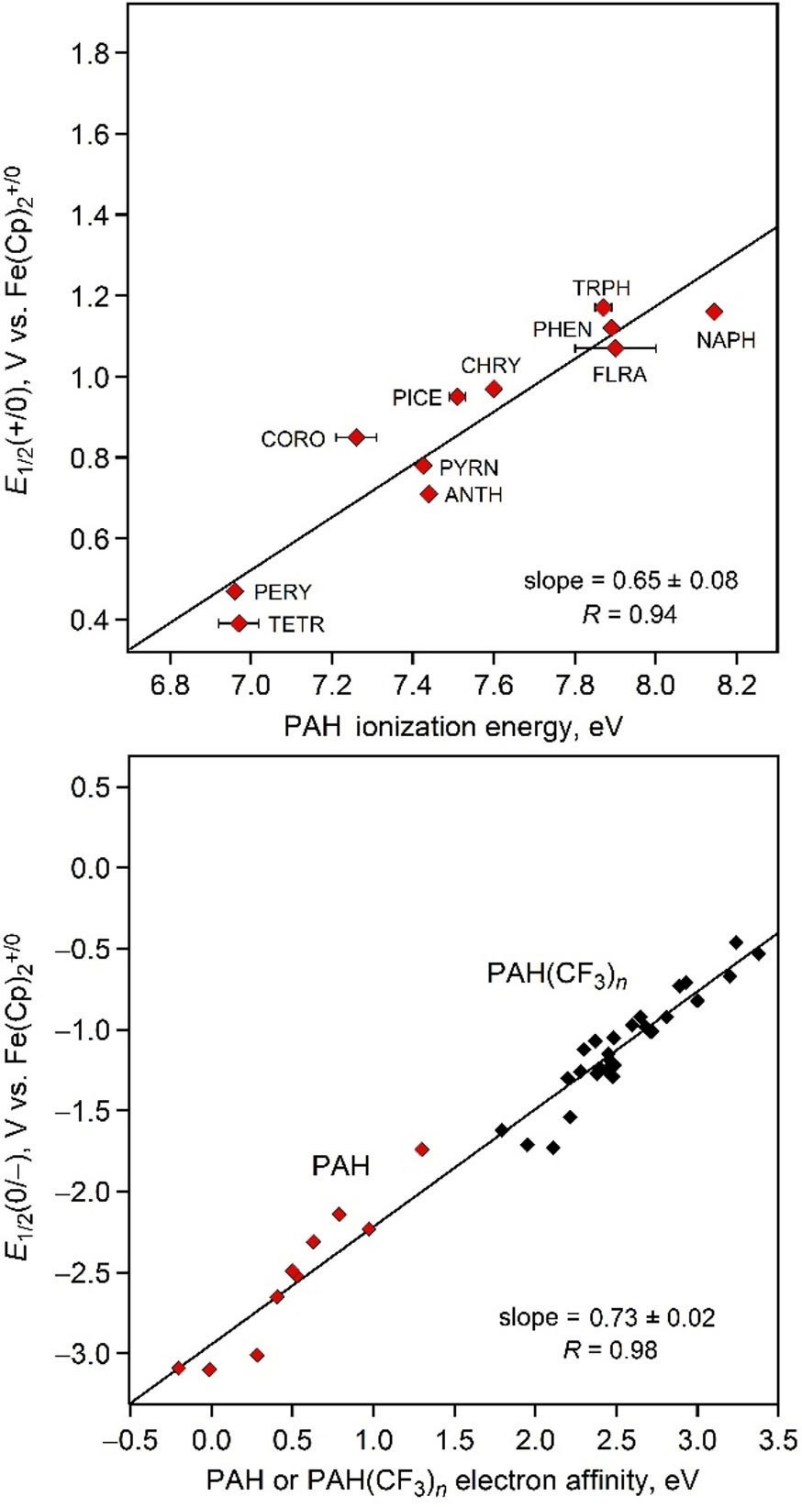
Top: Plot of the *E*_1/2_(+/0) values for PAHs reported in ref. 65 (CHRY=chrysene; PICE=picene) vs. the most recent and most reliable PAH *IE*s (see Table S5 for references). Bottom: Plot of PAH and PAH(CF_3_)_*n*_ experimental *EA* vs. *E*_1/2_(0/−) values. Both plots show that differences in gas‐phase one‐electron reductions or gas‐phase one‐electron oxidations for these sets of compounds are attenuated in solution, on average, by 27–35 %. Note that the lengths and ranges of values on both axes in both plots are equal (i.e., these are square plots), to show visually and unambiguously that the slopes are significantly less than unity).

Table S5 lists the most recent and most precise *EA*s and *IE*s for 12 PAHs (including the ones that are also listed in Table 1). The upper part of Figure 11 is a plot of the polarographic *E*_1/2_(+/0) values in CH_3_CN reported by Pysh and Yang in their classic 1963 study^65^ versus the PAH *IE*s listed in Table S5. A linear least‐square fit to the data has a slope of 0.65±0.08 V eV^−1^ (the slope in Pysh and Yang's original plot, which did not include the PAHs in Figure 11 other than NAPH, ANTH, and phenanthrene (PHEN), and which used photoionization energies available at that time, had a slope of 0.68±0.01 V eV^−1^). A similar plot of *E*_1/2_(+/0) values in CH_3_CN for 9‐ANTH(X) and 9,10‐ANTH(X)_2_ derivatives versus their photoionization energies, shown in Figure S27, has a slope of 0.73±0.07 V eV^−1^.^101^ A related plot for alkyl‐substituted benzenes (not shown) has a slope of 0.71 V eV^−1^.^102^ A plot of electrochemical oxidation potentials versus *IE*s for eight PAHs published by Savéant et al. has a slope of 0.85 V eV^−1^.^103^ It is clear that differences in gas phase *IE*s for PAHs are significantly attenuated in solution when measured by electrochemical oxidation. Nevertheless, it is possible to find papers in which a 1:1 correlation was assumed and/or was found for other compounds, possibly because electrochemical oxidation peak potentials were used when quasi‐reversible *E*_1/2_(+/0) values were not available.^104^, ^105^, ^106^ The bottom line is that solvation not only determines the intercepts of linear fits to *E*_1/2_(+/0) versus *IE* plots, it also reduces, or attenuates, the intrinsic differences in PAH *IE* or *E*(HOMO) values. For any pair of compounds, the difference in their *E*_1/2_(+/0) values should not be assumed to be equal to the difference in their *IE*s.

A similar attenuation of solution versus gas‐phase energy changes is expected, and has been found, for reductions of PAHs, that is, for plots of *E*_1/2_(0/−) versus *EA*s. However, it is rare to find experimental *EA*s and the corresponding experimental *E*_1/2_(0/−) values for the same set of PAHs, and it is rarer still to find sets of data that were measured with the same instrumentation and under the same conditions in the same laboratory. For that reason, calculated *EA*s and either calculated or “corrected” experimental *E*_1/2_(0/−) values are often used, and it is not uncommon to find plots with slopes of 0.9–1.0 V eV^−1^.^107^, ^108^, ^109^, ^110^, ^111^, ^112^ In contrast, a recent paper by Calbo, Aragó, et al. reported calculated *EA*s for a variety of aromatic molecules and strong electron acceptors, and a plot (prepared for this work) of experimental *E*_1/2_(0/−) values versus their G3(MP2) *EA*s has a slope of 0.75±0.04 V eV^−1^, as shown in Figure S28 (neither the plot nor the magnitude of the slope of the linear correlation was included in their paper).^113^ They reported that their calculated *EA*s and the experimental *E*_1/2_(0/−) values are related by an additive correction equal to the difference in the free energy of solvation between the neutral and anionic species (ΔΔ*G*_solv_). However, they also showed that ΔΔ*G*_solv_ is not a constant even for a family of related compounds.

The lower part of Figure 11 is a plot of experimental *E*_1/2_(0/−) values for PAH compounds measured at CSU using the same solvent, electrolyte salt, electrode array, electrochemical internal standard, and potentiostat versus experimental PAH *EA*s from the literature and PAH(CF_3_)_*n*_ *EA*s measured with the same LT‐PES instrumentation at Pacific Northwest National Laboratory. There are 38 data points in the plot, 10 for PAHs and 28 for PAH(CF_3_)_*n*_ compounds with *n=*2–6 (most of the individual values are listed in Tables S5 and S6^22^, ^25^, ^26^, ^46^, ^49^, ^50^, ^51^, ^114^). The ranges of *E*_1/2_(0/−) values and *EA*s are 2.64 V and 3.58 eV, respectively. The slope of the linear least‐squares fit is 0.73±0.02 V eV^−1^. The maximum deviation from the regression line is 0.32 V (for TRPH‐6‐1), and the average deviation is 0.10 V. To our knowledge, there are no comparable sets of experimental *EA*s and *E*_1/2_(0/−) values in the literature, let alone for a structurally similar set of compounds (in this case PAHs and aza‐PAHs with only H and CF_3_ substituents). As in the case for solution versus gas‐phase oxidations, there is no doubt that differences in gas phase *EA*s for PAHs and PAH(CF_3_)_*n*_ derivatives are significantly attenuated, in this case by ca. 27 %, when measured in solution by electrochemical reduction (i.e., for any pair of these compounds, the difference in their electron‐acceptor ability in solution is, on average, only 73 % of the difference in their electron‐acceptor ability in the gas phase).

### PAH/PAH(CF_3_)_*n*_ CT complexes in solution

#### Donor/acceptor complex stoichiometry and equilibrium constants

The results plotted in Figure 5 demonstrate that four of the nine PAH/PAH(CF_3_)_*n*_ combinations studied in this work formed 1/1 CT complexes in 1,2‐C_2_H_4_Cl_2_ solution; even the two combinations that formed 1/2 co‐crystals, ANTH/(ANTH‐5‐1)_2_ and PYRN/(ANTH‐6‐1)_2_. It is likely that the other five combinations also form 1/1 CT complexes in solution, but this was not specifically investigated. The *EA*s for ANTH‐5‐1 and ANTH‐6‐1 are 2.40(2) and 2.81(2) eV, respectively. The *K*_eq_ values for the ANTH/ANTH‐5‐1 and ANTH/ANTH‐6‐1 CT complexes in 1,2,C_2_H_4_Cl_2_ are 1.7(1) m ^−1^ and 2.8(1) m ^−1^, respectively. These CT equilibrium constants can be compared with the following data from the literature: the *EA* of chloroanil is 2.78(6) eV,^42^ and the *K*_eq_ reported for the ANTH/chloroanil CT complex in CH_2_Cl_2_ is 1.0 m ^−1^ (in CHCl_3_ and CCl_4_ the values are 1.7 and 3.1 m ^−1^, respectively);^115^ the *EA* of NBDF was estimated to be 2.45 eV (but see Table S6),^74^ and the *K*_eq_ reported for the ANTH/NBDF CT complex in CHCl_3_ is 5.5(3) m ^−1^;^74^ the *EA* of tetrachlorophthalic anhydride (TCPA) is 1.95(9) eV,^116^ and the *K*_eq_ reported for the ANTH/TCPA CT complex in CCl_4_ is 8.75 m ^−1^.^91^, ^117^ These data are listed in Tables S6 and S7. There is no correlation between *K*_eq_ and *EA*. In fact, the *K*_eq_ values differ by only a factor of 8.8, which is equivalent to a ΔΔ*G* value of 0.08 eV, while the range of *EA* values is 0.86 eV (If the factor of 3 solvent effect for CH_2_Cl_2_ vs. CCl_4_ reported by Lofti and Roberts is taken into account, the intrinsic *K*_eq_ values only differ by a factor of 2.9). However, differences in acceptor *EA* would not be expected to affect the energy of association for D/A complexes with a common donor if there is negligible charge transfer in the D/A complex ground state. On the other hand, given the differences in size and shape of the π‐systems of the five acceptors in this comparison, it is noteworthy that the *K*_eq_ values are so similar.

As far as the *K*_eq_ factor of ca. 2 for the formation of ANTH/ANTH‐6‐1 versus the formation of ANTH/ANTH‐5‐1, note that the difference in DFT Δ*E*_solv_ values for the D/A complex formation equilibria is 0.04 eV in favour of ANTH/ANTH‐6‐1. The DFT results, listed in Table 2 and Table S4 and shown graphically in Figure 6 and Figure S9, also show the expected results with respect to donor and acceptor solvation energies as a function of molecular size in a dielectric continuum equivalent to 1,2‐C_2_H_4_Cl_2_. The smaller donors, ANTH and PYRN, have solvation energies of −0.19 or −0.20 eV, and the larger donors, PERY and CORO, have solvation energies of −0.26 or −0.25 eV. The smaller acceptors, AZUL‐5‐1 and ANTH‐5‐1, have solvation energies of −0.29 or −0.27 eV, and the largest acceptor, TRPH‐6‐1, has a solvation energy of −0.41 eV. For the four donors ANTH, PYRN, PERY, and CORO and the common acceptor ANTH‐6‐1, the D+A→D/A energy change increases as the size of the donor increases, from ANTH (−0.79 eV) to PYRN (−0.83 eV) to PERY (−0.97 eV) to CORO (−1.03 eV), and not as a monotonic function of donor *IE*. Similarly, for the four acceptors AZUL‐5‐1, ANTH‐6‐1, PYRN‐6‐2, and TRPH‐6‐1 and the common donor PYRN, the D+A→D/A energy change increases as the size of the acceptor increases, from AZUL‐5‐1 (−0.78 eV) to ANTH‐6‐1 (−0.83 eV) and PYRN‐6‐2 (−0.82 eV) to TRPH‐6‐1 (−0.91 eV), and not as a monotonic function of acceptor *EA*. These results indicate that the PAH/PAH(CF_3_)_*n*_ CT complexes studied in this work have negligible charge‐transfer character in their electronic ground states.

#### Correlations of IEs and EAs with E(λ_max_) values

It has been known since Mulliken's seminal work on CT complexes of neutral donors and acceptors that the energy of the CT transition in solution (*E*(*λ*_max_)) is proportional to 1) the vertical *IE* of the donor for CT complexes with a given acceptor, 2) the vertical *EA* of the acceptor for CT complexes with a given donor, and, consequently, 3) the difference between the donor *IE* and the acceptor *EA*.^118^, ^119^, ^120^, ^121^, ^122^, ^123^ (Few vertical *IE*s and *EA*s are known relative to the greater number of precise adiabatic *IE*s and *EA*s; fortunately, as far as this work is concerned, the vertical and adiabatic *IE*s for PAHs are generally the same to within 0.1 or 0.2 eV.^124^) What has not always been appreciated,^94^, ^117^, ^125^, ^126^, ^127^, ^128^ even in the 21st century,^74^, ^111^, ^129^, ^130^ is that in general the proportionality constants are not 1.0.

**Figure 12:**
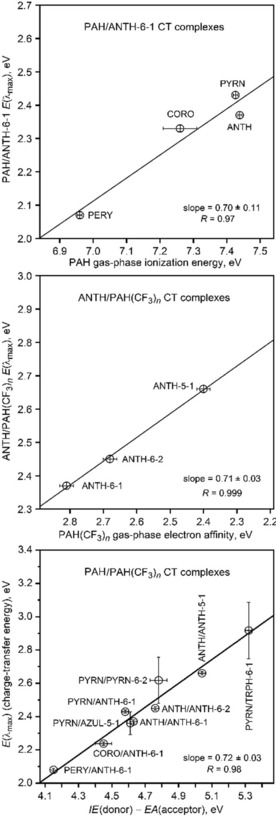
Plots of the solution *E*(*λ*_max_) values for PAH/PAH(CF_3_)_*n*_ CT complexes vs. PAH donor *IE* (top), PAH(CF_3_)_*n*_ acceptor *EA* (middle), and *IE*(donor)—*EA*(acceptor) (bottom; abbreviated Δ(*IE*/*EA*) in Table 1 and elsewhere). The uncertainties for *E*(*λ*_max_) values are equivalent to ±2 nm in *λ*_max_ values except for PYRN/AZUL‐5‐1(±15 nm), PYRN/PYRN‐6‐2(±25 nm), and PYRN/TRPH‐6‐1(±25 nm). The uncertainties for *IE*s and *EA*s are listed in Table 1. Note that the lengths and ranges of values on both axes in each plot are equal (i.e., these are square plots), to show visually and unambiguously that the slopes of the linear least‐squares line fits to the data in each plot are significantly less than unity.

When actual *E*(*λ*_max_) versus *IE* data from the literature are plotted, especially with the most recent and most precise *IE*s, the slopes of the linear regressions are all significantly less than 1.0 (e.g., 0.64,^117^ 0.79,^131^ 0.80,^132^ 0.82,^122^ 0.86,^133^ and 0.87 eV eV^−1[131]^). Figure S29 shows two *E*(*λ*_max_) versus *IE* plots for CT complexes of TCPA with PAHs and other aromatic hydrocarbons. Both plots were prepared for this work using data reported by Chowdhury and Basu in 1960.^117^ In that work, the authors used pre‐1960 LCAO MO energies for the donors in place of ionization energies. They claimed that the slope of their plot (which was not shown in their paper) was 1.0 (“unit slope”), but in fact the plot on the left in Figure S29 shows that the slope is actually 0.86±0.17 eV eV^−1^. When their *E*(*λ*_max_) values are used with the most recent adiabatic *IE*s for the donors, the plot on the right in Figure S29 shows that the slope is 0.64±0.13 eV eV^−1^. As another example, when *E*(*λ*_max_) versus *IE* data for PAH complexes with 1,2,4,5‐C_6_H_2_(CN)_4_ (pyromellitonitrile, hereinafter TCNB) are plotted using modern *IE*s and the CT *E*(*λ*_max_) values reported by Foster and Thompson in 1963,^132^ the slope of the plot is 0.80±0.04 eV eV^−1^, as shown in Figure S30. As a third example, when *E*(*λ*_max_) versus *IE* data for PAH complexes of chloranil are plotted using modern *IE*s and the CT *E*(*λ*_max_) values reported by Briegleb and Czekalla in 1960,^133^ the slope of the plot is 0.86±0.10 eV eV^−1^, as also shown in Figure S30. Two additional plots are shown in Figure S30, with slopes of 0.87±0.10 and 0.80±0.05 eV eV^−1^.^131^ In the present work, we determined *E*(*λ*_max_) for CT complexes of ANTH, CORO, PERY, and PYRN with the electron acceptor ANTH‐6‐1. The plot of *E*(*λ*_max_) versus PAH *IE*, shown at the top of Figure 12, has a slope of 0.70±0.11 eV eV^−1^. In summary, these plots show that *E*(*λ*_max_) values for CT complexes with the same acceptor are proportional to the *IE* of the donor, but the differences in *E*(*λ*_max_) values are attenuated by a multiplicative factor relative to the differences in *IE*s. The multiplicative factor ranges from 0.64 to 0.87, depending on the system (i.e., the attenuation ranges from 13 to 36 %).

There are fewer plots of *E*(*λ*_max_) versus acceptor *EA* for CT complexes with a single donor and a set of structurally‐similar electron acceptors, because there are fewer reliable *EA*s of strong acceptors commonly used for CT complexes. One such correlation was published by Farragher and Page in 1967 for PYRN CT complexes using magnetron electron affinities (the solvent that they used to measure the *λ*_max_ values was not specified).^125^ When we plotted their *E*(*λ*_max_) values versus the present day *EA*s of five of the acceptors that they used, the linear fit had a slope of 0.80±0.03 eV eV^−1^ and an *R* value of 0.998, as shown in Figure S31. In the present work, we determined *E*(*λ*_max_) for CT complexes of ANTH with the three acceptors ANTH‐5‐1, ANTH‐6‐1, and ANTH‐6‐2. The plot of *E*(*λ*_max_) versus ANTH(CF_3_)_5,6_ *EA*, shown in the middle part of Figure 12, has a slope of 0.71±0.03 eV eV^−1^. As with the more numerous *E*(*λ*_max_) versus *IE*, correlations, differences in gas‐phase electron‐acceptor abilities are attenuated, by 20–30 %, when measured in solution.

It is probably the case that differences in gas‐phase electron‐acceptor abilities are similarly attenuated when measured in the solid state. Two studies, by Akutagawa, Nakamura, et al. in 2003^134^ and by Yoshida et al. in 2013,^135^ reported solid‐state *E*(*λ*_max_) values for CT complexes of a common donor with a set of tetracyanoquinodimethane (TCNQ) derivatives. The *EA*s of the TCNQ derivatives were not known (except for TCNQ itself, 3.383(1) eV^136^), so the solid‐state *E*(*λ*_max_) values could only be compared with solution *E*_1/2_(0/−) values. A plot of *E*(*λ*_max_) for 20‐layer Langmuir–Blodgett films of monopyrrolo‐TTF CT complexes with the TCNQ acceptors versus CH_2_Cl_2_ *E*_1/2_(0/−) values, made for this work from the data in the Akutagawa, Nakamura, et al. paper,^134^ and shown in Figure S32, has a slope of 1.06±0.06 eV V^−1^ (only CT complexes with negligible ground‐state electron transfer were used to make this plot). A plot of *E*(*λ*_max_) for KBr pellets of crystalline CORO CT complexes with a set of TCNQ acceptors versus CH_3_CN *E*_1/2_(0/−) values, made for this work from the data in the Yoshida et al. paper^135^ and also shown in Figure S32, has a slope of 0.98±0.05 eV V^−1^. These plots show that the attenuation of gas‐phase *EA*s for these TCNQ acceptors is the same whether it is measured in solution (*E*_1/2_(0/−)) or in the solid state (*E*(*λ*_max_)). The 1:1 correlations were not obvious in these two papers because the *E*_1/2_(0/−) values were plotted against the energy of the charge‐transfer band in cm^−1^, not against the energy of the charge‐transfer band in eV.

When the top and middle plots of Figure 12 are combined along with the *E*(*λ*_max_) values for PYRN/AZUL‐5‐1, PYRN/PYRN‐6‐2, and PYRN/TRPH‐6‐1, the result is a plot of *E*(*λ*_max_) versus Δ(*IE*/*EA*), which is shown at the bottom of Figure 12. The ranges of *IE*s and *EA*s are 0.48 and 0.75 eV, respectively, and the range of Δ(*IE*/*EA*) values is more than 1.1 eV. The slope of this essentially linear plot is 0.70±0.08 eV eV^−1^, showing that the solution phase attenuation is approximately the same for all nine PAH/PAH(CF_3_)_*n*_ CT complexes studied in this work. This is true even though the donors vary in size by 71 %, from ANTH, with 14 C(sp^2^) atoms, to CORO, with 24 C(sp^2^) atoms, and the acceptors vary in size by 33 %, from AZUL‐5‐1, with 30 non‐hydrogen atoms, to PYRN‐6‐2 and TRPH‐6‐1, with 40 and 42 non‐hydrogen atoms, respectively. In addition, the formula unit volume (FUV) of crystalline PYRN/PYRN‐6‐2 (761.8 Å^3^) is 22 % larger than the FUV of crystalline PYRN/AZUL‐5‐1 (622.7 Å^3^).^46^ As far as we are aware, the bottom plot in Figure 12 is unprecedented for CT complexes with structurally‐related donors and acceptors for which experimental *IE*s and *EA*s are known.

#### PAH(CF_3_)_n_ versus TCNB, TCNE, TCNQ, and chloranil as CT complex acceptors

In general, the nine CT complexes we studied in this work do not appear to behave differently in solution relative to the many CT complexes with PAH donors and strong electron acceptors based on planar delocalized π‐systems studied since the 1960s. However, there is an important, consistent, and puzzling difference. As a class of CT complexes, the PAH/PAH(CF_3_)_*n*_ *E*(*λ*_max_) values are all significantly higher than one would expect if one compares them to CT complexes of the same donor with acceptors having comparable *EA*s. For example, the *EA*s of ANTH‐5‐1 and TCNB are 2.40(2)^26^ and 2.2(2) eV,^137^ respectively (the latter experimental value, with a relatively large uncertainty, was recently calculated and predicted to be 2.19 eV^138^). The difference between the *E*(*λ*_max_) values for ANTH/ANTH‐5‐1 and ANTH/TCNB is 0.22 eV, but it is the ANTH‐5‐1 complex that has the higher CT energy: 2.66 eV (466 nm) for ANTH/ANTH‐5‐1 in 1,2‐C_2_H_4_Cl_2_ versus 2.44 eV (508 nm) for ANTH/TCNB in CHCl_3_.^131^ According to Table S2, the difference in solvent could conceivably result in a *λ*_max_ value of 501 nm (2.47 eV) for ANTH/TCNB in CH_2_Cl_2_ or 1,2‐C_2_H_4_Cl_2_, but even in that case the ca. 0.4 eV discrepancy is essentially the same: *E*(*λ*_max_) for ANTH/ANTH‐5‐1 is not lower than *E*(*λ*_max_) for ANTH/TCNB by 0.2 eV, it is higher than *E*(*λ*_max_) for ANTH/TCNB by 0.2 eV. Another example is a comparison of PYRN/TCNB (*λ*_max_=497^131^ or 500 nm^132^ in CHCl_3_ (2.49±0.1 eV)) versus PYRN/ANTH‐6‐1 (*λ*_max_=510 nm in 1,2‐C_2_H_4_Cl_2_ (2.43 eV)). The *λ*_max_ values, even after adjusting for CHCl_3_ versus 1,2‐C_2_H_4_Cl_2_, indicate that ANTH‐6‐1 is the stronger acceptor in solution by ca. 0.1 eV, but their *EA*s show that ANTH‐6‐1 is the stronger acceptor in the gas phase by 0.6±0.2 eV, a discrepancy of 0.5 eV. As a third example, the *EA*s of ANTH‐6‐1 and chloranil are the same to within the uncertainties of the measurements, 2.81(2)^26^ and 2.78(6) eV,^42^ respectively. However, the *E*(*λ*_max_) value for the PYRN/ANTH‐6‐1 CT complex in 1,2‐C_2_H_4_Cl_2_ is 0.40 eV higher than the value for the PYRN/chloranil CT complex in CH_2_Cl_2_ (2.43 eV (510 nm) for PYRN/ANTH‐6‐1 versus 2.03 eV (610 nm) for PYRN/chloranil^94^). A solid‐state manifestation of this discrepancy is that PYRN/(ANTH‐6‐1)_2_ crystals are red,^47^ and PYRN/chloranil crystals are dark green.^139^

A fourth example is the difference in *λ*_max_ values for PERY/ANTH‐6‐1 (1,2‐C_2_H_4_Cl_2_) and PERY/TCNE (CH_2_Cl_2_),^121^ which are 600 and 900 nm, respectively (2.07 and 1.38 eV, respectively). The difference in ANTH‐6‐1 and TCNE *EA*s is 0.35 eV (see Table S6), but the difference in *E*(*λ*_max_) values is 0.69 eV, nearly twice as large. In addition, the difference in *E*(*λ*_max_) values for ANTH/ANTH‐6‐1 (1,2‐C_2_H_4_Cl_2_) and ANTH/TCNE (CH_2_Cl_2_) is 0.64 eV (*λ*_max_ and *E*(*λ*_max_) for ANTH/TCNE in CH_2_Cl_2_ are 717 nm and 1.73 eV, respectively^101^), consistent with the large discrepancy for the PERY CT complexes. As a final example, the *E*(*λ*_max_) value for ANTH/TCNQ in CH_2_Cl_2_ is 1.64 eV (757 nm; the spectrum, recorded for this work, is shown in Figure S33). The difference in ANTH‐6‐1 and TCNQ *EA*s is 0.57 eV (see Table S7), but the difference in *E*(*λ*_max_) values is 0.73 eV. The expectation based on the linear fit in the middle plot in Figure 12 is that the difference in *E*(*λ*_max_) values would be ca. 30 % lower than the difference in *EA*s, not 30 % higher. These six examples consistently show that PAH(CF_3_)_*n*_ acceptors are weaker electron acceptors in CT complexes in solution than the common aromatic CT complex acceptors TCNB, TCNE, TCNQ, and chloranil, even after accounting for differences in gas‐phase *EA*s and even after adjusting for the ca. 30 % attenuation of PAH(CF_3_)_*n*_ acceptor ability in solution demonstrated by the plots in Figure 12. Possible reasons for these significant differences will be considered after the following discussion of the nine (PAH)_*x*_/(PAH(CF_3_)_*n*_)_*y*_ structures.

### (PAH)_*x*_/(PAH(CF_3_)_*n*_)_*y*_ CT complexes in the solid state

#### General comments

Geometric parameters for D/A co‐crystal structures with stacks of alternating parallel donors and acceptors can be defined in a number of ways, sometimes differing mathematically and sometimes differing only in terminology. The parameters listed in Table 4 were chosen to facilitate the discussion below. The most important structural parameters are those that describe the nearly‐parallel donor/acceptor pair with the greatest amount of overlap of their respective π‐systems. These are 1) the dihedral angle between the least‐squares planes of the C(sp^2^) atoms of the donor and acceptor (D/A LSP dihedral ∡), 2) the rotation of the donor and acceptor major axes when viewed as a parallel projection with the donor LSP in the plane of the page, 3) the number of acceptor C(sp^2^) atoms that overlap the π‐system of the donor, and 4) the perpendicular out‐of‐plane (OOP) displacements of those acceptor C(sp^2^) atoms from the LSP of the donor (A→D(LSP) OOPs). The first three of these are evident in the drawings in Figure 10 and Figures S21–S25. The fourth is listed in Table 4 as the range of A→D(LSP) OOPs and as the average A→D(LSP) OOP.

A fifth parameter, the D/A interplanar separation, can be defined as half the perpendicular distance between the LSPs of the PAH donors on either side of a PAH(CF_3_)_*n*_ acceptor or, in the structures of ANTH/(ANTH‐5‐1)_2_ and PYRN/(TRPH‐6‐1)_2_, half the perpendicular distance between the LSPs of the acceptors on either side of the donor. However, the fourth and fifth parameters are virtually the same for the (PAH)_*x*_/(PAH(CF_3_)_*n*_)_*y*_ structures: they differ by only 0.01 or 0.02 Å for five of the structures and by no more than 0.07 Å for any of the structures, as shown by the values listed in Table S8. In our opinion, the average A→D(LSP) OOP is a more relevant measure of the D/A interplanar separation because it is based on the region of π–π overlap and avoids the ambiguity due to a non‐zero D/A LSP dihedral ∡.

#### Stoichiometry, stacking, packing, and bending

The CT complexes ANTH/ANTH‐5‐1 and PYRN/ANTH‐6‐1 were found to have 1/1 stoichiometry in solution but formed 1/2 co‐crystals. In contrast, the solution and solid‐state stoichiometries were 1/1 for ANTH/ANTH‐6‐1 and PERY/ANTH‐6‐1. However, it is possible that different crystalline phases of (PAH)_*x*_/(PAH(CF_3_)_*n*_)_*y*_ with different *x*/*y* stoichiometries could exist if the crystals were grown under different conditions. This is a known phenomenon for some D/A co‐crystal combinations. For example, the crystallization of CORO with 7,7,8,8‐tetrafluoro(tetracyanoquinodimethane) (F_4_TCNQ) from CH_2_Cl_2_/pentane produced 1/1 CT co‐crystals, CORO/F_4_TCNQ,^67^ whereas co‐sublimation produced 2/1 CT co‐crystals, (CORO)_2_/F_4_TCNQ.^68^, ^73^

Eight of the nine (PAH)_*x*_/(PAH(CF_3_)_*n*_)_*y*_ structures consist of hexagonal arrays of stacks of nearly parallel PAH donors and PAH(CF_3_)_*n*_ acceptors with significant π–π overlap and average A→D(LSP) OOPs that span a narrow range, from 3.49 in ANTH/(ANTH‐5‐1)_2_ to 3.61 Å in PYRN/AZUL‐5‐1 and (CORO)_2_/ANTH‐6‐1. Two of them, ANTH/(ANTH‐5‐1)_2_ and PYRN/(TRPH‐6‐1)_2_, contain pairs of acceptors with π–π overlap. This was not surprising because the individual structures of ANTH‐5‐1 and TRPH‐6‐1 contain stacks of molecules with π–π overlap similar to that for the acceptor pairs in ANTH/(ANTH‐5‐1)_2_ and PYRN/(TRPH‐6‐1)_2_ (see Figures S21 and S25).^26^ Many structural investigations have shown that partial fluorination, trifluoromethylation, and or *n*‐perfluoroalkylation tend to overcome herringbone crystal packing in PAH derivatives.^27^, ^28^, ^37^, ^38^, ^39^, ^60^, ^140^, ^141^, ^142^ Nevertheless, the structure of PYRN/(ANTH‐6‐1)_2_ does not consist of stacks. Instead, discrete ANTH‐6‐1/PYRN/ANTH‐6‐1 ({A/D/A}) complexes are packed in herringbone sandwich layers in crystallographic *ac*‐planes, such that the PYRN centroids are rigorously co‐planar. The {A/D/A} complexes in each layer form a pseudo‐square net depicted with dashed lines in Figure S34. The relative areas of π–π overlap for the (PAH)_*x*_/(PAH(CF_3_)_*n*_)_*y*_ complexes are listed in Table 5.

**Table 5:** Relative Areas of π–π Overlap for D_*x*_/A_*y*_ Co‐crystals.

structure	relative area of π–π overlap^[a]^	number of hexagons in donor	number of hexagons in acceptor
ANTH/(ANTH‐5‐1)_2_	1.00	3	3
ANTH/ANTH‐6‐1	1.02	3	3
ANTH/ANTH‐6‐2	1.02	3	3
(CORO)_2_/ANTH‐6‐1	1.24	7	3
PERY/ANTH‐6‐1	1.03	5	3
PYRN/(ANTH‐6‐1)_2_	1.12	4	3
PYRN/AZUL‐5‐1	1.10	4	2^[b]^
PYRN/PYRN‐6‐2	1.57, 1.62	4	4
PYRN/(TRPH‐6‐1)_2_	1.26	4	4

[a] The relative areas were measured by printing each complex at the same scale in parallel perspective, and cutting out and weighing the area of π–π overlap. [b] One pentagon and one heptagon, not two hexagons.

Figure S25 shows the π–π overlap for the four co‐crystal structures with PYRN as the donor. The area of overlap depends on the size and shape of the acceptor π‐system. The acceptors AZUL‐5‐1 and ANTH‐6‐1 have 10 and 14 C(sp^2^) atoms, respectively. However, the areas of π‐π overlap are virtually identical in PYRN/AZUL‐5‐1^46^ and PYRN/(ANTH‐6‐1)_2_.^47^ The acceptors PYRN‐6‐2 and TRPH‐6‐1, have 16 and 18 C(sp^2^) atoms, respectively, and both have four aromatic hexagons. However, the area of π–π overlap in PYRN/PYRN‐6‐2 is 26 % larger than in PYRN/(TRPH‐6‐1)_2_ and 43 % larger than in PYRN/AZUL‐5‐1 or PYRN/ANTH‐6‐1 because of the better match of the donor and acceptor shapes in PYRN/PYRN‐6‐2. Nevertheless the greater π–π overlap does not lead to a lower‐than‐expected CT *E*(*λ*_max_) for PYRN/PYRN‐6‐2 relative to the other PYRN/PAH(CF_3_)_*n*_ CT complexes. The *E*(*λ*_max_) versus Δ(*IE*/*EA*) plot in Figure 12 shows that, if anything, the point for PYRN/PYRN‐6‐2 is not displaced from the linear regression line to a lower *E*(*λ*_max_) value (in fact, it may be displaced to a higher *E*(*λ*_max_) value). The acceptor *EA*, not the area of D/A π–π overlap, is proportional to *E*(*λ*_max_). A plot of *E*(*λ*_max_) versus Δ(*IE*/*EA*) for just the four PYRN/PAH(CF_3_)_*n*_ CT complexes is shown in Figure S35. The slope of that plot, 0.73±0.09 eV eV^−1^, is the same as the slope for the plot in Figure 12 for all nine PAH/PAH(CF_3_)_*n*_ CT complexes, 0.72±0.03 eV eV^−1^, and the point for PYRN/PYRN‐6‐2 is slightly above the line in that plot as well.

Figure S36 shows the four co‐crystal structures with PYRN as the donor, oriented so that the PYRN aromatic core is perpendicular to the plane of the page. The planarity of the aromatic core in AZUL‐5‐1 and the nonplanarity of the aromatic cores in PYRN‐6‐2 and TRPH‐6‐1 are readily apparent. The structure of the ANTH‐6‐1 acceptor in PYRN/(ANTH‐6‐1)_2_ is nonplanar in a particularly distinctive way. The two halves of the ANTH core have been bent away from planarity by 13.4° at the ANTH C9⋅⋅⋅C10 hinge (the bend angle, *θ*, is defined in Figure S37).^47^ Significantly, the CF_3_ groups on C9 and C10 are eclipsed.

We recently reported that ANTH derivatives with CF_3_ or *n*‐R_F_ groups on C9 and C10, including ANTH‐6‐1, are more stable when they are bent in this way and have eclipsed central CF_3_ groups, than when they are planar with staggered central CF_3_ groups. The DFT optimized structure of ANTH‐6‐1 is bent by 17.5° and is 5.7 kJ mol^−1^ more stable than the DFT structure with a planar core.^47^ (Similarly, the DFT structure of 6,13‐pentacene(CF_3_)_2_ with eclipsed CF_3_ groups and *θ*=19.1° was found to be 9.0 kJ mol^−1^ more stable than the structure with staggered CF_3_ groups and *θ*=0.0°.^63^) The crystal structure of ANTH‐6‐1 is also bent, with *θ*=7.4° and partially eclipsed central CF_3_ groups. In contrast, the ANTH‐6‐1 molecule in the structures of ANTH/ANTH‐6‐1, (CORO)_2_/ANTH‐6‐1, and PERY/ANTH‐6‐1 have planar cores (*θ*=0.0°) and staggered CF_3_ groups, as shown in Figure S37.

#### Comparisons with DFT PAH/PAH(CF_3_)_n_ structures

DFT optimization of a single D/A complex in each of the nine X‐ray structures afforded the opportunity to observe the inherent structures of the complexes without the influence of neighbouring molecules. In particular, we were interested in comparing the planar versus bent conformation of ANTH‐6‐1 in the four D/A complexes PYRN/ANTH‐6‐1, ANTH/ANTH‐6‐1, CORO/ANTH‐6‐1, and PYRN/ANTH‐6‐1. These results are shown in Figures S38‐S41. The ANTH‐6‐1 molecule is bent, with eclipsed or partially eclipsed central CF_3_ groups, in all four DFT optimized complexes, with θ angles of 15.1° in PYRN/ANTH‐6‐1, 10.9° in ANTH/ANTH‐6‐1, 11.4° in CORO/ANTH‐6‐1, and 9.0° in PERY/ANTH‐6‐1. Since the lowest‐energy DFT structure of an isolated ANTH‐6‐1 molecule has *θ*=17.5°, interactions with the donors ANTH, CORO, and PERY reduce the bend to 9.0–11.4°. The other relevant structural parameters for the four DFT optimized ANTH‐6‐1 complexes, including the LSP tilt angles, π–π OOPs, and rotation of the D/A major axes, are essentially the same as in the crystal structures. The reason that *θ*=0.0° for the ANTH‐6‐1 molecules in the crystal structures of ANTH/ANTH‐6‐1, (CORO)_2_/ANTH‐6‐1, and PERY/ANTH‐6‐1 is presumably because the ANTH‐6‐1 acceptor in those three structures has a nearly parallel donor molecule on either side.

The other DFT optimized D/A structures are also similar to the crystal structures as far as LSP tilt angles, π–π A→D(LSP) OOPs, and rotations of the D/A major axes are concerned. However, the bend angle of the acceptor ANTH‐5‐1, which has only one, not two, central CF_3_ groups, is essentially the same in the crystal structure of ANTH/(ANTH‐5‐1)_2_ (5.0°), in DFT optimized ANTH/ANTH‐5‐1 (5.3°), and in the crystal structure of ANTH‐5‐1 (4.7 and 5.7° for two independent molecules).^26^ The structures of the DFT optimized and crystal structures of ANTH/ANTH‐5‐1 are compared in Figure S42.

#### Comparisons with literature co‐crystal structures of PAHs and common strong acceptors

With one important difference, the D/A complexes in the crystal structures of ANTH/(ANTH‐5‐1)_2_ and ANTH/NAPH(F)_8_ 44 share many similarities, as shown in Figure S43 (see also Table 4). Both structures consist of hexagonal arrays of stacks of nearly‐parallel donors and acceptors. (In spite of the different donor/acceptor stoichiometries, the ANTH donors in both structures have acceptors on both sides in the stacks.) The rotations of the major axes of ANTH donors and the acceptors with respect to one another, listed in Table 4, are 17.1 and 19.9°, respectively, in ANTH/(ANTH‐5‐1)_2_ and ANTH/NAPH(F)_8_. The D/A LSP dihedral angles are 2.2 and 2.7°, respectively, and the major and minor axis angles to the ANTH⋅⋅⋅ANTH (i.e., D⋅⋅⋅D) centroid vector are identical in the two structures, 14.8 and 7.6°, respectively. Although the smaller π‐system of NAPH(F)_8_ results in a smaller region of π–π overlap in ANTH/NAPH(F)_8_, the numbers of acceptor C(sp^2^) atoms situated within the boundary of the donor π‐system in the parallel projections shown in Figure S43 only differ by one atom (7 in ANTH/(ANTH‐5‐1)_2_ and 6 in ANTH/NAPH(F)_8_). The one important difference is that the perpendicular D/A interplanar separations are 3.49 and 3.35 Å, respectively. The separation of the nearly parallel donor and acceptor π‐systems in ANTH/NAPH(F)_8_ is graphite‐like, while the separation in ANTH/(ANTH‐5‐1)_2_ is 0.14 Å larger. The difference cannot be due to a ground‐state charge‐transfer electrostatic attraction in ANTH/NAPH(F)_8_, because NAPH(F)_8_ does not form charge‐transfer complexes with planar aromatic compounds, including tetrathiafulvalene^143^ (presumably because the estimated *EA* of NAPH(F)_8_ is only ca. 1 eV^29^).

The graphite‐like D/A separation in ANTH/NAPH(F)_8_ is commonly observed in D/A co‐crystals involving PAH donors and aromatic acceptors with electron‐withdrawing groups, such as (see Table S6 for abbreviations) ANTH/TCNB (3.37 Å),^75^ CORO/(MeO)_2_TCNQ (3.37 Å),^67^ 9‐ANTH(Me)/TCNB (3.34 Å),^71^ ANTH/NBDF (3.32 Å),^74^ PERY/Br_4_BQ (3.31 Å),^144^ CORO/TCNQ (3.26 Å),^67^ PYRN/F_4_BQ (3.24 Å),^144^ and ANTH/F_4_ANTH(O)_2_ (3.38 Å).^145^ Some related structures have intermediate D/A interplanar separations (i.e., 3.4–3.5 Å), including TETR/TCNQ (3.44 Å)^146^ and PYRN/chloranil (3.47 Å).^139^ These structures are shown in Figures S44 and S45.

The average A→D(LSP) π–π OOP displacement distance for PYRN/ANTH‐6‐1 is 3.55 Å, with 10 overlapped C(sp^2^) atoms. The average A→D(LSP) OOP distance for PYRN/chloranil, at 3.47 Å, is shorter by ca. 0.1 Å. Returning to a question posed in an earlier section, why is *E*(*λ*_max_) 0.4 eV smaller for PYRN/chloranil if ANTH‐6‐1 and chloranil have the same *EA*? In an effort to gauge whether a difference of 0.1 or 0.2 Å in π–π interplanar separation could have an appreciable effect on *E*(*λ*_max_), we used DFT to predict Δ*E*_CT_ for PYRN/chloranil at various separations starting with the crystal structure coordinates. A plot of the results of these calculations is shown in Figure S46. Increasing the chloranil→PYRN π–π separation by 0.1 and 0.2 Å only increased Δ*E*_CT_ by 0.016 and 0.035 eV, respectively. Therefore, the majority of the 0.4 eV difference in *E*(*λ*_max_) cannot be attributed to the π–π interplanar separation difference. Perhaps the two *E*(*λ*_max_) are so different because much of the negative charge on the ANTH‐6‐1^−^ radical anion in the CT excited state is spread out on the 18 F atoms instead of localized in the C(sp^2^) π‐system? Perhaps the negative charge is not as spread out on the two O and four Cl atoms in the chloranil^−^ radical anion. Another possibility is that different solvation energies attenuate the gas‐phase *EA*s of ANTH‐6‐1 and chloranil to significantly different extents.

The average A→D(LSP) OOP distance in ANTH/(ANTH‐5‐1)_2_ is 3.49 Å, with 7 overlapped C(sp^2^) atoms. The average A→D(LSP) OOP distance for ANTH/TCNB, at 3.37 Å,^75^ is shorter by 0.12 Å, and the average A→D(LSP) OOP distance for the related CT complex 9‐ANTH(Me)/TCNB, at 3.34 Å,^71^ is shorter by 0.15 Å. As discussed above, it is puzzling why *E*(*λ*_max_) is 0.2 eV lower for ANTH/TCNB^131^ than for ANTH/ANTH‐5‐1 even though the gas‐phase *EA* of ANTH‐5‐1 is ca. 0.2 eV higher than the *EA* of TCNB. This *E*(*λ*_max_) anomaly, ca. 0.4 eV, is as large as the anomaly discussed in the previous paragraph. We also used DFT to predict Δ*E*_CT_ for ANTH/TCNB at various separations, starting with the crystal structure coordinates. A plot of the calculated results is also shown in Figure S46. In this case, increasing the TCNB→ANTH π–π separation by 0.1 and 0.2 Å increased Δ*E*_CT_ by 0.041 and 0.075 eV, respectively. Therefore, as in the previous comparison, only 10–15 % of the 0.4 eV *E*(*λ*_max_) anomaly can be attributed to the π–π interplanar separation difference. The majority must be due to factors that remain to be investigated and identified.

#### Comparison of the structure of (CORO)_2_/ANTH‐6‐1 with (CORO)_2_/F_4_TCNQ

The different CORO/CORO π–π overlaps in the crystal structures of (CORO)_2_/ANTH‐6‐1, (CORO)_2_/F_4_TCNQ),^68^, ^73^ and pure CORO at 100 K are shown in Figure S47, and the different molecular stackings and packings in these three structures are shown in Figure S48. The (CORO)_2_ pair in (CORO)_2_/ANTH‐6‐1 exhibits both major (11 %) and minor (21 %) axis slippage and a greater number of π‐overlapped C atoms (15) than the (CORO)_2_ pair in (CORO)_2_/F_4_TCNQ (12), which exhibits almost no major axis slippage and 30 % minor axis slippage, although the areas of overlap are approximately the same. The neighbouring parallel molecules in the structure of pure CORO are on average closer together than in the other two cases (3.40 Å vs. 3.45 Å) but with a much greater minor axis slippage (51 %), fewer C atom overlaps, and a much smaller area of π–π overlap.

## Summary and Conclusions

This is the first study of CT complexes of a set of structurally‐similar aromatic acceptors that 1) have five or six of the same electron‐withdrawing substituent and no other substituents, 2) have precise experimental gas‐phase *EA*s (±0.02 eV) measured with the same instrumentation in the same laboratory (all except PYRN‐6‐2), 3) have precise experimental *E*_1/2_(0/−) values (±0.01 V) measured in the same electrolyte solution with the same instrumentation in the same laboratory, 4) have *E*(*λ*_max_) values determined in the same solvent for CT complexes with a common donor (ANTH with ANTH‐5‐1, ANTH‐6‐1, and ANTH‐6‐2, and PYRN with ANTH‐6‐1, AZUL‐5‐1, PYRN‐6‐2, and TRPH‐6‐1), and 5) have nine X‐ray diffraction co‐crystal structures to analyse.

Our findings include the following:

1. With one exception, the co‐crystal structures share many similarities with other aromatic D/A co‐crystal structures, including the formation of hexagonal arrays of mixed D/A stacks with essentially co‐planar donors and acceptors that have large areas of π–π overlap and either 1/1, 1/2, or 2/1 donor/acceptor stoichiometries. However, the exception is that the D/A interplanar separation in the nine (PAH)_*x*_/(PAH(CF_3_)_*n*_)_*y*_ structures are 3.55±0.06 Å, wider by 0.1–0.2 Å than in most other aromatic D/A co‐crystal structures. This could possibly be due to the steric requirements of the CF_3_ substituents, but a complete understanding of this exception remains to be determined. Our DFT calculations show that increasing a D/A interplanar separation by 0.1 or 0.2 Å should have a relatively small effect on the CT energy (i.e., *E*(*λ*_max_)), on the order of 0.02–0.04 eV or 0.04–0.08 eV, respectively.
2. Four of the nine PAH/PAH(CF_3_)_*n*_ combinations form 1/1 D/A complexes in solution. Two have formation constants of ca. 2 m ^−1^, comparable to the formation constants of other aromatic D/A complexes. However, the *E*(*λ*_max_) values are consistently higher, by as much as 0.4 eV, than expected given the CT *E*(*λ*_max_) values and the acceptor *EA*s for common aromatic D/A complexes. For reasons that are still not clear, the PAH(CF_3_)_*n*_ compounds are weaker electron acceptors in solution than other aromatic acceptors with comparable gas‐phase *EA*s, even after adjusting for the unusual 3.55±0.06 Å PAH/PAH(CF_3_)_*n*_ interplanar separations.
3. A plot of *E*(*λ*_max_) versus Δ(*IE*/*EA*) for the nine PAH/PAH(CF_3_)_*n*_ CT complexes is linear with a slope of 0.72±0.03 eV eV^−1^. This plot is the first of its kind for CT complexes with structurally related donors and acceptors for which precise experimental gas‐phase *IE*s and *EA*s are known. It demonstrates that conclusions based on the common assumption that the slope of a CT *E*(*λ*_max_) versus Δ(*IE*/*EA*) plot is unity may be incorrect in at least some cases and should be reconsidered.

## Conflict of interest

The authors declare no conflict of interest.

## Supporting information

As a service to our authors and readers, this journal provides supporting information supplied by the authors. Such materials are peer reviewed and may be re‐organized for online delivery, but are not copy‐edited or typeset. Technical support issues arising from supporting information (other than missing files) should be addressed to the authors.

Supplementary

## References

[1] J. Zhang , W. Xu , P. Sheng , G. Y. Zhao , D. B. Zhu , Acc. Chem. Res. 2017, 50, 1654–1662. 28608673 10.1021/acs.accounts.7b00124

[2] A. Ueda , Bull. Chem. Soc. Jap. 2017, 90, 1181–1188.

[3] V. Colombo , L. Lo Presti , A. Gavezzotti , CrystEngComm 2017, 19, 2413–2423.

[4] N. R. Goud , A. J. Matzger , Cryst. Growth Des. 2017, 17, 328–336.

[5] L. Zoppi , K. K. Baldridge , Int. J. Quantum Chem. 2018, 118, e25413.

[6] J. Zhang , J. Q. Jin , H. X. Xu , Q. C. Zhang , W. Huang , J. Mater. Chem. C 2018, 6, 3485–3498.

[7] L. J. Sun , W. G. Zhu , F. X. Yang , B. L. Li , X. C. Ren , X. T. Zhang , W. P. Hu , Phys. Chem. Chem. Phys. 2018, 20, 6009–6023. 29238770 10.1039/c7cp07167a

[8] Z. Z. Li , L. S. Liao , X. D. Wang , Small 2018, 14, 1702952.

[9] Y. Wang , W. G. Zhu , H. L. Dong , X. T. Zhang , R. J. Li , W. P. Hu , Top. Curr. Chem. 2016, 374, 83. 10.1007/s41061-016-0081-8 27885589

[10] M. Wolf , A. Herrmann , A. Hirsch , D. M. Guldi , J. Am. Chem. Soc. 2017, 139, 11779–11788. 28749146 10.1021/jacs.7b04589

[11] Z.-F. Yao , J.-Y. Wang , J. Pei , Cryst. Growth Des. 2018, 18, 7–15, and references therein.

[12] H. Jiang , P. Hu , J. Ye , K. K. K. Zhang , Y. Long , W. P. Hu , C. Kloc , J. Mater. Chem. C 2018, 6, 1884–1902.

[13] P. Hu , S. C. Wang , A. Chaturvedi , F. X. Wei , X. T. Zhu , X. T. Zhang , R. J. Li , Y. X. Li , H. Jiang , Y. Long , C. Kloc , Cryst. Growth Des. 2018, 18, 1776–1785.

[14] G. C. Saunders , T. T. Wehr-Candler , J. Fluorine Chem. 2013, 153, 162–164.

[15] H. I. Althagbi , J. R. Lane , G. C. Saunders , S. J. Webb , J. Fluorine Chem. 2014, 166, 88–95.

[16] A. M. Hiszpanski , J. D. Saathoff , L. Shaw , H. Wang , L. Kraya , F. Luettich , M. A. Brady , M. L. Chabinyc , A. Kahn , P. Clancy , Y. L. Loo , Chem. Mater. 2015, 27, 1892–1900.

[17] H. I. Althagbi , D. R. Bernstein , W. C. Crombie , J. R. Lane , D. K. McQuiston , M. A. Oosterwijk , G. C. Saunders , W. Zou , J. Fluorine Chem. 2018, 206, 61–71.

[18] N. Castagnetti , M. Masino , C. Rizzoli , A. Girlando , C. Rovira , Phys. Rev. Mater. 2018, 2, 024602.

[19] B. Milián-Medina , J. Gierschner , J. Phys. Chem. Lett. 2017, 8, 91–101, and references therein. 27958747 10.1021/acs.jpclett.6b02495

[20] M. O. BaniKhaled , J. D. Becker , M. Koppang , H. Sun , Cryst. Growth Des. 2016, 16, 1869–1878, and references therein.

[21] H. Sun , J. H. Kramer , in New Fluorinated Carbons: Fundamentals and Applications (Eds.: O. V. Boltalina, T. Nakajima), Elsevier, Amsterdam, 2017, pp. 155–176.

[22] L. K. San , S. N. Spisak , C. Dubceac , S. H. M. Deng , I. V. Kuvychko , M. A. Petrukhina , X.-B. Wang , A. A. Popov , S. H. Strauss , O. V. Boltalina , Chem. Eur. J. 2018, 24, 1441–1447. 29178382 10.1002/chem.201704868

[23] I. V. Kuvychko , T. T. Clikeman , C. Dubceac , Y.-S. Chen , M. A. Petrukhina , S. H. Strauss , A. A. Popov , O. V. Boltalina , Eur. J. Org. Chem. 2018, 4233–4245.

[24] H. Sun , A. Putta , M. Billion , J. Phys. Chem. A 2012, 116, 8015–8022. 22780095 10.1021/jp301718j

[25] I. V. Kuvychko , S. N. Spisak , Y. S. Chen , A. A. Popov , M. A. Petrukhina , S. H. Strauss , O. V. Boltalina , Angew. Chem. Int. Ed. 2012, 51, 4939–4942; 10.1002/anie.201200178 22492671

[26] I. V. Kuvychko , K. P. Castro , S. H. M. Deng , X.-B. Wang , S. H. Strauss , O. B. Boltalina , Angew. Chem. Int. Ed. 2013, 52, 4871–4874; 10.1002/anie.201300085 23526691

[27] H. Sun , U. K. Tottempudi , J. D. Mottishaw , P. N. Basa , A. Putta , A. G. Sykes , Cryst. Growth Des. 2012, 12, 5655–5662.

[28] J. Schwaben , N. Münster , T. Breuer , M. Klues , K. Harms , G. Witte , U. Koert , Eur. J. Org. Chem. 2013, 1639–1643. 10.1002/chem.201501399 26248605

[29] Y. Xie , H. F. Schaefer III , F. A. Cotton , Chem. Commun. 2003, 102–103. 10.1039/b208831m 12610986

[30] X. Feng , Q. Li , J. Gu , F. A. Cotton , Y. Xie , G. F. Schaefer , J. Phys. Chem. A 2009, 113, 887–894. 19133794 10.1021/jp809110f

[31] Y.-C. Chang , M.-Y. Kuo , C.-P. Chen , H.-F. Lu , I. Chao , J. Phys. Chem. C 2010, 114, 11595–11601.

[32] J. Dhar , U. Salzner , S. Patil , J. Mater. Chem. C 2017, 5, 7404–7430, and references therein.

[33] B. Maiti , A. Schubert , S. Sarkar , S. Bhandari , K. Wang , Z. Li , E. Geva , R. J. Twieg , B. D. Dunietz , Chem. Sci. 2017, 8, 6947–6953. 29147520 10.1039/c7sc02491f PMC5642104

[34] M. Perry , C. Carra , M. N. Chrétien , J. C. Scaiano , J. Phys. Chem. A 2007, 111, 4884–4889. 17516633 10.1021/jp0702797

[35] Y. Sakamoto , T. Suzuki , M. Kobayashi , Y. Gao , Y. Fukai , Y. Inoue , F. Sato , S. Tokito , J. Am. Chem. Soc. 2004, 126, 8138–8140. 15225054 10.1021/ja0476258

[36] C. P. Brock , J. D. Dunitz , Acta Crystallogr. Sect. B 1990, 46, 795–806.

[37] F. Cozzi , S. Bacchi , G. Filippini , T. Pilati , A. Gavezzotti , Chem. Eur. J. 2007, 13, 7177–7184. 17568459 10.1002/chem.200700267

[38] I. Y. Bagryanskaya , Y. V. Gatilov , A. M. Maksimov , V. E. Platonov , A. V. Zibarev , J. Fluorine Chem. 2005, 126, 1281–1287.

[39] S. Bhandary , D. Chopra , Cryst. Growth Des. 2018, 18, 3027–3036.

[40] C. Hansch , A. Leo , R. W. Taft , Chem. Rev. 1991, 91, 165–195.

[41] T. Siodła , W. P. Ozimiński , M. Hoffmann , H. Koroniak , T. M. Krygowski , J. Org. Chem. 2014, 79, 7321–7331. 25046196 10.1021/jo501013p

[42] T. Heinis , S. L. Chowdhury , S. Scott , P. Kebarle , J. Am. Chem. Soc. 1988, 110, 400–407.

[43] Y. Matsubara , A. Kimura , Y. Yamaguchi , Z. Yoshida , Org. Lett. 2008, 10, 5541–5544. 19007178 10.1021/ol802337v

[44] J. C. Collings , K. P. Roscoe , R. L. Thomas , A. S. Batsanov , L. M. Stimson , J. A. K. Howard , T. B. Marder , New J. Chem. 2001, 25, 1410–1417.

[45] J. C. Collings , A. S. Batsanov , J. A. K. Howard , T. B. Marder , Can. J. Chem. 2006, 84, 238–242.

[46] T. T. Clikeman , E. V. Bukovsky , I. V. Kuvychko , L. K. San , S. H. M. Deng , X.-B. Wang , Y.-S. Chen , S. H. Strauss , O. B. Boltalina , Chem. Commun. 2014, 50, 6263–6266. 10.1039/c4cc00510d 24788399

[47] N. J. DeWeerd , E. V. Bukovsky , K. P. Castro , I. V. Kuvychko , A. A. Popov , S. H. Strauss , O. V. Boltalina , J. Fluorine Chem. 2019, 221, 1–7.

[48] C. Dubceac , Y. Sevryugina , I. V. Kuvychko , O. V. Boltalina , S. H. Strauss , M. A. Petrukhina , Cryst. Growth Des. 2018, 18, 307–311.

[49] L. K. San , E. V. Bukovsky , I. V. Kuvychko , A. A. Popov , S. H. Strauss , O. V. Boltalina , Chem. Eur. J. 2014, 20, 4373–4379. 24591166 10.1002/chem.201304554

[50] K. P. Castro, “Trifluoromethylated Fullerenes and Polycyclic Aromatic Hydrocarbons and Anaerobically Milled Silicon Nanoparticles,” Ph.D. Dissertation thesis, Colorado State University **2015**.

[51] K. P. Castro , T. T. Clikeman , N. J. DeWeerd , E. V. Bukovsky , K. C. Rippy , I. V. Kuvychko , G. L. Hou , Y.-S. Chen , X.-B. Wang , S. H. Strauss , O. V. Boltalina , Chem. Eur. J. 2016, 22, 3930–3936. 26617289 10.1002/chem.201504122

[52] C. G. Krespan , B. C. McKusick , T. L. Cairns , J. Am. Chem. Soc. 1961, 83, 3428–3432.

[53] Y. Kobayashi , I. Kumadaki , S. Sato , N. Hara , E. Chikami , Chem. Pharm. Bull. 1970, 18, 2334–2339.

[54] K. Hosokawa , K. Inukai , Nippon Kagaku Kaishi 1972, 383–386.

[55] M. Mintas , H. Gusten , P. G. Williard , J. Photochem. Photobiol. A 1989, 48, 341–344.

[56] S. Toyota , Y. Watanabe , H. Yoshida , M. Ōki , Bull. Chem. Soc. Japan 1995, 68, 2751–2756.

[57] S. Furuta , M. Kuroboshi , T. Hiyama , Bull. Chem. Soc. Japan 1999, 72, 805–819.

[58] B. M. Schmidt , S. Seki , B. Topolinski , K. Ohkubo , S. Fukuzumi , H. Sakurai , D. Lentz , Angew. Chem. Int. Ed. 2012, 51, 11385–11388; 10.1002/anie.201205757 23047849

[59] B. M. Schmidt , B. Topolinski , M. Yamada , S. Higashibayashi , M. Shionoya , H. Sakurai , D. Lentz , Chem. Eur. J. 2013, 19, 13872–13880. 24009101 10.1002/chem.201301910

[60] S. Yamada , K. Kinoshita , S. Iwama , T. Yamazaki , T. Kubota , T. Yajima , RSC Adv. 2013, 3, 6803–6806.

[61] S. Yamada , S. Iwama , K. Kinoshita , T. Yamazaki , T. Kubota , T. Yajima , Tetrahedron 2014, 70, 6749–6756.

[62] B. M. Schmidt , A. K. Meyer , D. Lentz , CrystEngComm 2017, 19, 1328–1333.

[63] S. Yamada , K. Kinoshita , S. Iwama , T. Yamazaki , T. Kubota , T. Yajima , K. Yamamoto , S. Tahara , Org. Biomol. Chem. 2017, 15, 2522–2535. 28256673 10.1039/c7ob00043j

[64] NIST Webbook (http://webbook.nist.gov/chemistry).

[65] E. S. Pysh , N. C. Yang , J. Am. Chem. Soc. 1963, 85, 2124–2130.

[66] M. Dietrich , J. Heinze , J. Am. Chem. Soc. 1990, 112, 5142–5145.

[67] Y. Yoshida , Y. Kumagai , M. Mizuno , G. Saito , Cryst. Growth Des. 2015, 15, 1389–1394.

[68] Y. Yoshida , Y. Kumagai , M. Mizuno , K. Isomura , Y. Nakamura , H. Kishida , G. Saito , Cryst. Growth Des. 2015, 15, 5513–5518.

[69] A. S. Batsanov , J. C. Collings , J. A. K. Howard , T. B. Marder , Acta Crystallogr. Sect. E 2001, 57, o950–o952. 10.1107/s0108270101007557 11443271

[70] J. C. Collings , K. P. Roscoe , E. G. Robins , A. S. Batsanov , L. M. Stimson , J. A. K. Howard , S. J. Clark , T. B. Marder , New J. Chem. 2002, 26, 1740–1746.

[71] R. O. Al-Kaysi , A. M. Müller , R. J. Frisbee , C. J. Bardeen , Cryst. Growth Des. 2009, 9, 1780–1785.

[72] T. Salzillo , M. Masino , G. Kociok-Kohn , D. Di Nuzzo , E. Venuti , R. G. Della Valle , D. Vanossi , C. Fontanesi , A. Girlando , A. Brillante , E. Da Como , Cryst. Growth Des. 2016, 16, 3028–3036.

[73] O. Kataeva , M. Khrizanforov , Y. Budnikova , D. Islamov , T. Burganov , A. Vandyukov , K. Lyssenko , B. Mahns , M. Nohr , S. Hampel , M. Knupfer , Cryst. Growth Des. 2016, 16, 331–338.

[74] G. Berionni , P. A. Bertelle , J. Marrot , R. Goumont , J. Am. Chem. Soc. 2009, 131, 18224–18225. 19968279 10.1021/ja908747j

[75] J. Lefebvre , G. Odou , M. Muller , A. Mierzejewski , T. Luty , Acta Crystallogr. Sect. B 1989, 45, 323–336.

[76] X.-B. Wang , L.-S. Wang , Rev. Sci. Instrum. 2008, 79, 0731108. 10.1063/1.2957610 18681692

[77] G. M. Sheldrick, SADABS: A program for area detector absorption corrections, **2004**.

[78] G. M. Sheldrick, Crystallography Program APEX2 v. 5-0, Bruker AXS, Madison, WI, **2014**.

[79] G. M. Sheldrick, SHELXTL, Bruker AXS, Madison, WI, **2001**.

[80] O. V. Dolomanov , L. J. Bourhis , R. J. Gildea , J. A. K. Howard , H. Puschmann , J. Appl. Crystallogr. 2009, 42, 339–341. 10.1107/S0021889811041161 PMC3236671 22199401

[81] A. D. Becke , J. Chem. Phys. 1993, 98, 5648–5652.

[82] S. Grimme , WIREs Comput. Mol. Sci. 2011, 1, 211–228.

[83] F. Weigend , R. Ahlrichs , Phys. Chem. Chem. Phys. 2005, 7, 3297–3305. 16240044 10.1039/b508541a

[84] M. Valiev , E. J. Bylaska , N. Govind , K. Kowalski , T. P. Straatsma , H. J. J. Van Dam , D. Wang , J. Nieplocha , E. Apra , T. L. Windus , W. A. de Jong , Comput. Phys. Commun. 2010, 181, 1477–1489.

[85] A. Klamt , G. Schüürmann , J. Chem. Soc. Perkin Trans. 2 1993, 799–805.

[86] D. M. York , M. Karplus , J. Phys. Chem. A 1999, 103, 11060–11079.

[87] Q. Wu , T. Van Voorhis , Phys. Rev. A 2005, 72, 024502.

[88] N. Ando , M. Mitsui , A. Nakajima , J. Chem. Phys. 2007, 127, 234305. 18154380 10.1063/1.2805185

[89] P. Job , An. Chim. Fr. 1928, 9, 113–203.

[90] R. L. Scott , Recl. Trav. Chim. Pays-Bas 2010, 75, 787–789.

[91] B. K. Seal , H. Sil , D. C. Mukherjee , Spectrochim. Acta Part A 1982, 38, 289–292.

[92] J. C. Barnes , M. Juríček , N. L. Strutt , M. Frasconi , S. Sampath , M. A. Giesener , P. L. McGrier , C. J. Bruns , C. L. Stern , A. A. Sarjeant , J. F. Stoddart , J. Am. Chem. Soc. 2013, 135, 183–192. 22928610 10.1021/ja307360n

[93] M. Krishnamurthy , U. Muralikrishna , Spectrosc. Lett. 1988, 21, 277–283.

[94] R. Beukers , A. Szent-Györgyi , Recl. Trav. Chim. Pays-Bas 1962, 81, 255–268.

[95] F. B. Mallory , C. W. Mallory , K. E. Butler , M. B. Lewis , A. Q. Xia , E. D. Luzik, Jr. , L. E. Fredenburgh , M. M. Ramanjulu , Q. N. Van , M. M. Franci , D. A. Freed , C. C. Wray , C. Hann , M. Nerz-Stormes , P. J. Carroll , L. E. Chirlian , J. Am. Chem. Soc. 2000, 122, 4108–4116.

[96] J. E. Peralta , V. Barone , R. H. Contreras , D. G. Zaccari , J. P. Snyder , J. Am. Chem. Soc. 2001, 123, 9162–9163. 11552825 10.1021/ja011164y

[97] I. Alkorta , J. E. Elguero , Struct. Chem. 2004, 15, 117–120.

[98] T. Tuttle , J. Grafenstein , D. Cremer , Chem. Phys. Lett. 2004, 394, 5–13. 10.1063/1.1711598 15268014

[99] I. E. Kareev , G. S. Quinones , I. V. Kuvychko , P. A. Khavrel , I. N. Ioffe , I. V. Goldt , S. F. Lebedkin , K. Seppelt , S. H. Strauss , O. V. Boltalina , J. Am. Chem. Soc. 2005, 127, 11497–11504. 16089480 10.1021/ja051954y

[100] I. E. Kareev , I. V. Kuvychko , S. F. Lebedkin , S. M. Miller , O. P. Anderson , K. Seppelt , S. H. Strauss , O. V. Boltalina , J. Am. Chem. Soc. 2005, 127, 8362–8375. 15941270 10.1021/ja050305j

[101] J. M. Masnovi , E. A. Seddon , J. K. Kochi , Can. J. Chem. 1984, 62, 2552–2559.

[102] J. O. Howell , J. M. Gonclaves , C. Amatore , L. Klasinc , R. M. Wightman , J. K. Kochi , J. Am. Chem. Soc. 1984, 106, 3968–3976.

[103] E. Anxolabéhère , P. Hapiot , J.-M. Savéant , J. Electroanal. Chem. Interfacial Electrochem. 1990, 282, 275–280.

[104] V. D. Parker , J. Am. Chem. Soc. 1976, 98, 98–103.

[105] J. B. Torrance , J. E. Vazquez , J. J. Mayerle , V. Y. Lee , Phys. Rev. Lett. 1981, 46, 253–257.

[106] W. M. Nau , W. Adam , D. Klapstein , C. Sahin , H. Walter , J. Org. Chem. 1997, 62, 5128–5132, and references therein.

[107] R. S. Ruoff , K. M. Kadish , P. Boulas , E. C. M. Chen , J. Phys. Chem. 1995, 99, 8843–8850, and references therein.

[108] E. S. Chen , E. C. M. Chen , N. Sane , L. Talley , N. Kozanecki , S. Shulze , J. Chem. Phys. 1999, 110, 9319–9329, and references therein.

[109] L. D. Betowski , M. Enlow , L. Riddick , D. H. Aue , J. Phys. Chem. A 2006, 110, 12927–12946, and references therein. 17125310 10.1021/jp065785v

[110] A. Modelli , L. Mussoni , D. Fabbri , J. Phys. Chem. A 2006, 110, 6482–6486, and references therein. 16706405 10.1021/jp0605911

[111] G. Berionni , J. M. Gonçalves , C. Mathieu , T. Devic , A. Etchéberry , R. Goumont , Phys. Chem. Chem. Phys. 2011, 13, 2857–2869. 21165467 10.1039/c0cp01282c

[112] D. D. Méndez-Hernández , P. Tarakeshwar , D. Gust , T. A. Moore , A. L. Moore , V. Mujica , J. Mol. Model. 2013, 19, 2845–2848, and references therein. 23224940 10.1007/s00894-012-1694-7

[113] J. Calbo , R. Viruela , E. Ortí , J. Aragó , ChemPhysChem 2016, 17, 3881–3890. 27595419 10.1002/cphc.201600778

[114] T. T. Clikeman , E. V. Bukovsky , X.-B. Wang , Y.-S. Chen , G. Rumbles , S. H. Strauss , O. V. Boltalina , Eur. J. Org. Chem. 2015, 6641–6654.

[115] M. Lotfi , R. M. G. Roberts , Tetrahedron 1979, 35, 2123–2129.

[116] G. Paul , P. Kebarle , J. Am. Chem. Soc. 1989, 111, 464–470.

[117] M. Chowdhury , S. Basu , Trans. Faraday Soc. 1960, 56, 335–359.

[118] R. S. Mulliken , J. Am. Chem. Soc. 1952, 74, 811–824.

[119] R. S. Mulliken , J. Phys. Chem. 1952, 56, 801–822.

[120] R. S. Mulliken , W. B. Person , Ann. Rev. Phys. Chem. 1962, 13, 107–126, and references therein.

[121] H. Kuroda , M. Kobayashi , M. Kinoshita , S. Takemoto , J. Chem. Phys. 1962, 36, 457–462.

[122] C. J. Bender , Chem. Soc. Rev. 1986, 15, 475–502, and references therein.

[123] J. E. Frey , Appl. Spectrosc. Rev. 1987, 23, 247–283.

[124] E. Michoulier , N. Ben Amor , M. Rapacioli , J. A. Noble , J. Mascetti , C. Toubin , A. Simon , Phys. Chem. Chem. Phys. 2018, 20, 11941–11953, and references therein. 29667677 10.1039/c8cp01175c

[125] A. L. Farragher , F. M. Page , Trans. Faraday Soc. 1967, 63, 2369–2378.

[126] M. Batley , L. E. Lyons , Nature 1962, 196, 573–574.

[127] M. J. S. Dewar , A. R. Lepley , J. Am. Chem. Soc. 1961, 83, 4560–4563.

[128] E. C. M. Chen , W. E. Wentworth , J. Chem. Phys. 1975, 63, 3183–3191.

[129] S. Hashimoto , Tetrahedron 2000, 56, 6957–6963.

[130] C. G. S. Lima , T. D. Lima , M. Duarte , I. D. Jurberg , M. W. Paixao , ACS Catal. 2016, 6, 1389–1407.

[131] A. Zweig , J. E. Lehnsen , W. G. Hodgson , W. H. Jura , J. Am. Chem. Soc. 1963, 85, 3937–3939.

[132] R. Foster , T. J. Thomson , Trans. Faraday Soc. 1963, 59, 2287–2295.

[133] G. Briegleb , J. Czekalla , Angew. Chem. 1960, 72, 401–413.

[134] T. Akutagawa , M. Uchigata , T. Hasegawa , T. Nakamura , K. A. Nielsen , J. O. Jeppesen , T. Brimert , J. Becher , J. Phys. Chem. B 2003, 107, 13929–13938.

[135] Y. Yoshida , Y. Shimizu , T. Yajima , G. Maruta , S. Takeda , Y. Nakano , T. Hiramatsu , H. Kageyama , H. Yamochi , G. Saito , Chem. Eur. J. 2013, 19, 12313–12324. 23897669 10.1002/chem.201300578

[136] G.-Z. Zhu , L.-S. Wang , J. Chem. Phys. 2015, 143, 221102. 26671350 10.1063/1.4937761

[137] A. L. Farragher , F. M. Page , Trans. Faraday Soc. 1966, 62, 3072–3080.

[138] H. Nakashima , Y. Honda , T. Shida , H. Nakatsuji , Mol. Phys. 2015, 113, 1728–1739.

[139] K. Prout , I. J. Tickle , J. Chem. Soc. Perkin Trans. 2 1973, 1212–1215.

[140] J. D. Mottishaw , H. R. Sun , J. Phys. Chem. A 2013, 117, 7970–7979. 23906416 10.1021/jp403679x

[141] A. Putta , J. D. Mottishaw , Z. H. Wang , H. R. Sun , Cryst. Growth Des. 2014, 14, 350–356.

[142] M. O. BaniKhaled , J. D. Mottishaw , H. R. Sun , Cryst. Growth Des. 2015, 15, 2235–2242.

[143] A. S. Batsanov , J. C. Collings , J. A. K. Howard , T. B. Marder , D. F. Perepichka , Acta Crystallogr. Sect. C 2001, 57, 1306–1307. 11706258 10.1107/s0108270101013075

[144] H. Bock , M. Sievert , H. Schödel , M. Kleine , Z. Naturforsch. B 1996, 51, 1521–1537.

[145] H. Chen , F. Gao , E. D. Yao , Q. Chen , Y. G. Ma , CrystEngComm 2013, 15, 4413–4416.

[146] A. J. C. Buurma , O. D. Jurchescu , I. Shokaryev , J. Baas , A. Meetsma , G. A. de Wijs , R. A. de Groot , T. T. M. Palstra , J. Phys. Chem. C 2007, 111, 3486–3489.

