# Supplementary material for: PAH/PAH(CF3)n Donor/Acceptor Charge‐Transfer Complexes in Solution and in Solid‐State Co‐Crystals
Source: Chemistry. 2019 Oct 7;25(59):13547–65. doi: 10.1002/chem.201902712 (PMC6916568; doi:10.1002/chem.201902712)
Supplement: Supplementary file 1 — Supplementary [file CHEM-25-13547-s001.pdf]

CHEMISTRY
A European Journal

Supporting Information

PAH/PAH(CF_3)$_n$ Donor/Acceptor Charge-Transfer Complexes in Solution and in Solid-State Co-Crystals

Karlee P. Castro,[a] Eric V. Bukovsky,[a] Igor V. Kuvychko,[a] Nicholas J. DeWeerd,[a] Yu-Sheng Chen,[b] Shihu H. M. Deng,[c] Xue-Bin Wang,*[c] Alexey A. Popov,*[d] Steven H. Strauss,*[a] and Olga V. Boltalina*[a]

chem_201902712_sm_miscellaneous_information.pdf

SUPPORTING INFORMATION FOR

PAH/PAH(CF_3)$_n$ Donor/Acceptor Charge-Transfer Complexes in Solution and in Solid-State Co-Crystals

Karlee P. Castro, Eric V. Bukovsky, Igor V. Kuvychko, Nicholas J. DeWeerd,
Yu-Sheng Chen, Shihu H. M. Deng, Xue-Bin Wang, Alexey A. Popov,
Steven H. Strauss, and Olga V. Boltalina

Notes: The generic abbreviation PAH(CF_3)$_n$ denotes a compound with n H atoms replaced by n CF_3 groups (e.g., the composition of ANTH(CF_3)$_6$ is $C_{14}H_4(CF_3)_6$, not $C_{14}H_{10}(CF_3)_6$). The references cited in this document are collected at the end of this document; their numbers are not the same as the number of the same reference in the main text.

PAH abbreviations: ANTH, anthracene; AZUL, azulene; CORO, coronene;
PERY, perylene; PYRN, pyrene; TRPH, triphenylene

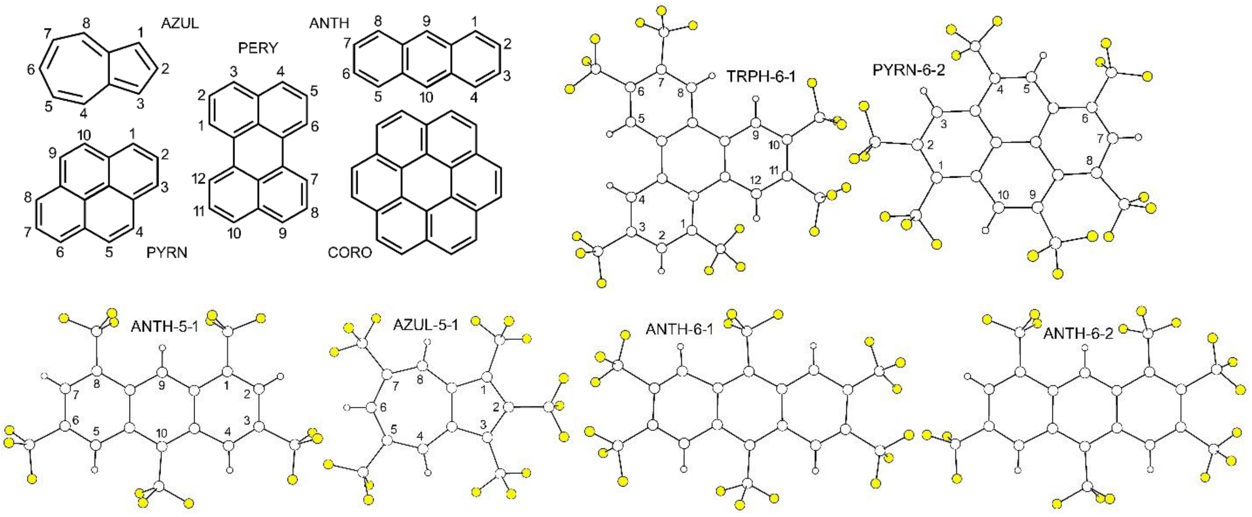

Table of Contents

	page
**Table S1.** All PAH(CF_3)$_n$ compounds reported in the literature to date	4
**Figure S1.** The ^{1}H and ^{19}F NMR spectra of ANTH-6-2	7
**Figure S2.** The ^{1}H and ^{19}F NMR spectra of PYRN-6-2	8
**Figure S3.** Cyclic voltammograms of ANTH-6-2, PYRN-6-2, and CORO	9
**Figure S4.** Near UV spectra of ANTH and ANTH-6-1	10
**Figure S5.** Photographs of solutions or crystals of some PAH/PAH(CF_3)$_n$ CT complexes	11
**Figure S6.** Electronic spectra of CT complexes in the solid state	12
**Figure S7.** Scott and Seal plots for titrations of ANTH-5-1 and ANTH-6-1 with ANTH	13
**Figure S8.** Solution electronic spectra of five PAH/PAH(CF_3)$_n$ CT complexes	14
**Table S2.** Solvent effect on the $E(\lambda_{max})$ for the piperidine/$C_6H_2(CN)_4$ CT complex	15

Table S3. Solvent effect on the $E(\lambda_{max})$ for the PYRN/chloranil CT complex 16
Table S4. DFT energies for 1:1 PAH/PAH(CF_3)$_n$ charge-transfer complexes 17
Figure S9 Graphical representations of DFT results for PAH/PAH(CF_3)$_n$ CT complexes (two pages) 18
Figure S10. Thermal ellipsoid plots for the structures of ANTH/(ANTH-5-1)$_2$ and ANTH/ANTH-6-1 20
Figure S11. Thermal ellipsoid plots for the structures of PERY/ANTH-6-1 and (CORO)$_2$/ANTH-5-1 21
Figure S12. Thermal ellipsoid plots for the structures of PYRN/(TRPH-6-1)$_2$ and the published structures of PYRN/(ANTH-6-1)$_2$ and PYRN/AZUL-5-1 22
Figure S13. Drawings of portions of the D/A/D/A... infinite stacks in ANTH/ANTH-6-2, PERY/ANTH-6-1, PYRN/AZUL-5-1, PYRN/PYRN-6-2, and PYRN/(TRPH-6-1)$_2$ 23
Figure S14. Perpendicular drawings of the array of D/A/D/A... stacks in ANTH/ANTH-6-1 24
Figure S15. Perpendicular drawings of the array of D/A/A/D/A/A... stacks in ANTH/(ANTH-5-1)$_2$ 25
Figure S16. Perpendicular drawings of the array of D/A/D/A... stacks in ANTH/ANTH-6-2 26
Figure S17. Perpendicular drawings of the array of D/D/A/D/D/A... stacks in (CORO)$_2$/ANTH-6-1 27
Figure S18. Perpendicular drawings of the array of D/A/D/A... stacks in PYRN/PYRN-6-2 28
Figure S19. Perpendicular drawings of the array of D/A/D/A... stacks in PYRN/AZUL-5-1 29
Figure S20. "Perpendicular" drawings of the array of D/A/A/D/A/A... stacks in PYRN/(TRPH-6-1)$_2$ 30
Figure S21. Drawings of the π–π overlap in the structure of ANTH/(ANTH-5-1)$_2$ 31
Figure S22. Drawings of the π–π overlap in the structure of ANTH/ANTH-6-2 32
Figure S23. Drawings of π–π overlap in the PYRN/AZUL-5-1 and PYRN/(TRPH-6-1)$_2$ structures 33
Figure S24. Drawings of the π–π overlap in the structure of PYRN/PYRN-6-2 34
Figure S25. Comparison of π–π overlap in the four structures with PYRN as the donor in this work 35
Figure S26. Comparison of the (TRPH)$_2$ pairs in PYRN/(TRPH)$_2$ and in TRPH-6-1 36
Table S5. PAH donor *IE*s and *EA*s 37
Figure S27. PAH *IE* vs. $E_{1/2}$(+/0) plotted from literature data (Pysh/Yang and Kochi et al.) 38
Table S6. PAH(CF_3)$_n$ and other strong electron acceptor *EA*s 39
Figure S28. PAH G3(MP2) *EA* vs. $E_{1/2}$(0/−) plotted from literature data (Calbo/Aragó) 40
Table S7. Equilibrium constants for D/A CT complexes with D = anthracene (ANTH) 41
Figure S29 PAH *IE* vs. PAH/TCPA $E(\lambda_{max})$ plotted from literature data (Chowdhury/Basu) 42
Figure S30. PAH *IE* vs. PAH/acceptor $E(\lambda_{max})$ plotted from literature data (Foster/Thompson, Briegleb/Czekalla, and Zweig et al.) 43
Figure S31. Acceptor *EA* vs. PYRN/acceptor $E(\lambda_{max})$ plotted from literature data (Farragher/Page) 44
Figure S32. Acceptor *EA* vs. D/A $E(\lambda_{max})$ plotted from literature data (Yoshida et al. and Akutagawa, Nakamura, et al.) 45
Figure S33. Electronic spectra of the ANTH/ANTH-6-1 and ANTH/TCNQ CT complexes in CH_2Cl_2 46
Table S8. Donor/acceptor interplanar distances defined two different ways 47

Figure S34. The recently published structure of PYRN/(ANTH-6-1)$_2$ 48

Figure S35. Plot of $E(\lambda_{max})$ vs. $\Delta(IE/EA)$ for the four PYRN/PAH(CF_3)$_n$ CT complexes 49

Figure S36. Side views of the structures of PYRN/AZUL-5-1, PYRN/(ANTH-6-1)$_2$, PYRN/PYRN-6-2, and PYRN/(TRPH)$_2$ 50

Figure S37. Definition of the ANTH bend angle θ and flat projections perpendicular to the least-squares planes of the aromatic cores in the four donor/ANTH-6-1 X-ray structures 51

Figure S38. Comparison of the DFT optimized and X-ray structures of the PYRN/ANTH-6-1 charge-transfer complexes 52

Figure S39. Comparison of the DFT optimized and X-ray structures of the ANTH/ANTH-6-1 charge-transfer complexes 53

Figure S40. Comparison of the DFT optimized and X-ray structures of the CORO/ANTH-6-1 charge-transfer complexes 54

Figure S41. Comparison of the DFT optimized and X-ray structures of the PERY/ANTH-6-1 charge-transfer complexes 55

Figure S42. Comparison of the DFT optimized and X-ray structures of the ANTH/ANTH-5-1 charge-transfer complexes 56

Figure S43. Comparison of the X-ray structures of ANTH/(ANTH-5-1)$_2$ and ANTH/NAPH(F)$_8$ 57

Figure S44. The D/A π–π overlap in the X-ray structures of PYRN/chloroanil, ANTH/TCNB, ANTH/(ANTH-5-1)$_2$, and ANTH/ANTH-6-1 58

Figure S45. The D/A π–π overlap in the X-ray structures of CORO/(MeO)$_2$TCNQ, ANTH/NBDF, 9-ANTH(Me)/TCNB, PERY/Br_4BQ, CORO/TCNQ, PYRN/F_4BQ, and TETR/TCNQ 59

Figure S46. DFT-predicted ΔE_{CT} as a function of interplanar spacing 60

Figure S47. CORO/CORO π–π overlap in (CORO)$_2$/ANTH-6-1, (CORO)$_2$/F_4TCNQ, and pure CORO 61

Figure S48. Molecular stacking and packing in the structures of (CORO)$_2$/ANTH-6-1 (CORO)$_2$/F_4TCNQ, and pure CORO at 100 K 62

Supporting Information References 63

Table S1. PAH(CF_3)$_n$ compounds reported in the literature to date[a]

compd	abbreviation	*EA*, eV	X-ray structure	ref.
9-ANTH(CF_3)	ANTH-1-1	—	no	[1-2]
9,10-ANTH(CF_3)$_2$	ANTH-2-1	—	no	[3]
1,3,6,8-ANTH(CF_3)$_4$	ANTH-4-1	—	yes	[4]
1,3,6,8,10-ANTH(CF_3)$_5$	ANTH-5-1	2.40(2)	yes	[5]
2,3,6,7,9,10-ANTH(CF_3)$_6$	ANTH-6-1	2.81(2)	yes	[5]
2,3,6,7,9,10-ANTH(CF_3)$_6$	ANTH-6-2	2.68(2)	yes	this work
1,2,4,5,7,8-ANTH(CF_3)$_6$[b]	ANTH-6-3	—	no	[4]
2,3,6,7,9,10-ANTH(CF_3)$_6$[b]	ANTH-6-4	—	no	[4]
1,3,5-AZUL(CF_3)$_3$[b]	AZUL-3-1	—	no	[6]
1,3,6-AZUL(CF_3)$_3$[b]	AZUL-3-2	—	no	[6]
1,2,3-AZUL(CF_3)$_3$[b]	AZUL-3-2	—	no	[6]
1,3,5,7-AZUL(CF_3)$_4$	AZUL-4-1	2.49(1)	yes	[6]
1,2,3,5-AZUL(CF_3)$_4$	AZUL-4-2	2.50(1)	yes	[6]
1,2,3,5,7-AZUL(CF_3)$_5$	AZUL-5-1	2.850(15)	yes	[6]
1-CORA(CF_3)	CORA-1-1[c]	—	no	[7]
1,2-CORA(CF_3)$_2$	CORA-2-1	—	yes	[7]
1.4.7-CORA(CF_3)$_3$[b]	CORA-3-2[d]	—	no	[8]
1,2,7,8-CORA(CF_3)$_2$	CORA-4-1	—	no	[9]
1,2,6,8-CORA(CF_3)$_4$	CORA-4-2	—	yes	[8]
1,3,5,7,9-CORA(CF_3)$_5$	CORA-5-1	2.215(15)	yes	[10]
1,3,5,8,10-CORA(CF_3)$_5$[b]	CORA-5-2	—	no	[8]
1,2,4,6,8-CORA(CF_3)$_5$	CORA-5-3	—	yes	[8]
1,2,3,5,7,9-CORA(CF_3)$_6$[b]	CORA-6-1	—	no	[8]
1,2,4,5,7,9-CORA(CF_3)$_6$	CORA-6-2	—	yes	[8]
1,2,4,6,7,9-CORA(CF_3)$_6$	CORA-6-3	—	yes	[8]
1,2,3,5,6,8,9-CORA(CF_3)$_7$	CORA-7-1	—	yes	[8]
13,14-DAPP(CF_3)$_2$	DAPP-2-1	—	yes	[11]
9-FLRA(CF_3)[b]	FLRA-1-1	—	no	[12]
8,9- FLRA(CF_3)$_2$[b]	FLRA-2-1	—	no	[12]
1,4,7-FLRA(CF_3)$_3$[b]	FLRA-3-1	—	no	[4]
1,3,6-FLRA(CF_3)$_3$[b]	FLRA-3-1	—	no	[4]

Table S1. PAH(CF_3)$_n$ compounds reported in the literature to date (continued)[a]

compd	abbreviation	*EA*, eV	X-ray structure	ref.
3,8,9-FLRA(CF_3)$_3$[b]	FLRA-3-3	—	no	[12]
1,3,6,8-FLRA(CF_3)$_4$[b]	FLRA-4-1	2.28(1)	no	[4]
3,4,8,9-FLRA(CF_3)$_4$[b]	FLRA-4-2	—	no	[12]
3,5,8,9-FLRA(CF_3)$_4$[b]	FLRA-4-3	—	no	[12]
2,3,5,7,9-FLUR(CF_3)$_5$[b]	FLUR-5-1	—	no	[4]
1-NAPH(CF_3)	NAPH-1-1	—	no	[13]
2-NAPH(CF_3)	NAPH-2-1	—	no	[13-14]
1,3,5,7-NAPH(CF_3)$_4$	NAPH-4-1	1.80(2)	yes	[15-16]
2,3,6,7-NAPH(CF_3)$_4$	NAPH-4-2	—	no	[17]
2-QUIN(CF_3)	QUIN-1-1	—	no	[18]
3-QUIN(CF_3)	QUIN-1-2	—	no	[18]
6,13-PENT(CF_3)$_2$[b]	PENT-2-1	—	yes	[19-20]
PENT(CF_3)$_8$ [mixture of isomers]	PENT-8	3.32(2)	no	[5]
1,4,7,10-PERY(CF_3)$_4$	PERY-4-1	2.20(2)	yes	[5]
1,3,6,8,10-PERY(CF_3)$_5$	PERY-5-1	2.46(2)	yes	[5]
[locants unknown]	PERY-5-2	2.48(2)	no	[5]
[locants unknown]	PERY-5-3	2.49(2)	no	[5]
[locants unknown]	PERY-5-4	2.45(2)	no	[5]
1,3,5,7,9,11-PERY(CF_3)$_6$	PERY-6-1	2.72(2)	yes	[5]
PERY(CF_3)$_7$ [mixture of isomers]	PERY-7	2.91(2)[c]	no	[5]
1,3,6,8-PHEN(CF_3)$_4$	PHEN-4-1	—	no	[4]
1,3,6,9-PHEN(CF_3)$_4$	PHEN-4-2	—	no	[4]
1,3,6,7,9-PHEN(CF_3)$_5$	PHEN-5-1	1.95(1)	yes	[5]
1,6-PHNZ(CF_3)$_2$	PHNZ-2-1	2.00(2)[e]	yes	[21]
1,4-PHNZ(CF_3)$_2$	PHNZ-2-2	2.00(2)[e]	no	[21]
1,3,9-PHNZ(CF_3)$_3$	PHNZ-3-1	2.37(2)	yes	[21]
1,4,6-PHNZ(CF_3)$_3$	PHNZ-3-1	2.30(2)	yes	[21]
1,3,6,9-PHNZ(CF_3)$_4$	PHNZ-4-1	2.65(1)	yes	[21]
1,4,6,9-PHNZ(CF_3)$_4$	PHNZ-4-2	2.60(1)	yes	[21]
1,2,4,6,9-PHNZ(CF_3)$_5$	PHNZ-5-1	2.93(1)	yes	[21]

Table S1. $PAH(CF_3)_n$ compounds reported in the literature to date (continued)[a]

compd	abbreviation	*EA*, eV	X-ray structure	ref.
1,3,4,6,7,9-PHNZ$(CF_3)_6$	PHNZ-6-1	3.24(2)[e]	yes	[21]
1,2,4,6,7,9-PHNZ$(CF_3)_6$[b]	PHNZ-6-2	3.24(2)[e]	no	[21]
1,3,4,6,8-PYRN$(CF_3)_5$	PYRN-5-1	2.44(2)	yes	[5]
1,3,4,6,9-PYRN$(CF_3)_5$	PYRN-5-2	2.38(2)	yes	[5]
1,3,5,8,9-PYRN$(CF_3)_5$[b]	PYRN-5-3	—	no	[4]
1,3,4,6,8,9-PYRN$(CF_3)_6$	PYRN-6-1	2.71(2)	yes	[5]
1,2,4,6,8,9-PYRN$(CF_3)_6$	PYRN-6-2	—	yes	this work
1,3,6,7,10,11-TRPH$(CF_3)_6$	TRPH-6-1	2.11(2)	yes	[5]

[a] PAH derivatives with substituents other than CF_3 groups are not listed in this table. Abbreviations: *EA*, experimental gas-phase electron affinity; ANTH, anthracene; AZUL, azulene; CORA, corannulene; DAPP = 5,8-diazapentaphene = dibenzo[*b*,*j*][4,7]phenanthroline; FLRA, fluoranthene; FLUR, fluorene; NAPH, naphthalene; PENT, pentacene; PERY, perylene; PHEN, phenanthrene; PHNZ, phenazine; PYRN, pyrene; QUIN, quinoline; TRPH, triphenylene. [b] Tentative locants based on NMR spectroscopy. [c] This is the only possible isomer of $CORA(CF_3)$. [d] A hypothetical isomer calculated by DFT was labeled CORA-3-1 in ref. [8]. [e] Mixture of isomers.

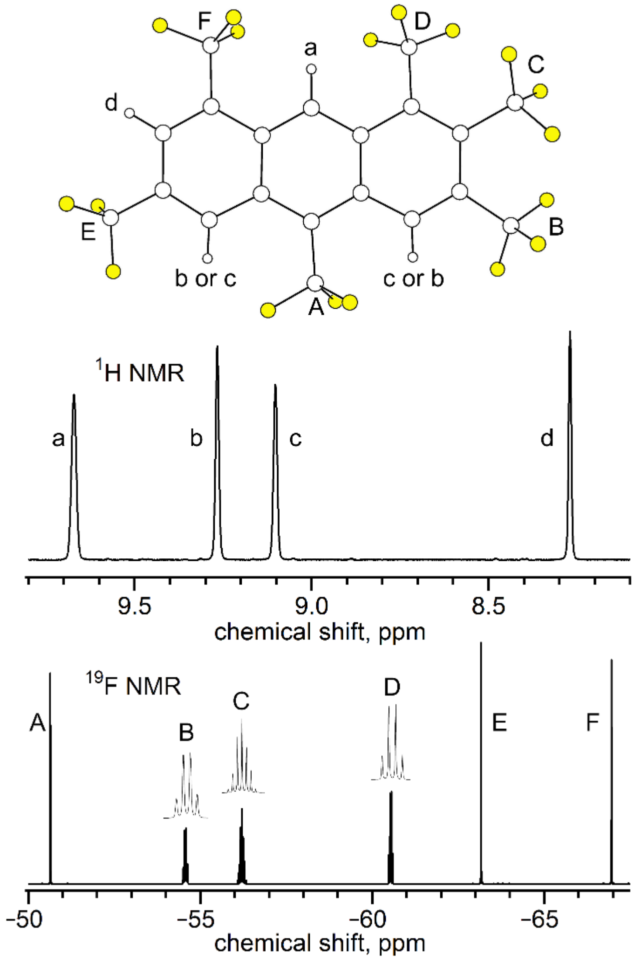

Figure S1. The ^{1}H and ^{19}F NMR spectra of ANTH-6-2 in $CDCl_3$. The resonance and multiplet assignments are based on the NMR spectra of the symmetric derivatives ANTH-5-1 and ANTH-6-1 published in ref. [5].

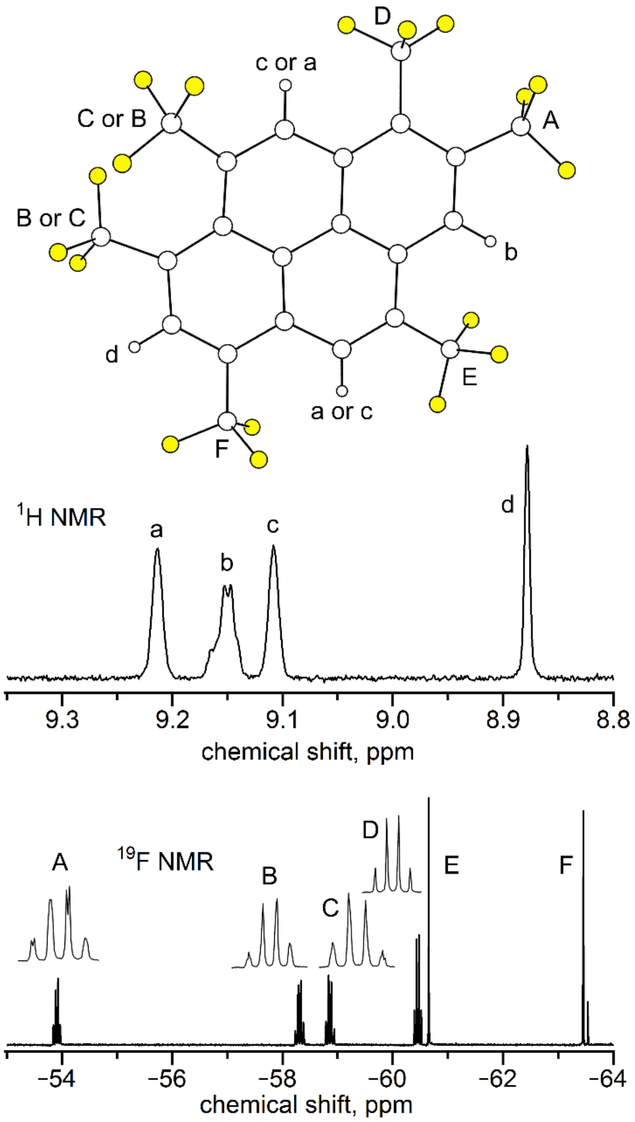

Figure S2. The ^{1}H and ^{19}F NMR spectra of PYRN-6-2 in $CDCl_3$. The resonance and multiplet assignments are based on the NMR spectra of the symmetric derivatives PYRN-5-1 and PYRN-6-1 published in ref. [5].

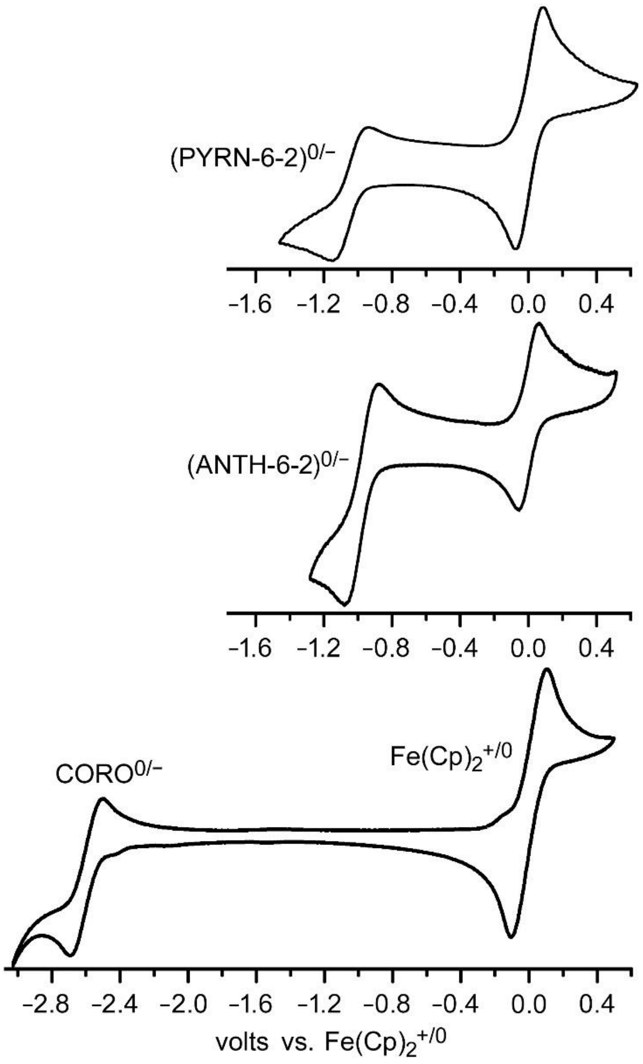

Figure S3. Cyclic voltammograms of PYRN-6-2, ANTH-6-2, and CORO, showing the quasi-reversible first reduction of each compound (dimethoxyethane, 0.10 M $N(n\text{-}Bu)_4ClO_4$, $Fe(Cp)_2$ internal standard, 500 mV s^{-1}).

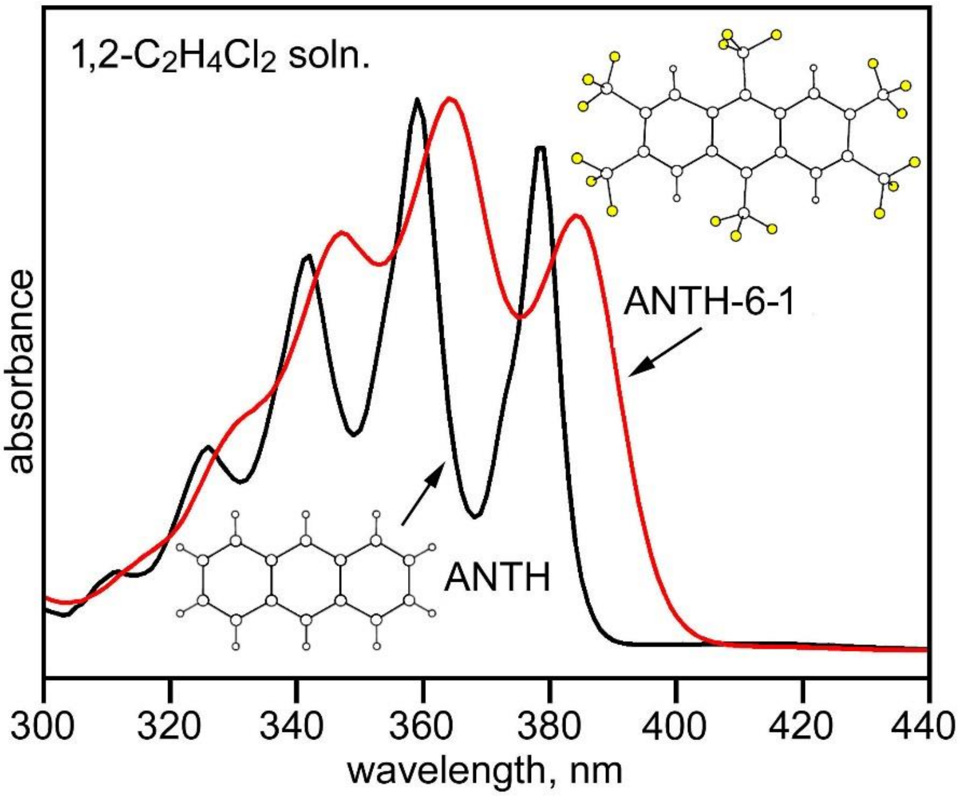

Figure S4. The near UV spectra of ANTH and ANTH-6-1 in 1,2-$C_2H_4Cl_2$. Note that the HOMO-LUMO gap in ANTH-6-1 is just slightly smaller than for ANTH. The λ_{max} values in the spectrum of ANTH are 379, 359, 342, and 326 nm. The λ_{max} values in the spectrum of ANTH-6-1 are 384, 364, and 347 nm. A similar figure was recently published in ref. [22]. The concentrations of the samples for both spectra were ca. 2 mM.

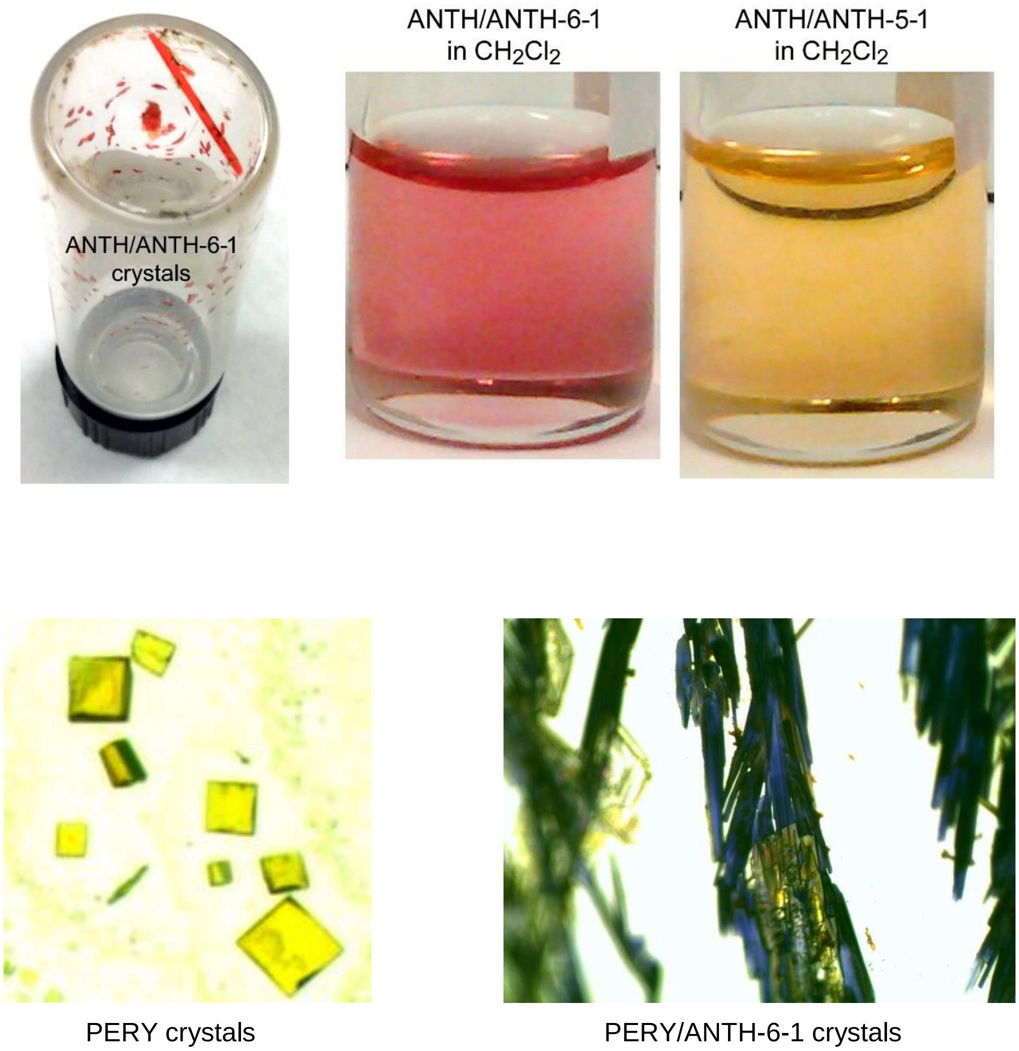

Figure S5. Crystals of the ANTH/ANTH-6-1 CT complex co-crystals and CH_2Cl_2 solutions of the ANTH/ANTH-6-1 and ANTH/ANTH-5-1 CT complexes (top). Crystals of PERY and the PERY/ANTH-6-1 CT complex co-crystals (bottom).

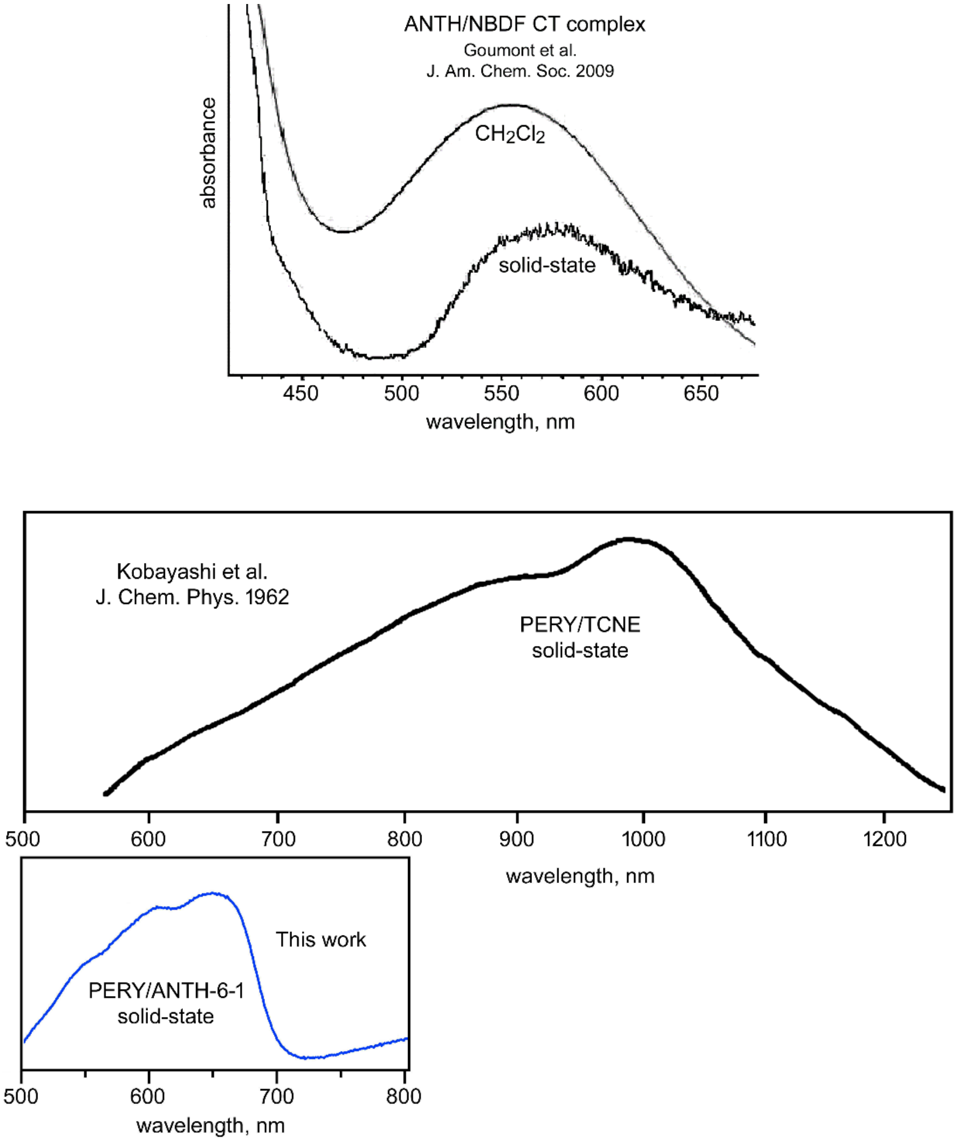

Figure S6. Top: Electronic spectra of the CT complex ANTH/NBDF in CH_2Cl_2 and in the solid state (NBDF = 4-nitrobenzodifuroxan). This figure was modified from the figure in ref. [23]. Bottom: Electronic spectra of the CT complexes PERY/TCNE (modified from ref. [24]) and PERY/ANTH-6-1 in the solid state (this work).

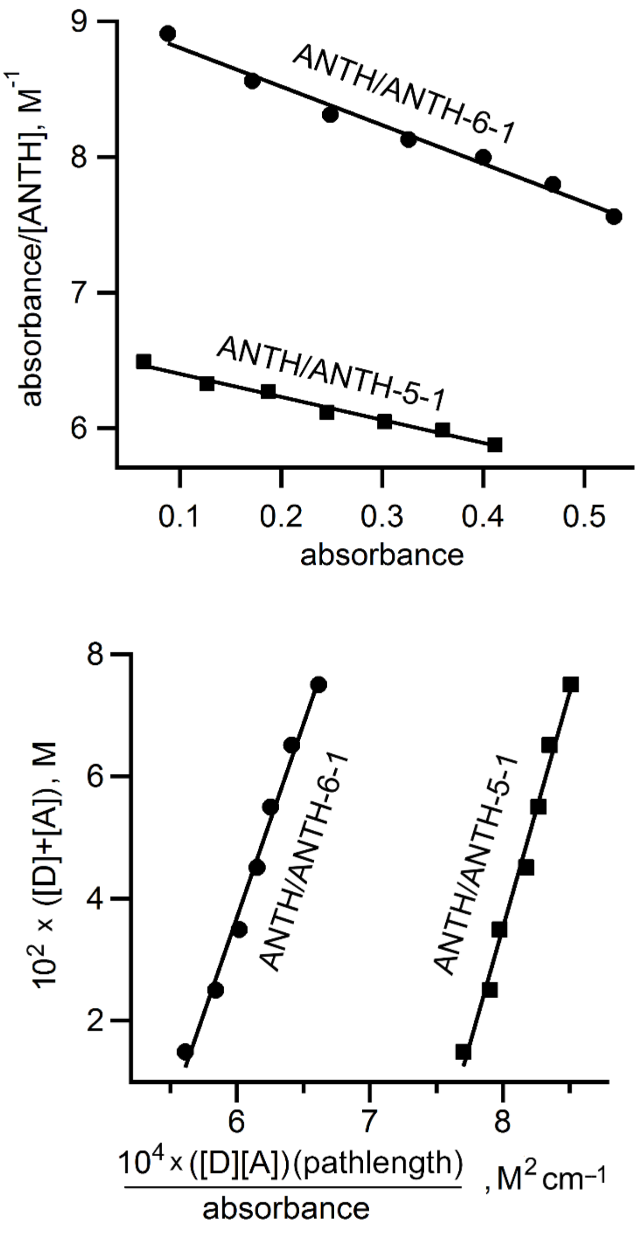

Figure S7. Scott plots (top) and Seal plots (bottom) for titrations of ANTH-5-1 and ANTH-6-1 with ANTH in 1,2-dichloroethane. The concentration of ANTH-5-1 and ANTH-6-1 was 5.0 mM and the concentration of ANTH was varied between 10 and 70 mM. The slope of each Scott plot is equal to $-K_{eq}$ for the D + A $\rightleftharpoons$ D/A equilibrium. The slope of each Seal plot is the molar extinction coefficient, ε, for the CT band λ_{max}. The K_{eq} values for ANTH/ANTH-5-1 and ANTH/ANTH-6-1 are 1.7(1) and 2.8(1) M^{-1}, respectively. The ε and λ_{max} values for the ANTH/ANTH-5-1 and ANTH/ANTH-6-1 CT bands are 7.7(4) $\times 10^2$ cm^{-1} M^{-1} (λ_{max} = 467 nm) and 6.3(3) $\times 10^2$ cm^{-1} M^{-1} (λ_{max} = 523 nm), respectively.

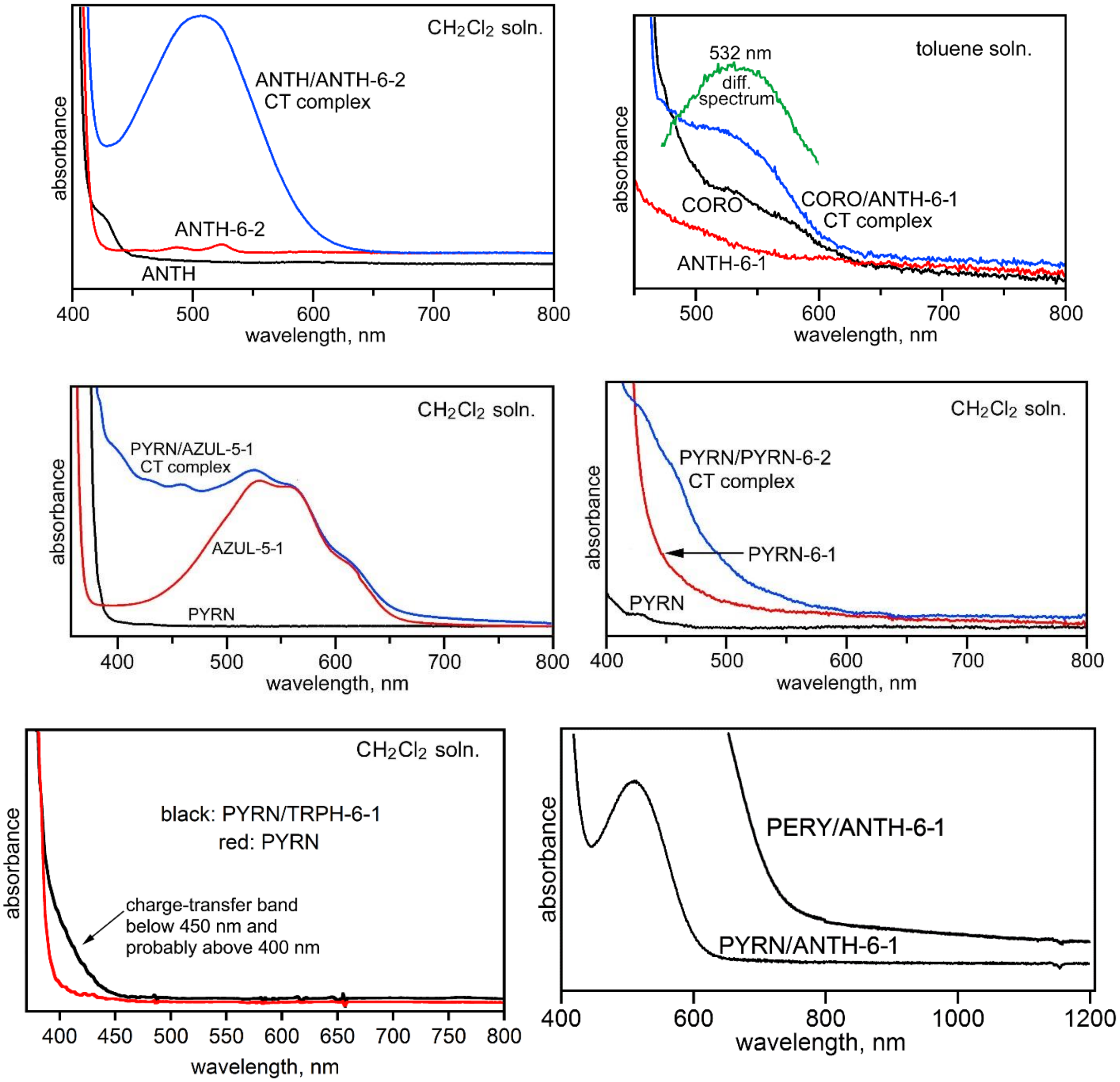

Figure S8. Electronic spectra of the ANTH/ANTH-6-2, PYRN/AZUL-5-1, PYRN/PYRN-6-2, and PYRN/TRPH-6-1 CT complexes in dichloromethane and of CORO/ANTH-6-1 in toluene. The spectra of the individual PAH donor (D) and PAH(CF_3)$_n$ acceptor (A) are also shown. For convenience these are written as 1/1 D/A complexes in solution, although that was not verified. For the CORO/ANTH-6-1 panel of spectra, part of the difference spectrum (blue spectrum minus black spectrum) is shown in green (λ_{max} = 532 nm). The difference spectrum is not to scale in the absorbance dimension. Spectral subtraction did not result in a difference spectrum with an obvious CT band λ_{max} for PYRN/AZUL-5-1 or PYRN/TRPH-6-1. The spectrum of PYRN-6-1 is shown in the panel with the spectrum of PYRN/PYRN-6-2 because the electronic spectrum of PYRN-6-2 was not recorded. The lower right pair of spectra show that no spectral bands were observed between 800–1200 nm for PYRN/ANTH-6-1 and PERY/ANTH-6-1 in dichloromethane. This was also true for the other solution spectra, but the other plots are shown only out to 800 nm for simplicity. The concentrations of the samples for these spectra were ca. 5 mM.

Table S2. Solvent effect on the CT band λ_{max} value for the piperidine/tetrachloro-1,4-benzoquinone donor/acceptor complex[a]

solvent	λ_{max} ($cm^{-1}/1000$)	λ_{max} (nm)	λ_{max} (eV)
n-C_6H_{14}	17.79	562	2.206
C_6H_6	17.69	565	2.193
$O(C_2H_5)_2$	17.65	567	2.188
$CH_3C(O)OC_2H_5$	17.54	570	2.175
1,2-$C_2H_4Cl_2$	17.53	570	2.173
CH_2Cl_2	17.50	571	2.170
1,2-$C_6H_4Cl_2$	17.48	572	2.167
CH_3OH	17.41	574	2.159
$CH_3C(O)CH_3$	17.41	574	2.159

[a] All data from ref. [25]. The CT band λ_{max} is 6 nm higher (0.02 eV lower) in CH_2Cl_2 than in C_6H_6. Tetrachloro-1,4-benzoquinone = Cl_4BQ = chloranil.

Table S3. Solvent effect on the CT band λ_{max} value for the pyrene/chloranil donor/acceptor complex[a]

solvent	λ_{max} (cm^{-1}/1000)	λ_{max} (nm)	λ_{max} (eV)
$CHCl_3$	16.20	617	2.009
CH_2Cl_2	16.40	610	2.033
CCl_4	16.50	606	2.046
C_6H_6	16.80	595	2.083
toluene	17.00	588	2.108
THF	17.50	571	2.170
$CH_3C(O)OC_2H_5$	17.70	565	2.195
DMSO	17.90	559	2.219
CH_3OH	17.90	559	2.219
$CH_3C(O)CH_3$	18.00	556	2.232

[a] All data from ref. [26]. The CT band λ_{max} is 22 nm higher (0.08 eV lower) in CH_2Cl_2 than in toluene and 6 nm (0.02 eV lower) in CH_2Cl_2 than in $CHCl_3$.

Table S4. DFT energies (eV) for donor (D) and acceptor (A) reactions for 1:1 PAH/PAH$(CF_3)_n$ charge-transfer complexes[a]

reaction	ANTH/ANTH-5-1	ANTH/ANTH-6-1	ANTH/ANTH-6-2	PERY/ANTH-6-1
$D \rightarrow D^+$	7.07	7.07	7.07	6.60
$D \rightarrow D_{soln}$	−0.19	−0.19	−0.19	−0.26
$D_{soln} \rightarrow D_{soln}^+$	5.44	5.44	5.44	5.17
$A \rightarrow A^-$	−2.44	−2.87	−2.73	−2.87
$A \rightarrow A_{soln}$	−0.27	−0.33	−0.29	−0.33
$A_{soln} \rightarrow A_{soln}^-$	−3.40	−3.77	−3.69	−3.77
$D^+ + A^- \rightarrow D^+A^-$	−2.81	−2.56	−2.64	−2.61
$D^+A^- \rightarrow (D^+A^-)_{soln}$	−0.89[b]	−0.92[b]	−0.94[b]	−0.91[b]
$D + A \rightarrow DA$	−0.74	−0.79	−0.82	−0.97
$DA \rightarrow D^+A^-$	2.70[b]	2.43[b]	2.52	2.09[b]
$D_{soln} + A_{soln} \rightarrow (DA)_{soln}$	−0.63	−0.67	−0.72	−0.79
$(DA)_{soln} \rightarrow (D^+A^-)_{soln}$	2.16[b]	1.91[b]	1.96	1.59[b]
$D_{soln}^+ + A^-_{soln} \rightarrow (D^+A^-)_{soln}$	−0.51	−0.42	−0.52	−0.60

reaction	CORO/ANTH-6-1	PYRN/ANTH-6-1	PYRN/AZUL-6-1	PYRN/PYRN-6-2	PYRN/TRPH-6-1
$D \rightarrow D^+$	7.07	7.12	7.12	7.12	7.12
$D \rightarrow D_{soln}$	−0.25	−0.20	−0.20	−0.20	−0.20
$D_{soln} \rightarrow D_{soln}^+$	5.68	5.54	5.54	5.54	5.54
$A \rightarrow A^-$	−2.87	−2.87	−2.77	−2.67	−2.12
$A \rightarrow A_{soln}$	−0.33	−0.33	−0.29	−0.30	−0.41
$A_{soln} \rightarrow A_{soln}^-$	−3.77	−3.77	−3.83	−3.59	−2.97
$D^+ + A^- \rightarrow D^+A^-$	−2.66	−2.54	−2.45	−2.54	−2.60
$D^+A^- \rightarrow (D^+A^-)_{soln}$	−0.88[b]	−0.88[b]	−0.92[b]	−0.90[b]	−0.98[b]
$D + A \rightarrow DA$	−1.03	−0.83	−0.79	−0.82	−0.91
$DA \rightarrow D^+A^-$	2.57[b]	2.54[b]	2.68[b]	2.73[b]	3.30[b]
$D_{soln} + A_{soln} \rightarrow (DA)_{soln}$	−0.87	−0.67	−0.68	−0.69	−0.79
$(DA)_{soln} \rightarrow (D^+A^-)_{soln}$	2.11[b]	2.03[b]	2.15[b]	2.20[b]	2.82[b]
$D_{soln}^+ + A^-_{soln} \rightarrow (D^+A^-)_{soln}$	−0.67	−0.41	−0.22	−0.42	−0.60

[a] All calculations by B3LYP-D3/def2-TZVPP unless othewise indicated. Some of the values in this table are also listed in Table 2 in the main text. The gas-phase *IE* and *EA* values are for adiabatic processes. [b] Calculations by B3LYP-D3/6-311G* because of convergence problems when using the def2-TZVPP basis set.

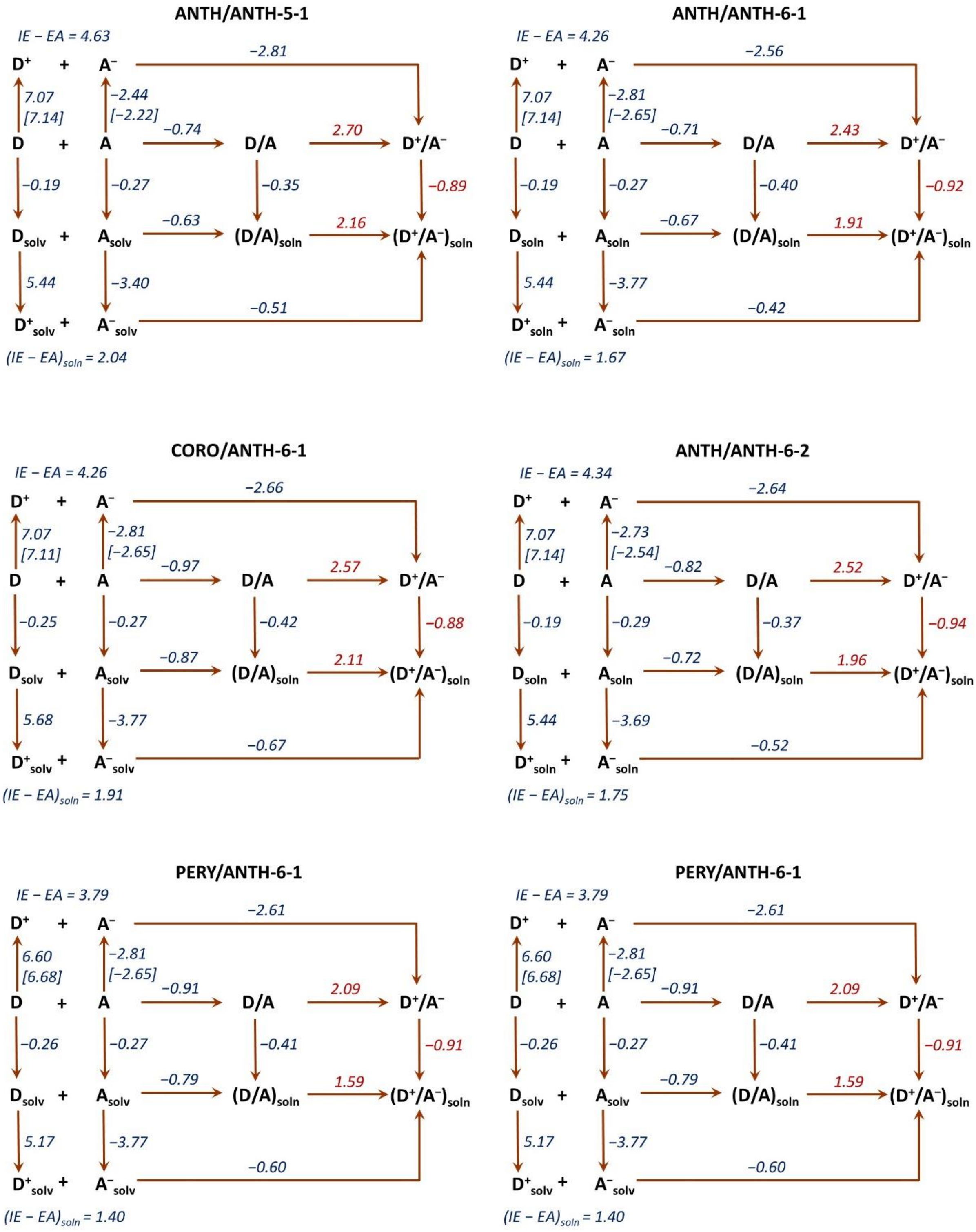

[**Figure S9** (in part). See next page for figure caption]

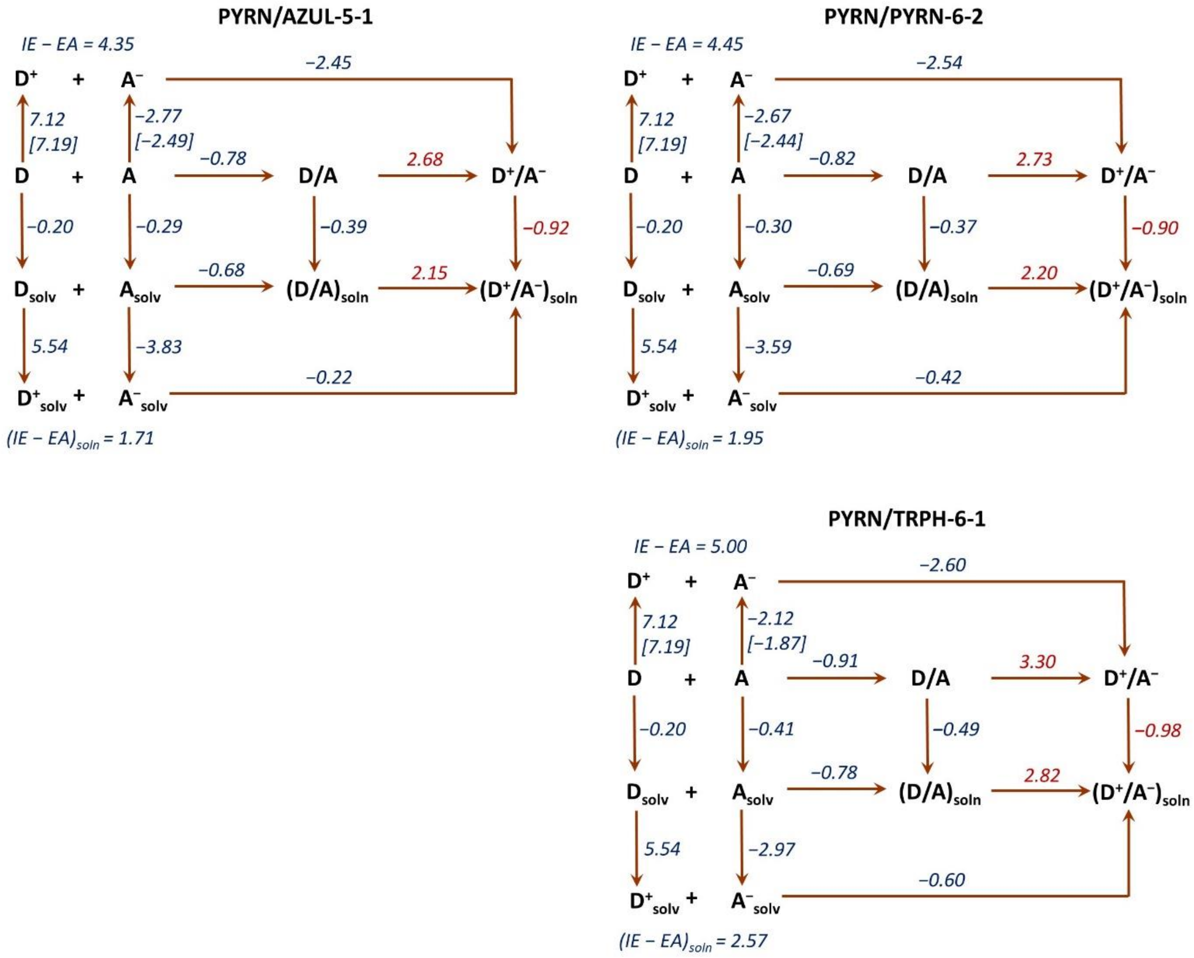

Figure S9. Graphical representations of DFT-predicted energy changes (eV) for reactions of PAH electron donors (D) and PAH(CF_3)$_n$ electron acceptors (A) and their D/A CT complexes that were studied in this work. The species are either in the gas-phase (not labeled) or in solution (labeled soln) with a dielectric continuum equivalent to 1,2-dichloroethane (ε = 10.4). All calculations were B3LYP-D3/def2-TZVPP except D/A → D^+/A^- and $(D/A)_{soln}$ → $(D^+/A^-)_{soln}$ (highlighted in red), which were B3LYP-D3/6-311G* because of convergence problems when using the def2-TZVPP basis set. The *IE* and *EA* values in square brackets are for vertical processes.

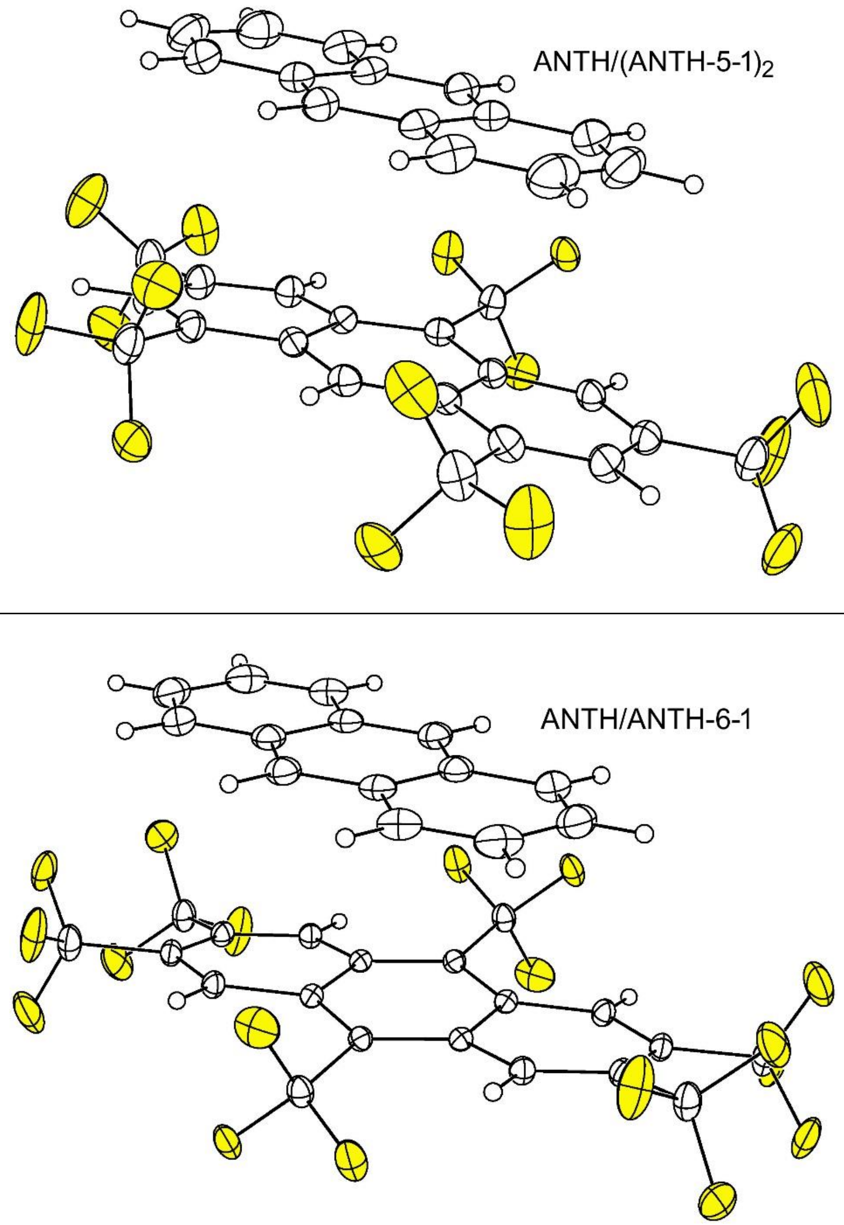

Figure S10. Thermal ellipsoid plots of a single donor/acceptor pair in the CT complex co-crystal structures of ANTH/(ANTH-5-1)$_2$ (top) and ANTH/ANTH-6-1 (bottom; 50% probability ellipsoids except for H atoms; F atoms shaded yellow). The structure of ANTH/(ANTH-5-1)$_2$ exhibits hexagonal arrays of infinite D/A/A/D/A/A... stacks of parallel donors and acceptors. The structure of ANTH/ANTH-6-1 exhibits hexagonal arrays of infinite alternating D/A/D/A... stacks of parallel donors and acceptors.

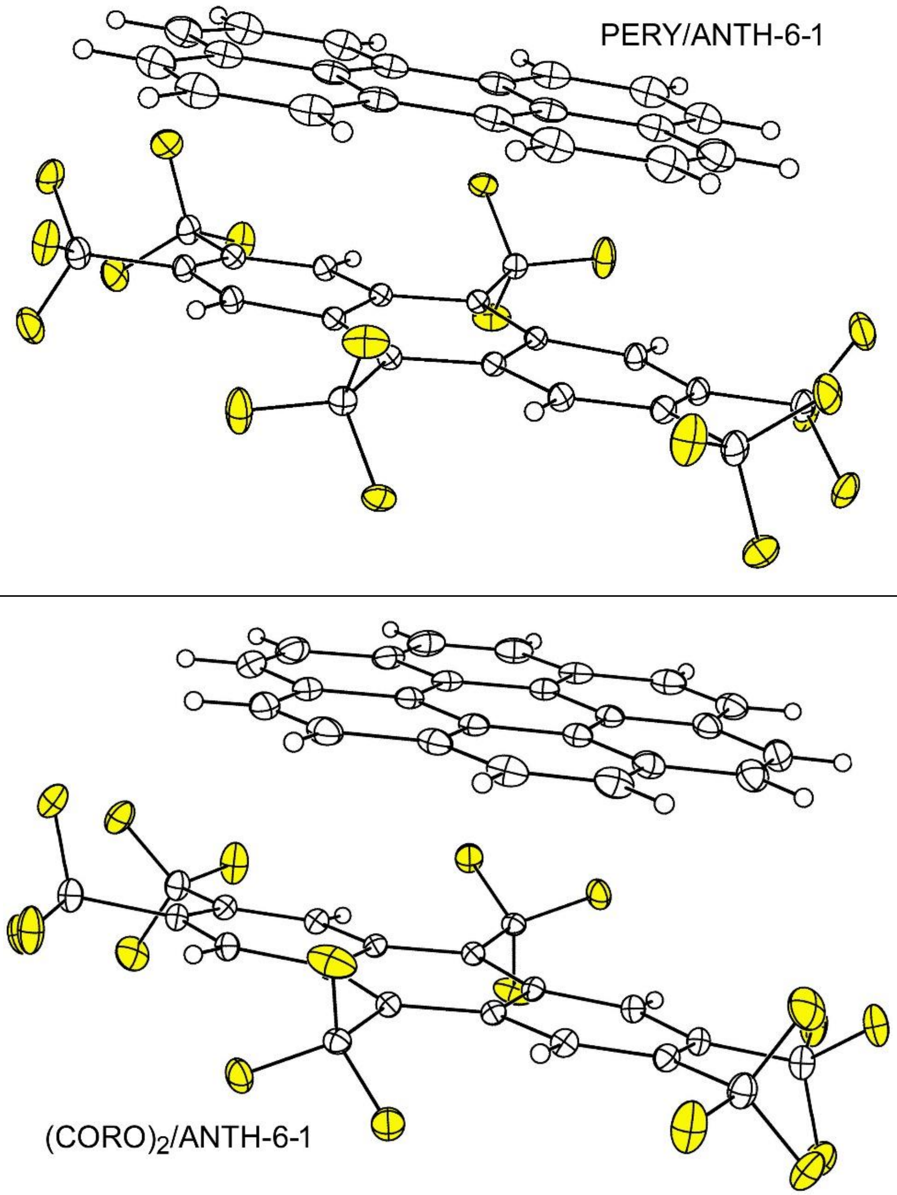

Figure S11. Thermal ellipsoid plots of a single donor/acceptor pair in the CT complex co-crystal structures of PERY/ANTH-6-1 (top) and $(CORO)_2$/ANTH-6-1 (bottom; 50% probability ellipsoids except for H atoms; F atoms shaded yellow). The structure of PERY/ANTH-6-1 exhibits hexagonal arrays of infinite alternating D/A/D/A... stacks of parallel donors and acceptors. The structure of $(CORO)_2$/ANTH-6-1 exhibits hexagonal arrays of infinite D/D/A/D/D/A... stacks of parallel donors and acceptors.

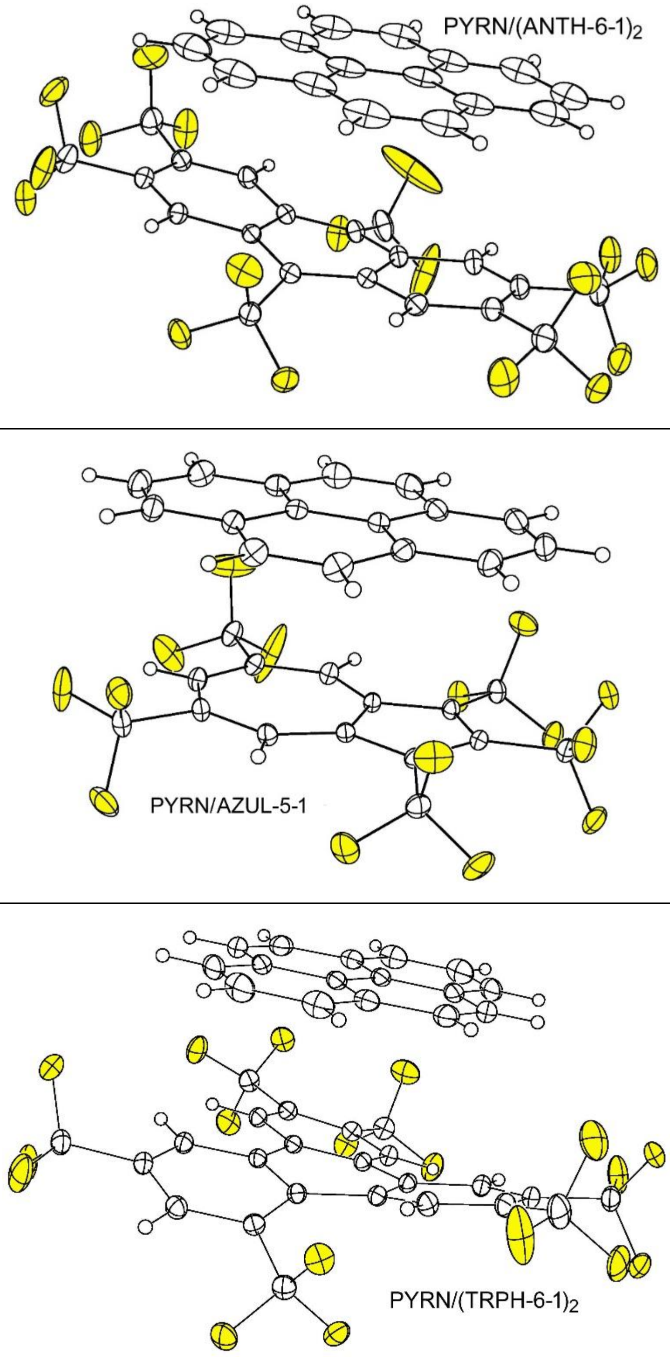

Figure S12. Thermal ellipsoid plots (50% probability ellipsoids except for H atoms; F atoms shaded yellow) of a single donor/acceptor pair in the CT complex co-crystal structures of PYRN/(ANTH-6-1)$_2$ (top, ref. [22]), PYRN/AZUL-5-1 (middle, ref. [6]), and PYRN/(TRPH-6-1)$_2$ (bottom, this work). The structure of PYRN/(ANTH-6-1)$_2$ exhibits discrete {A/D/A} sandwiches of parallel donor and acceptor molecules. The structures of PYRN/AZUL-5-1 and PYRN/(TRPH-6-1)$_2$ exhibit hexagonal arrays of infinite stacks of nearly parallel donors and acceptors.

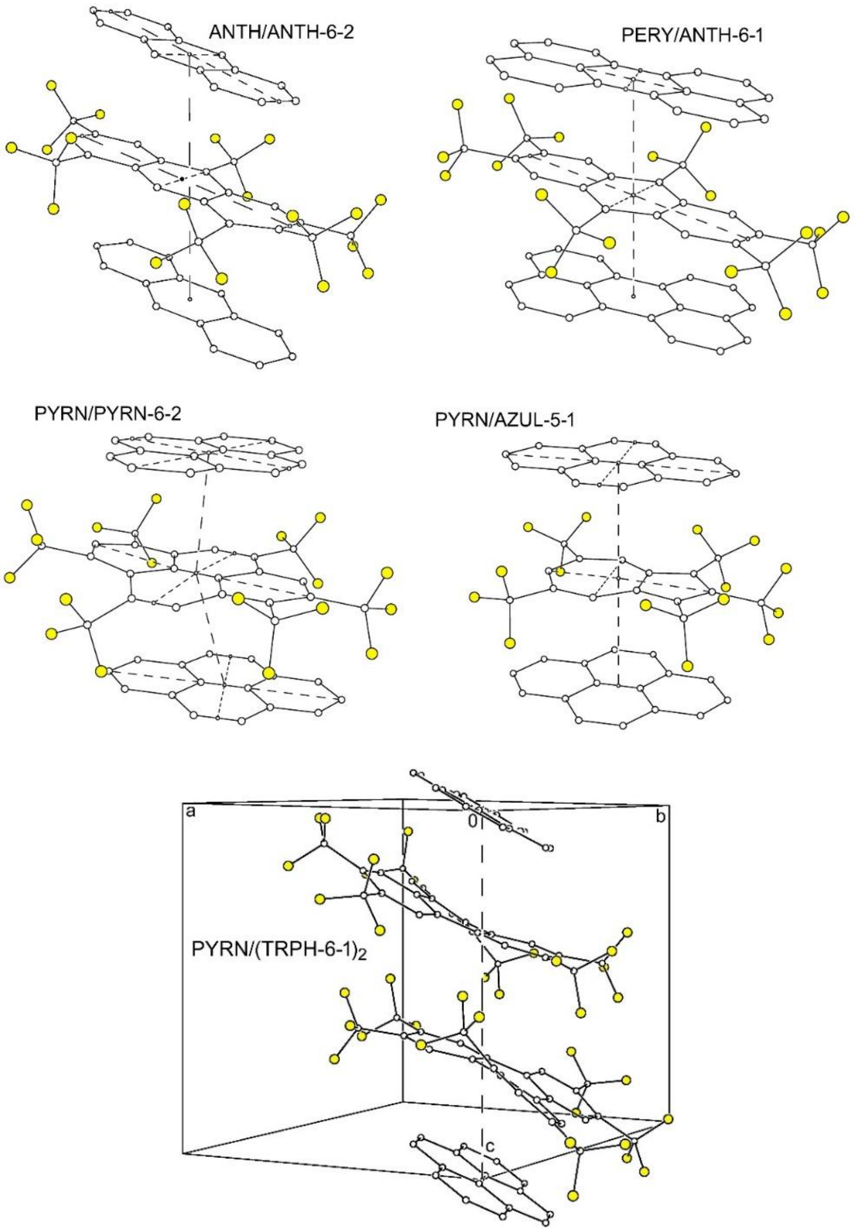

Figure S13. Drawings of portions of the structures ANTH/ANTH-6-2, PERY/ANTH-6-1, PYRN/AZUL-5-1, PYRN/PYRN-6-2 and PYRN/(TRPH-6-1)$_2$ showing the nearly parallel donors and acceptors (H atoms omitted for clarity; F atoms shaded yellow). The smallest circles represent either bond or hexagon centroids. The dihedral angles between the least squares planes of the aromatic cores of each donor/acceptor pair of molecules are 1.9°, 1.0°, 0.8°, 3.1°, and 4.5° respectively. The rotations of the donor major axes with respect to the acceptor major axes are 14.8°, 23.2°, 3.9°, and 22.8/85.2° for the first four structures, respectively (note: there are two angles in PYRN/PYRN-6-2). The TRPH-6-1 acceptor does not have a clearly defined major axis.

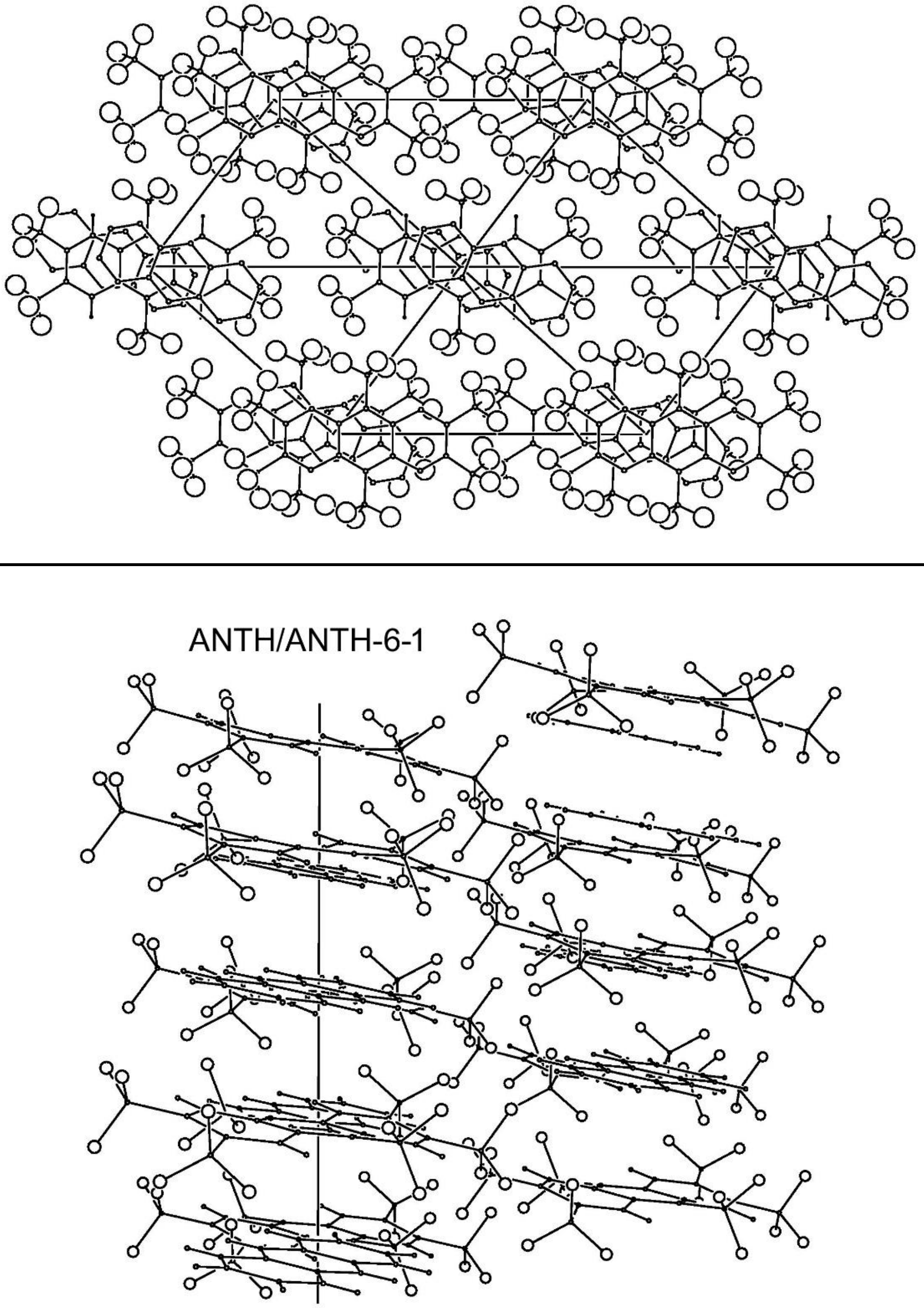

Figure S14. Perpendicular drawings of the hexagonal array of infinite alternating D/A/D/A... stacks of nearly parallel ANTH (D) and ANTH-6-1 (A) molecules in the CT complex co-crystal structure of ANTH/ANTH-6-1. The larger spheres in each drawing are F atoms. The vertical line in the lower drawing is perpendicular to the plane of the hexagon in the upper drawing. The ANTH molecules in all of the stacks are parallel.

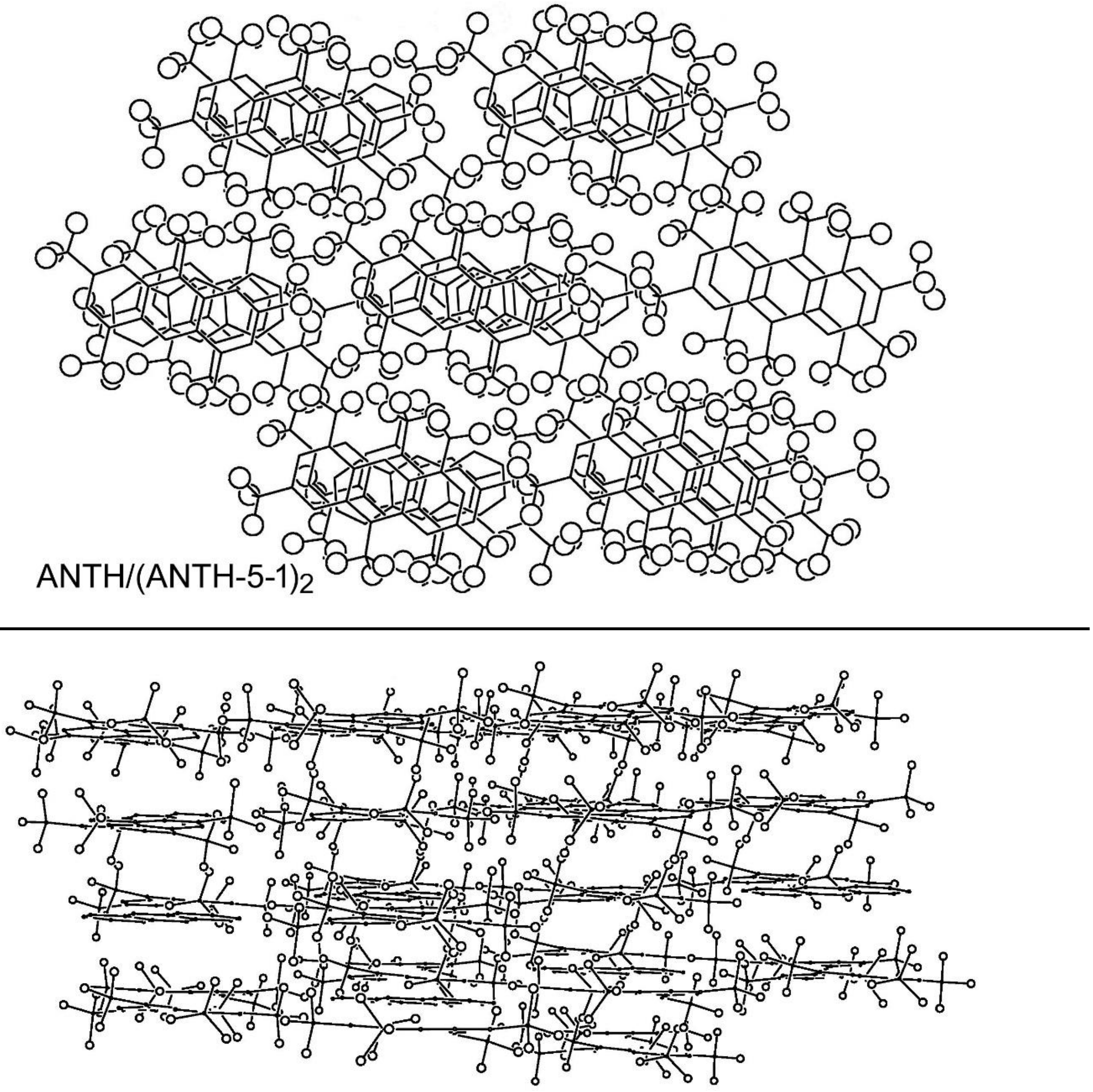

Figure S15. Perpendicular drawings of the hexagonal array of infinite alternating D/A/A/D/A/A... stacks of nearly parallel ANTH (D) and ANTH-5-1 (A) molecules in the CT complex co-crystal structure of ANTH/(ANTH-5-1)$_2$ (H atoms omitted for clarity). The larger spheres in each drawing are F atoms. The ANTH molecules in all of the stacks are essentially parallel.

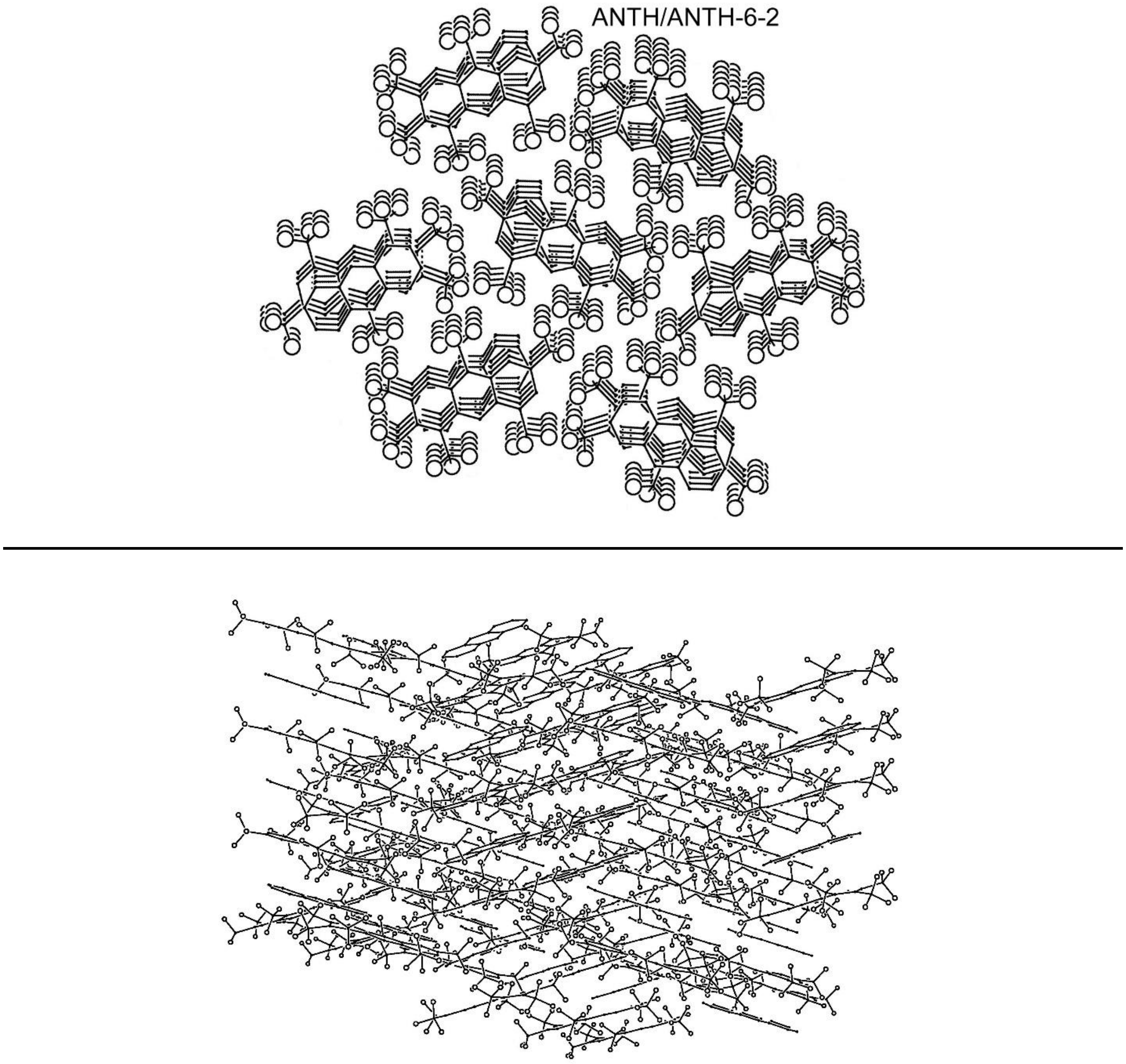

Figure S16. Perpendicular drawings of the hexagonal array of infinite alternating D/A/D/A... stacks of nearly parallel ANTH (D) and ANTH-6-2 (A) molecules in the CT complex co-crystal structure of ANTH/ANTH-6-2 (H atoms omitted for clarity). The larger spheres in each drawing are F atoms. The ANTH and ANTH-6-2 molecules in half of the stacks are tilted 34.6° with respect to the ANTH and ANTH-6-2 molecules in the other stacks.

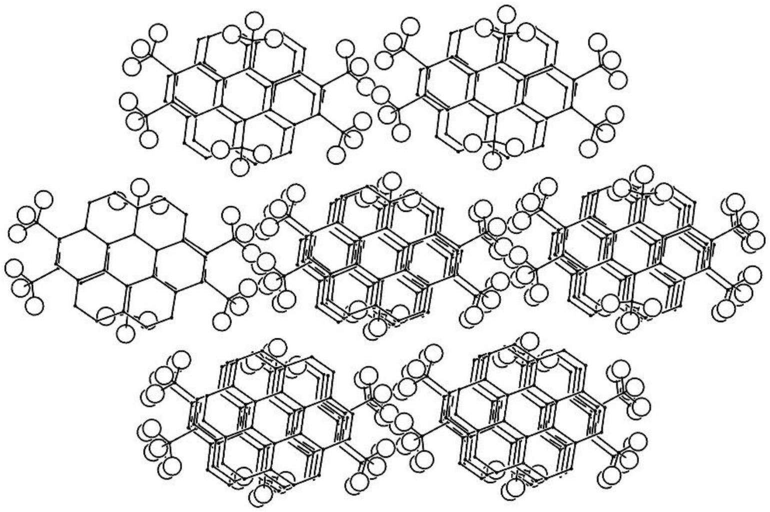

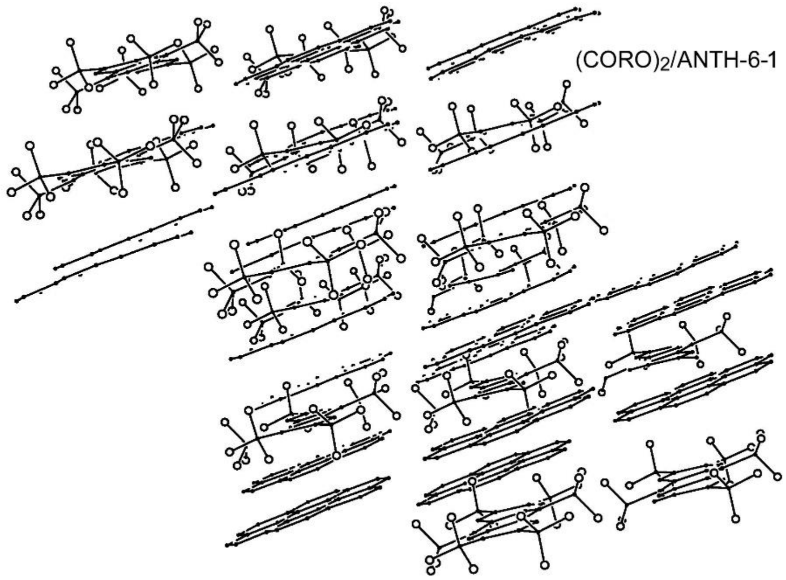

Figure S17. Perpendicular drawings of the hexagonal array of infinite alternating D/D/A/D/D/A... stacks of nearly parallel CORO (D) and ANTH-6-1 (A) molecules in the CT complex co-crystal structure of $(CORO)_2$/ANTH-6-1 (H atoms omitted for clarity). The larger spheres in each drawing are F atoms. The CORO molecules in all of the stacks are parallel.

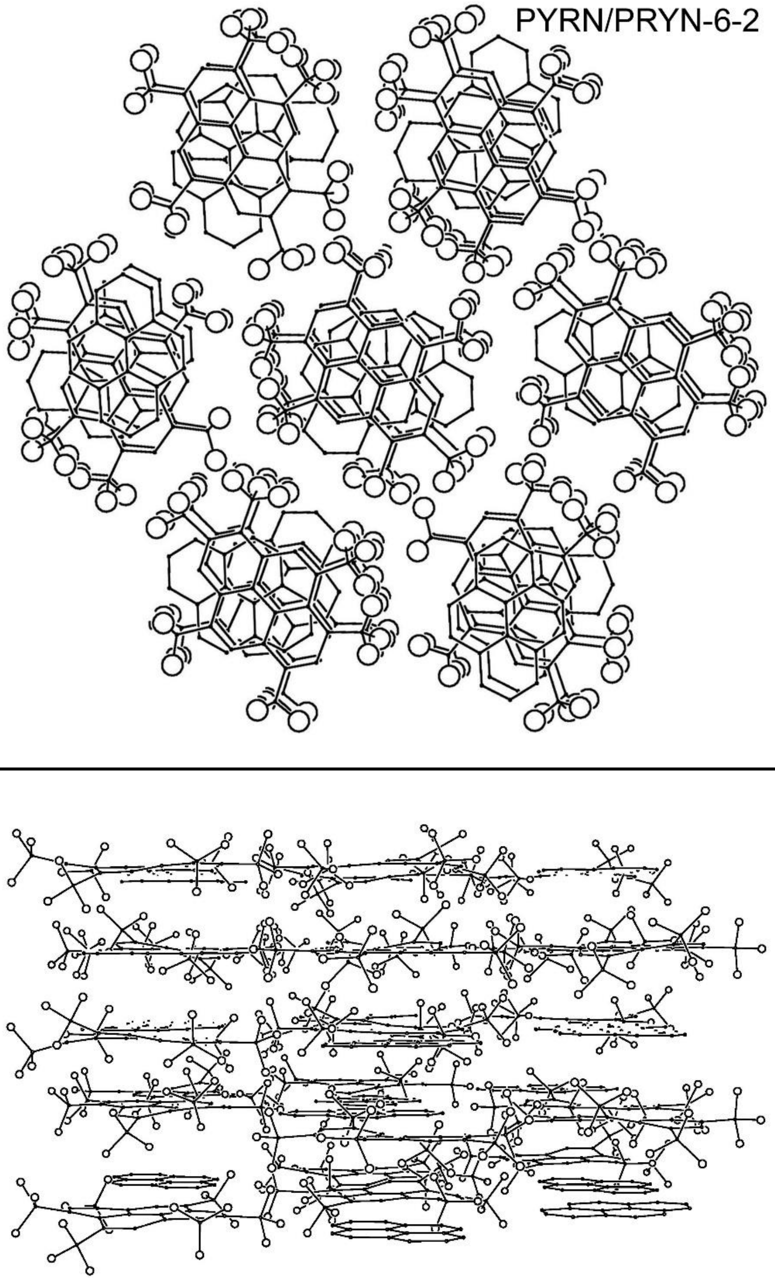

Figure S18. Perpendicular drawings of the hexagonal array of infinite alternating D/A/D/A... stacks of nearly parallel PYRN (D) and PYRN-6-2 (A) molecules in the CT complex co-crystal structure of PYRN/PYRN-6-2 (H atoms omitted for clarity). The larger spheres in each drawing are F atoms. The PYRN molecules in all of the stacks are essentially parallel.

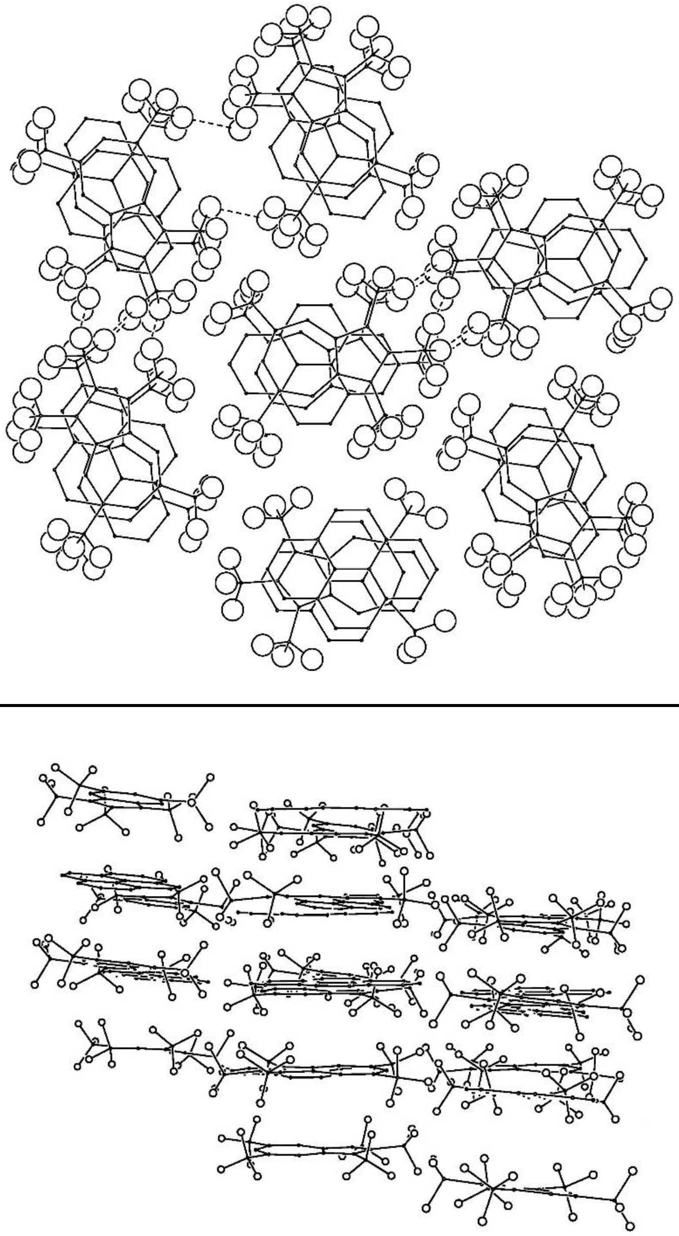

Figure S19. Perpendicular drawings of the hexagonal array of infinite alternating D/A/D/A... stacks of nearly parallel PYRN (D) and AZUL-5-1 (A) molecules in the CT complex co-crystal structure of PYRN/AZUL-5-1 (H atoms omitted for clarity). The larger spheres in each drawing are F atoms. The PYRN molecules within the stacks and between the stacks are essentially parallel. This structure was first reported in ref. [6].

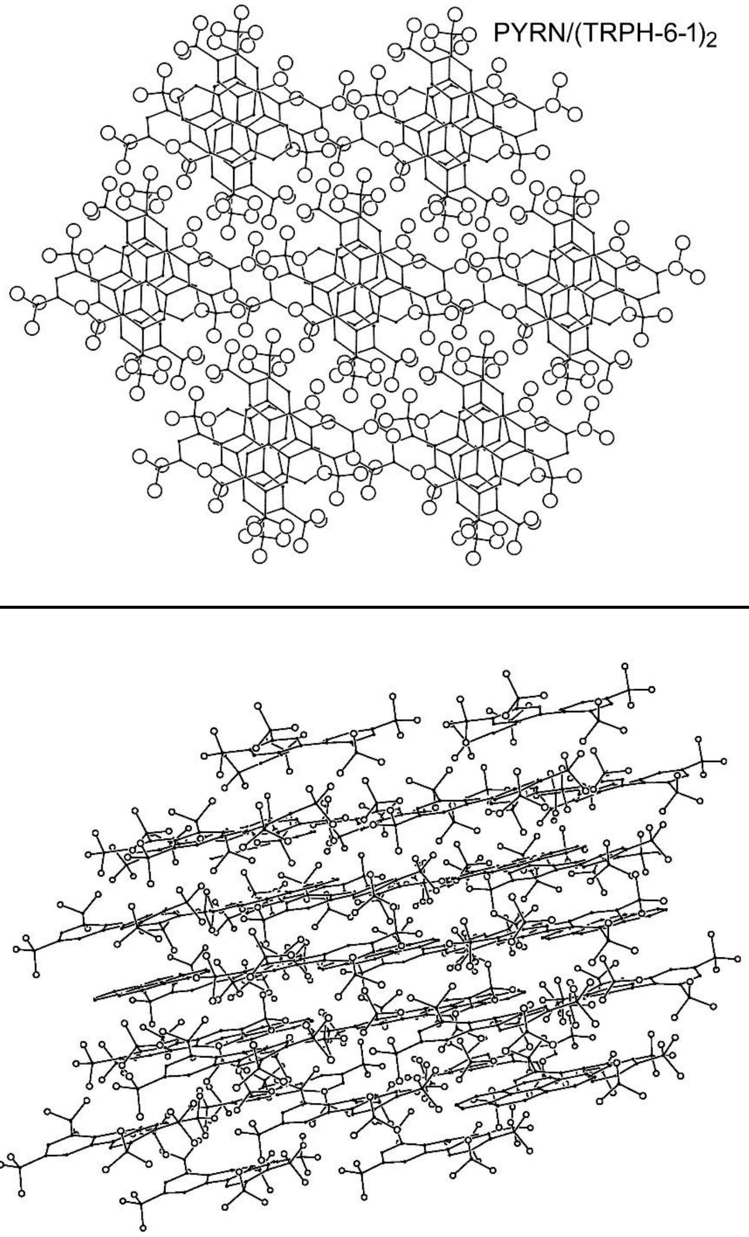

Figure S20. Drawings of the hexagonal array of infinite alternating D/A/A/D/A/A... stacks of nearly parallel PYRN (D) and TRPH-6-1 (A) molecules in the CT complex co-crystal structure of PYRN/(TRPH-6-1)$_2$ (H atoms omitted for clarity). The lower drawing has been tilted by 113° relative to the upper drawing. The larger spheres in both drawings are F atoms. The PYRN molecules within the stacks and between the stacks are parallel to one another.

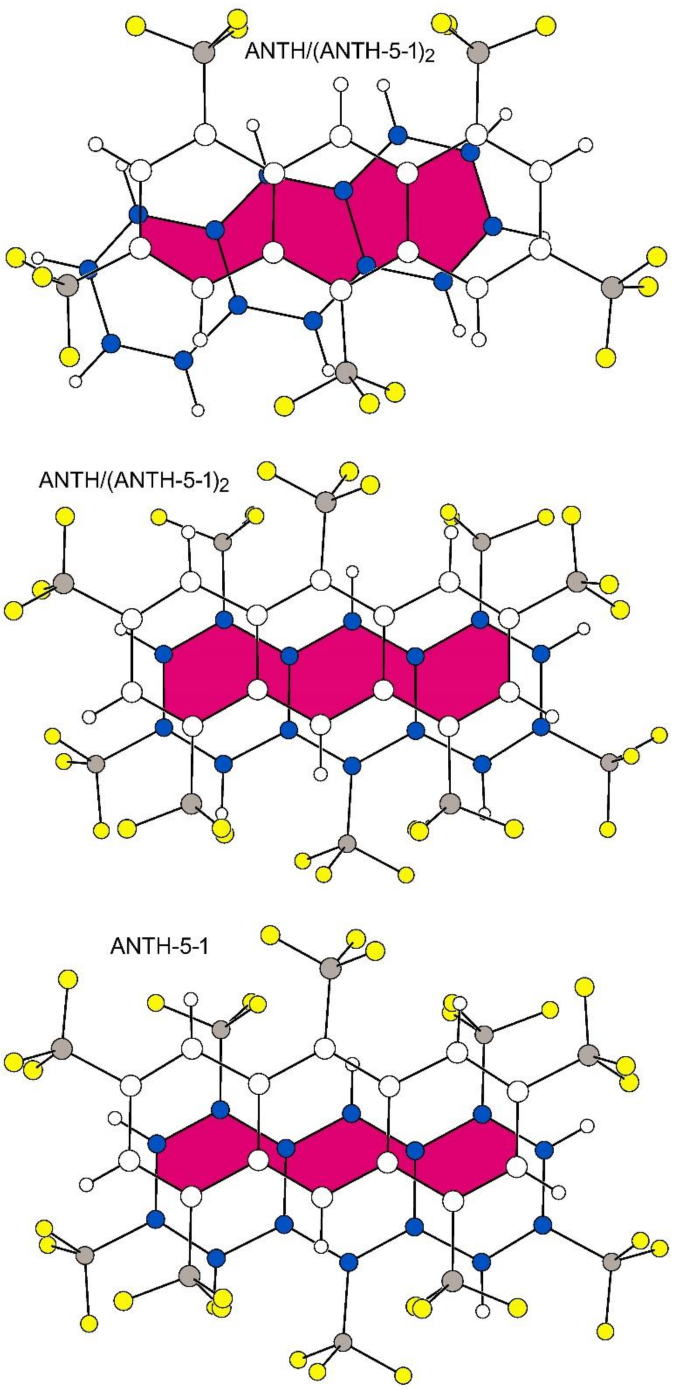

Figure S21. Parallel projection drawings of the π–π overlap (shaded magenta) of neighboring pairs of ANTH and ANTH-5-1 molecules (top) and two ANTH-5-1 molecules (middle) in the structure of ANTH/(ANTH-5-1)$_2$ and two ANTH-5-1 molecules in the structure of ANTH-5-1. In all drawings F atoms are shaded yellow and ANTH-5-1 C(sp^3) atoms are shaded gray. In the top drawing the ANTH C(sp^2) atoms are shaded blue and the least squares plane of the ANTH C(sp^2) atoms is in the plane of the page. In the middle and bottom drawings the C(sp^2) atoms of the lower ANTH-5-1 molecule are shaded blue and the least squares planes of the lower ANTH-5-1 C(sp^2) atoms are in the plane of the page.

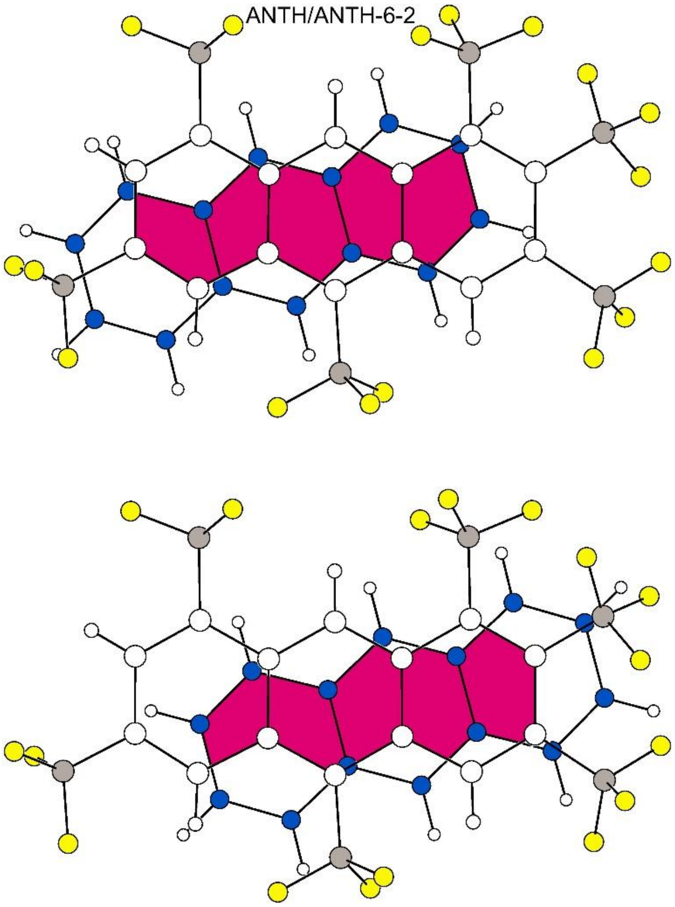

Figure S22. Parallel projection drawings of the π–π overlap (shaded magenta) of neighboring pairs of ANTH and ANTH-6-2 molecules in the structure of ANTH/ANTH-6-2. In all drawings F atoms are shaded yellow, ANTH-6-2 C(sp^3) atoms are shaded gray, ANTH C(sp^2) atoms are shaded blue, and the least squares planes of the ANTH C(sp^2) atoms are in the plane of the page.

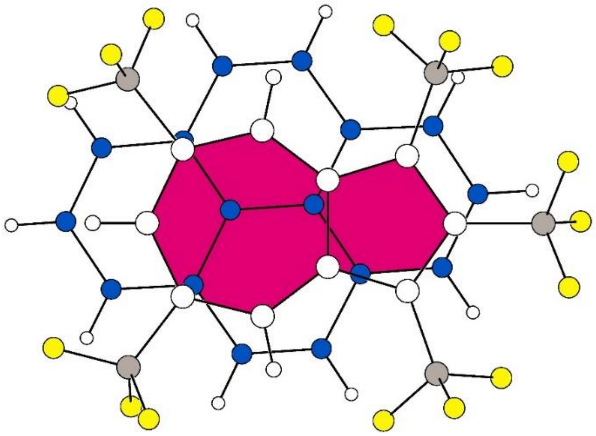

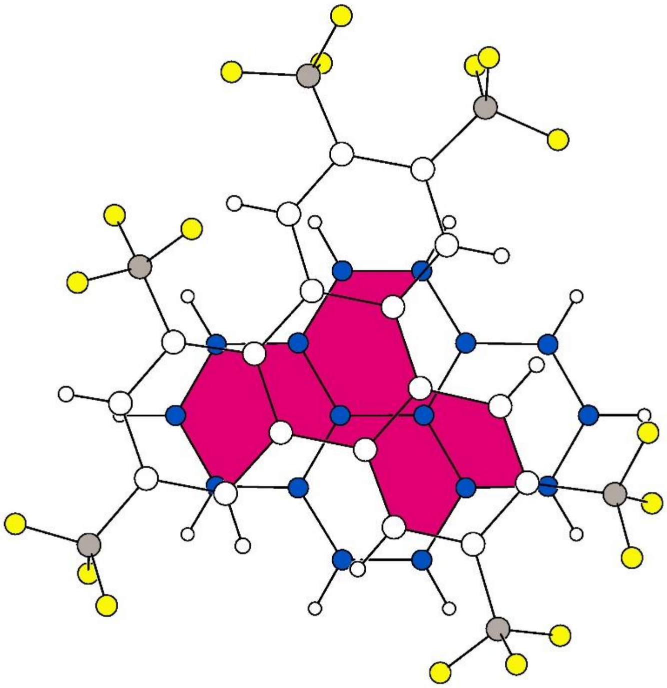

Figure S23. Parallel projection drawings of the π–π overlap (shaded magenta) of neighboring pairs of PYRN and AZUL-5-1 molecules in the structure of PYRN/AZUL-5-1 (top) and PYRN and TRPH-6-1 molecules in the structure of PYRN/(TRPH-6-1)$_2$ (bottom). The F atoms are shaded yellow, AZUL-5-1 and TRPH-6-1 C(sp^3) atoms are shaded gray, PYRN C(sp^2) atoms are shaded blue, and the least squares plane of the PYRN C(sp^2) atoms is in the plane of the page in both drawings.

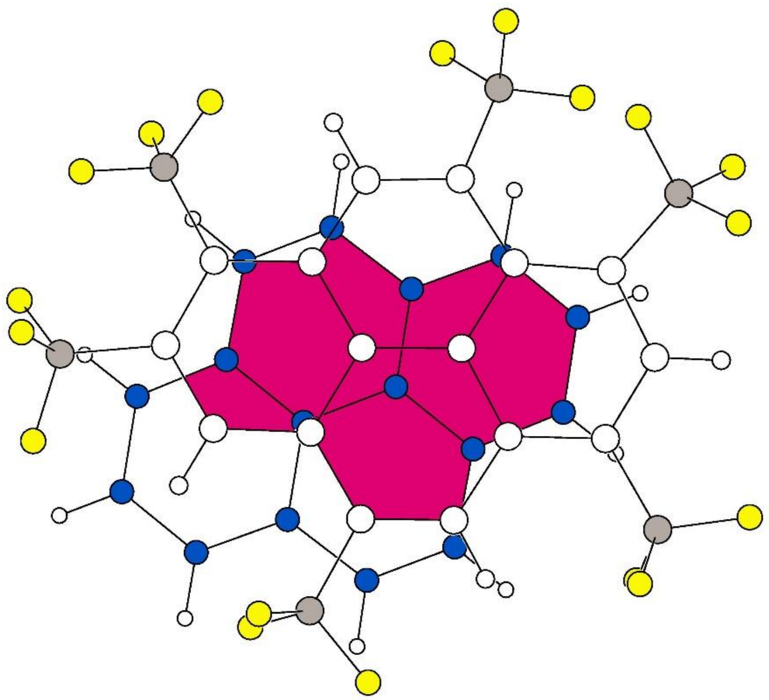

Figure S24. Parallel projection drawing of the π–π overlap (shaded magenta) of one of the four types of neighboring pairs of PYRN and PYRN-6-2 molecules in the structure of PYRN/PYRN-6-2. The F atoms are shaded yellow, PYRN-6-2 C(sp^3) atoms are shaded gray, PYRN C(sp^2) atoms are shaded blue, and the least squares plane of the PYRN C(sp^2) atoms is in the plane of the page.

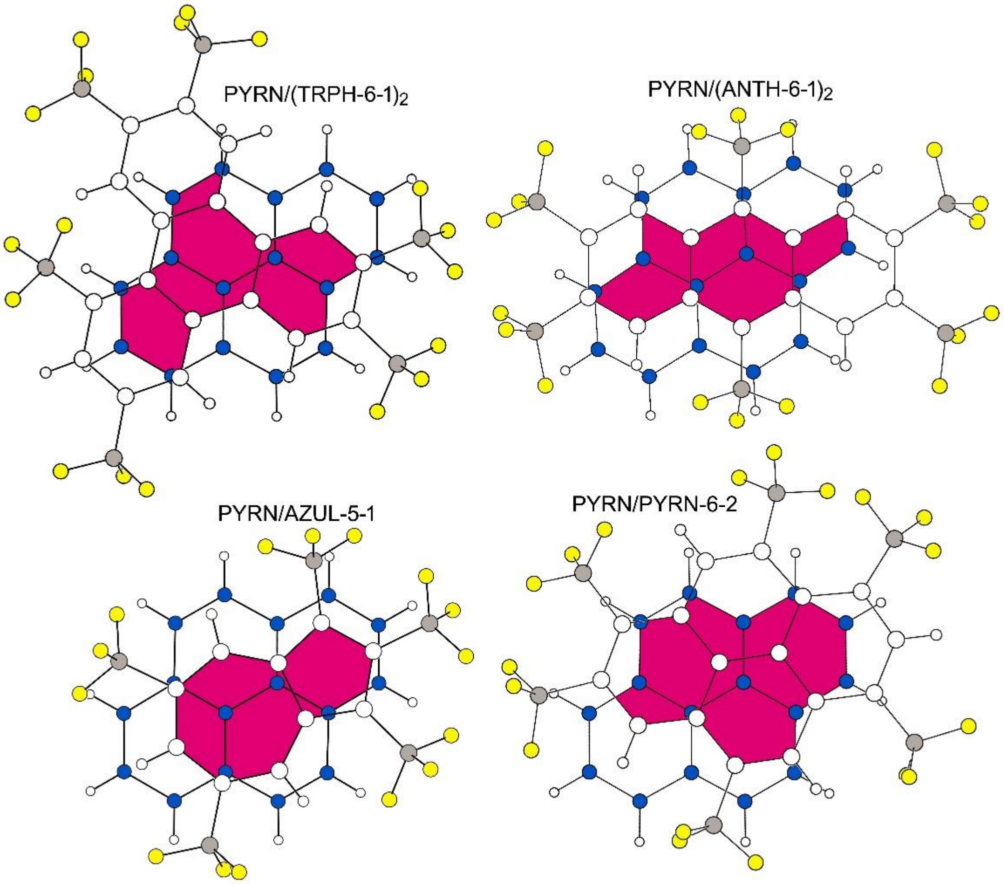

Figure S25. Comparison of parallel projection drawings of the π–π overlap (shaded magenta) in the four PYRN/A$_y$ charge-transfer co-crystals studied in this work. The F atoms are shaded yellow, acceptor C(sp^3) atoms are shaded gray, PYRN C(sp^2) atoms are shaded blue, and the least squares plane of the PYRN C(sp^2) atoms is in the plane of the page in all four drawings. The structures are drawn to scale. The structure of PYRN/(ANTH-6-1)$_2$ was reported in ref. [22].

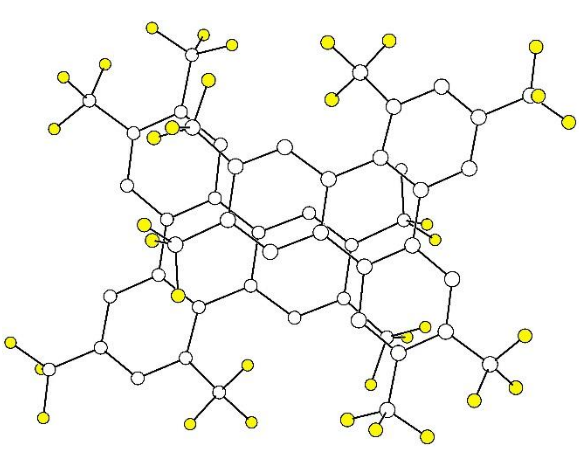

(TRPH-6-1)$_2$ pairs in PYRN/(TRPH-6-1)$_2$

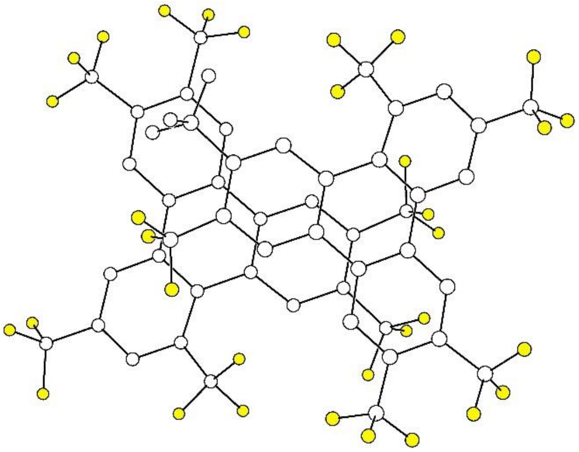

Closer of the two types of (TRPH-6-1)$_2$ pairs in the structure of TRPH-6-1

Figure S26. Comparison of the (TRPH-6-1)$_2$ pair in the structure of PYRN/(TRPH-6-1)$_2$ (left; this work) and the closer and more overlapped of the two types of (TRPH-6-1)$_2$ pairs in the hexagonal arrays of infinite stacks of TRPH-6-1 molecules in the structure of TRPH-6-1 (right; ref. [5]). The H atoms have been omitted for clarity, and F atoms are shaded yellow.

Table S5. Ionization energies (*IE*s), $E_{1/2}(+/0)$ values, and electron affintities (*EA*s) of some PAH donors of interest in this work[a]

compound	abbreviation	*IE*, eV	ref.	$E_{1/2}(+/0)$, V vs. $Fe(Cp)_2^{+/0}$ [b]	*EA*, eV	ref.
naphthalene	NAPH	8.144(1)	[27]	1.16	−0.20(5)	[28]
fluoranthene	FLRA	7.9(1)	[29]	1.07	0.629995	[30]
phenanthrene	PHEN	7.891(1)	[31]	1.12	−0.01(4)	[32]
triphenylene	TRPH	7.87(2)[c]	—	1.17	0.285(8)	[33]
chrysene	CHRY	7.60(1)[c]	—	0.97	0.32(1)	[34]
picene	PICE	7.51(2)[c]	—	0.95	0.542(8)	[33]
anthracene	ANTH	7.439(6)	[31]	0.71	0.53(2)	[35]
benzanthracene	BzANTH	7.45(5)	—	—[d]	0.39(10)	[36]
pyrene	PYRN	7.426(1)	[31]	0.78	0.41(1)	[37]
coronene	CORO	7.26(5)	[38]	0.85	0.47(9)	[39]
perylene	PERY	6.960(1)	[40]	0.47	0.973(5)	[41]
tetracene	TETR	6.97(5)[c]	—	0.39	1.058(5)	[42]
pentacene	PENT	6.63(5)[c]	—	—[d]	1.39(4)	[43]

[a] Unless otherwise indicated, only the most recent, most precise, and/or most reliable entries in the NIST WebBook (ref. [44]) are included in this table. [b] Polarographic $E_{1/2}(+/0)$ values vs. SCE in CH_3CN containing 2 M $NaClO_4$ (ref. [45]); $E_{1/2}(+/0)$ for $Fe(Cp)_2$ is 0.38 V vs. SCE in CH_3CN (ref. [46]). [c] This is the "evaluated" *IE* value listed in the NIST WebBook. [d] $E_{1/2}(+/0)$ values for BzANTH and PENT were not reported.

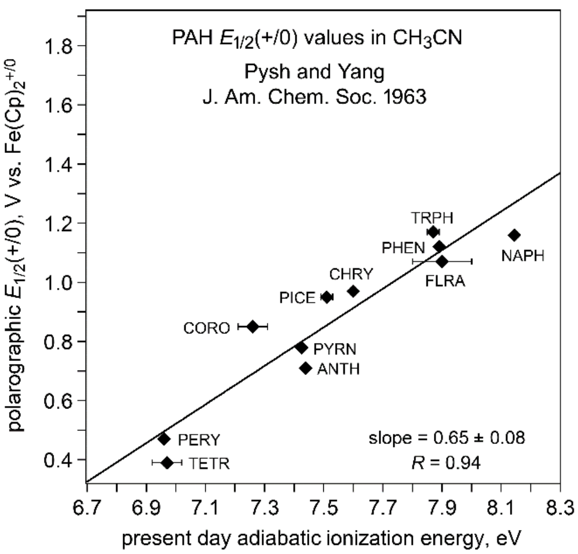

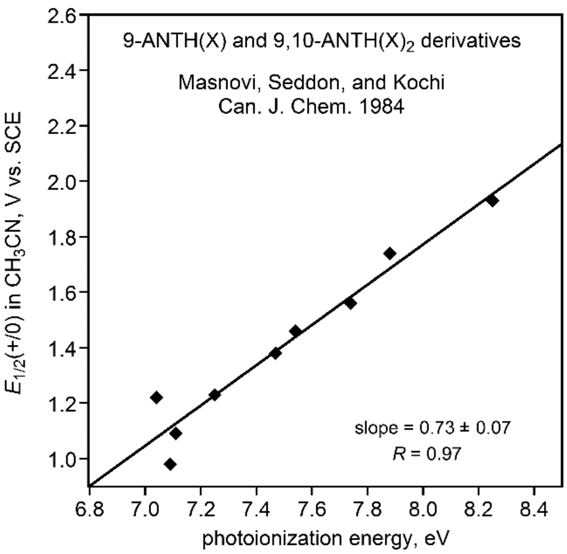

Figure S27. Left: Plot of the most recent and most precise PAH adiabatic *IE*s vs. polarographic $E_{1/2}$(+/0) values published in ref. [45]. This plot is also part of Figure 11 in the main text. Right: Gas phase photoionization energy vs. cyclic voltammetric $E_{1/2}$(+/0) values for anthracene (ANTH) derivatives with Me, Cl, Br, NO_2, OMe, CHO, and/or Ph substituents on the 9 and 10 positions (ref. [47]). Note that the lengths and ranges of values on both axes are equal (i.e., these are square plots), to show visually and unequivocally that the slopes are less than unity.

Table S6. Electron affinities (*EA*s) and $E_{1/2}(0/-)$ values of electron acceptors of interest in this work[a]

compound	abbreviation	*EA*, eV	$E_{1/2}(0/-)$, V vs. $Fe(Cp)_2^{+/0}$ [b]	ref.
benzoquinone	BQ	1.91(6)	—	[48]
tetrachlorophthalic anhydride	TCPA	1.95(9)	—	[49]
1,2,4,5-tetracyanobenzene[c]	TCNB	2.2(2)	—	[50]
4-nitrobenzodifuroxan	NBDF	2.45 (estimate)[d]	—	[23]
tetrafluorobenzoquinone	F_4BQ	2.70(6)	—	[48]
tetrachlorobenzoquinone[e]	Cl_4BQ	2.78(6)	—	[48]
tetracyanoquinodimethane	TCNQ	3.383(1)	—	[51]
tetracyanoethylene	TCNE	3.16(2)	—	[52]
2,3,5,6-tetrafluoro-TCNQ	F_4TCNQ	3.2[f,g]	—	[53]
1,3,6,8,10-ANTH$(CF_3)_5$	ANTH-5-1	2.40(2)	−1.24	[5]
2,3,6,7,9,10-ANTH$(CF_3)_6$	ANTH-6-1	2.81(2)	−1.01	[5]
1,2,3,6,8,10-ANTH$(CF_3)_6$	ANTH-6-2	2.68(2)	−1.05	this work
1,3,5,7-AZUL$(CF_3)_4$	AZUL-4-1	2.485(10)	−1.05	[6]
1,2,3,5-AZUL$(CF_3)_4$	AZUL-4-2	2.495(10)	—	[6]
1,2,3,5,7-AZUL$(CF_3)_5$	AZUL-5-1	2.815(15)	−0.73	[5]
1,3,5,7-NAPH$(CF_3)_4$	NAPH-4-1	1.795(20)	−1.62	[16]
1,4,7,10-PERY$(CF_3)_4$	PERY-4-1	2.20(2)	−1.30	[5]
1,3,6,8,10-PERY$(CF_3)_5$	PERY-5-1	2.46(2)	−1.19	[5]
1,3,6,8,10-PERY$(CF_3)_6$	PERY-6-1	2.72(2)	−1.01	[5]
1,3,6,7,9-PHEN$(CF_3)_5$	PHEN-5-1	1.95(1)	−1.71	[5]
2,4,6-PHNZ$(CF_3)_3$	PHNZ-3-1	2.37(2)	−1.07	[21]
1,4,6-PHNZ$(CF_3)_3$	PHNZ-3-2	2.30(2)	−1.12	[21]
1,4,6,8-PHNZ$(CF_3)_4$	PHNZ-4-1	2.65(1)	−0.92	[21]
1,4,6,9-PHNZ$(CF_3)_4$	PHNZ-4-2	2.60(1)	−0.97	[21]
1,2,4,6,9-PHNZ$(CF_3)_5$	PHNZ-5-1	2.93(1)	−0.71	[21]
1,3,4,6,8-PYRN$(CF_3)_5$	PYRN-5-1	2.44(2)	−1.25	[5]
1,3,4,6,9-PYRN$(CF_3)_5$	PYRN-5-2	2.38(2)	−1.27	[5]
1,3,4,6,8,9-PYRN$(CF_3)_6$	PYRN-6-1	2.71(2)	−1.01	[5]
1,2,4,6,8,9-PYRN$(CF_3)_6$	PYRN-6-2	2.6–2.7[h]	−1.05	this work
1,3,6,7,10,11-TRPH$(CF_3)_6$	TRPH-6-1	2.11(2)	−1.73	[5]

[a] Unless otherwise indicated, only the most recent, most precise, and/or most reliable entries in the NIST WebBook (ref. [44]) are included in this table. [b] $E_{1/2}(0/-)$ values were measured by cyclic voltammetry in dimethoxyethane containing 0.1 M $N(n\text{-}Bu)_4^+ClO_4^-$. [c] Commonly known as pyromellitonitrile. [d] This estimated value may be incorrect for two reasons: (i) it was based on the erroneous assumption of a 1:1 correlation between gas-phase *EA* and solution-phase CT complex $E(\lambda_{max})$; and (ii) it was based on an incorrect value of *EA* for TCNE, 2.88 eV, instead of the correct value given in the table above, 3.16(2) eV (see the Supporting Information for ref. [23]). [e] Commonly known as chloranil. [f] No uncertainty was reported. [g] Not listed in NIST WebBook. [h] Estimate based on the *EA* of PYRN-6-1 and the $E_{1/2}(0/-)$ values for both isomers and the *EA*s and $E_{1/2}(0/-)$ values for other PAH$(CF_3)_n$ isomers.

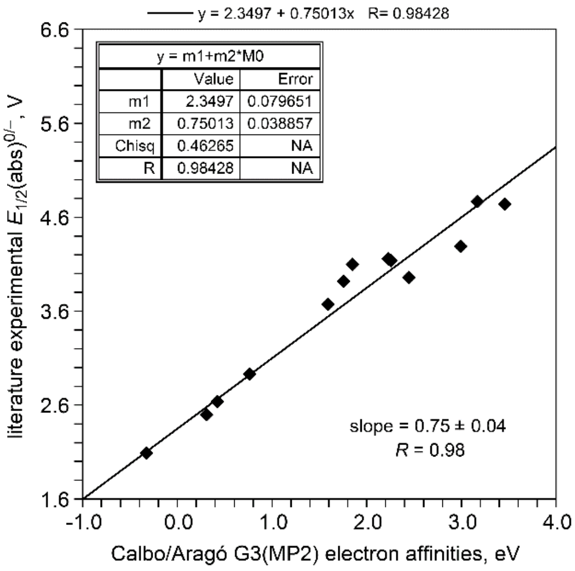

Figure S28. Electrochemical $E_{1/2}(0/-)$ values vs. G3(MP2) predicted electron affinities for aromatic compounds and several strong electron acceptors, including C_6H_6, NAPH, ANTH, PYRN, PERY, benzoquinone, 1,2,4,5-$C_6H_2(CN)_4$, TCNE, and TCNQ, among others (ref. [54]). Note that the lengths and ranges of values on both axes are equal (i.e., this is a square plot), to show visually and unequivocally that the slope is less than unity).

Table S7. Equilibrium constants for D/A CT complexes with D = anthracene (ANTH)[a]

D/A CT complex	solvent	K_{eq}, M^{-1}	K_{eq} ref.	acceptor *EA*, eV[b]
ANTH/ANTH-5-1	CH_2Cl_2	1.7(1)	this work	2.40(2)
ANTH/ANTH-6-1	CH_2Cl_2	2.8(1)	this work	2.81(2)
ANTH/chloranil	CH_2Cl_2	1.0	[55]	2.78(6)
ANTH/chloranil	$CHCl_3$	1.7	[55]	2.78(6)
ANTH/chloranil	CCl_4	3.1	[55]	2.78(6)
ANTH/NBDF	$CHCl_3$	5.5(3)	[23]	2.45[c]
ANTH/TCPA	CCl_4	8.75	[56-57]	1.95(9)
ANTH/TCNQ	THF	5.2(8)	[58]	3.383(1)

[a] Abbreviations: NBDF = 4-nitrobenzodifuroxan; TCPA = tetrachlorophthalic anhydride; chloranil = tetrachlorobenzoquinone. [b] See Table S6 for *EA* references. [c] This estimated value may be incorrect. See footnote d in Table S6.

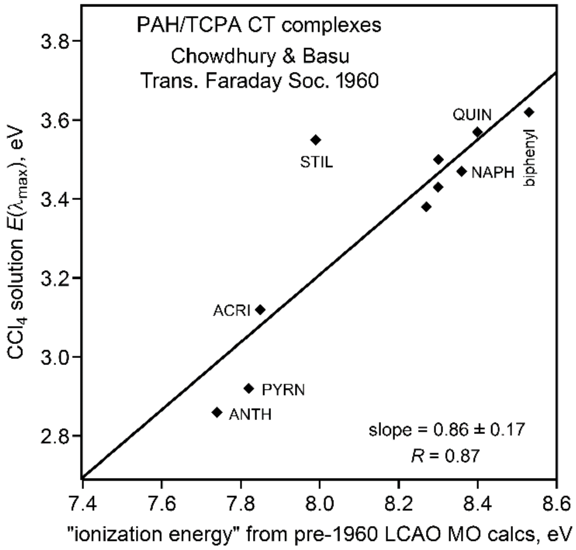

Figure S29. Left: $E(\lambda_{max})$ values for CT complexes of the electron acceptor tetrachlorophthalic anhydride (TCPA) with PAHs and other aromatic hydrocarbon donors vs. "ionization energy" determined by pre-1960 LCAO MO calculations. The plot was made for this work from the data reported in ref. [57]. In that work, Chowdhury and Basu claimed that the slope of their plot (which was not shown) was 1.0 eV/eV ("unit slope"), but in fact the slope is actually 0.86 ± 0.17 eV/eV. Right: Plot using the same set of $E(\lambda_{max})$ values and the most recent and most precise adiabatic *IE*s for the donors. The slope is 0.64 ± 0.13 eV/eV, similar to the slopes of similar plots in the literature (see text) and in this work (see Figure 12). Note that the lengths and ranges of values on both axes are equal (i.e., these are square plots), to show visually and unequivocally that the slopes are less than unity).

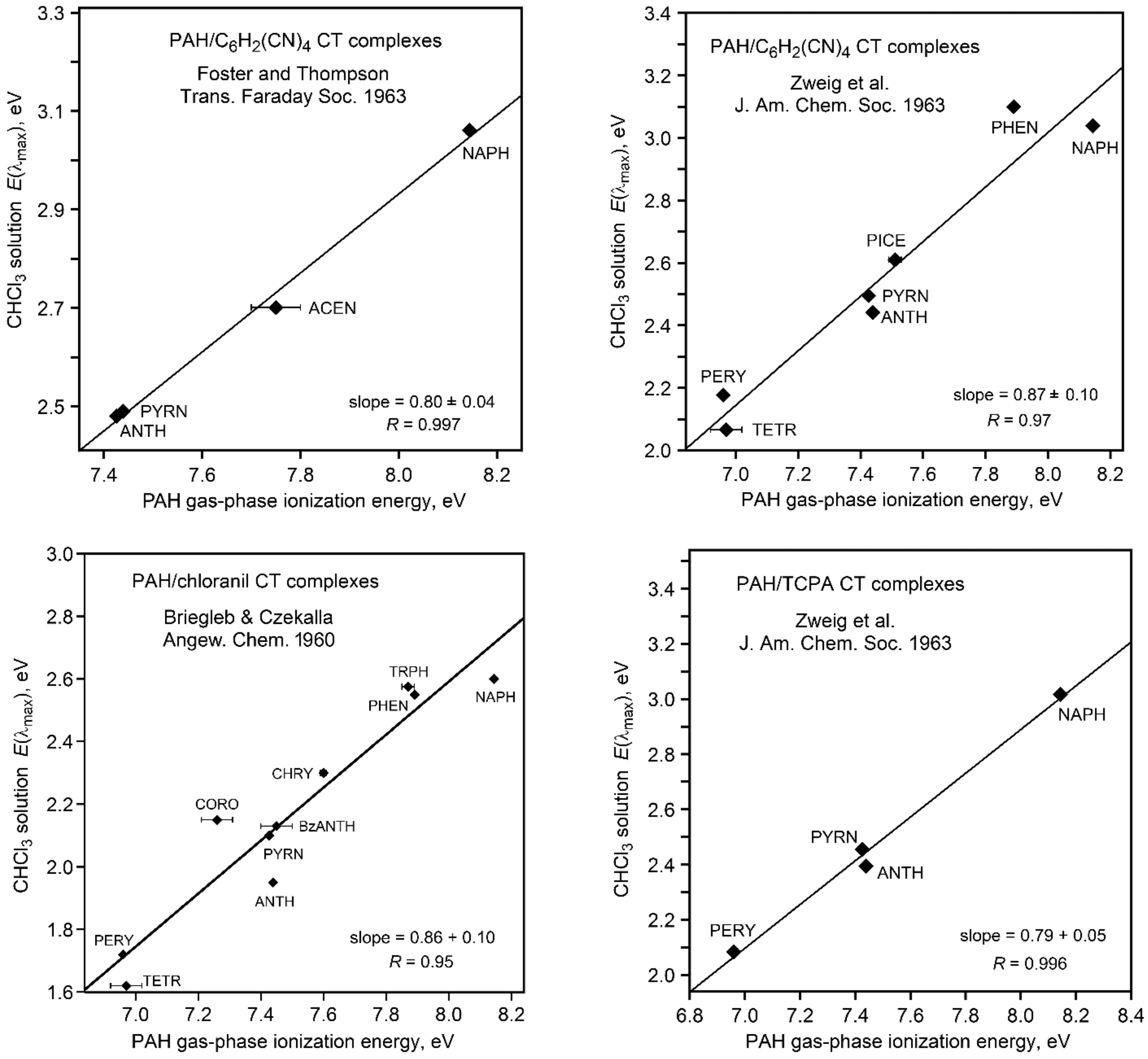

Figure S30. Charge-transfer complex $E(\lambda_{max})$ vs. PAH ionization energy plots prepared for this work from $E(\lambda_{max})$ data published in refs. [59] (upper left), [60] (lower left), and [61] (upper and lower right). Only the most recent and most precise *IE*s from the NIST WebBook were used. Note that the lengths and ranges of values on both axes are equal (i.e., these are square plots), to show visually and unequivocally that the slopes are less than unity).

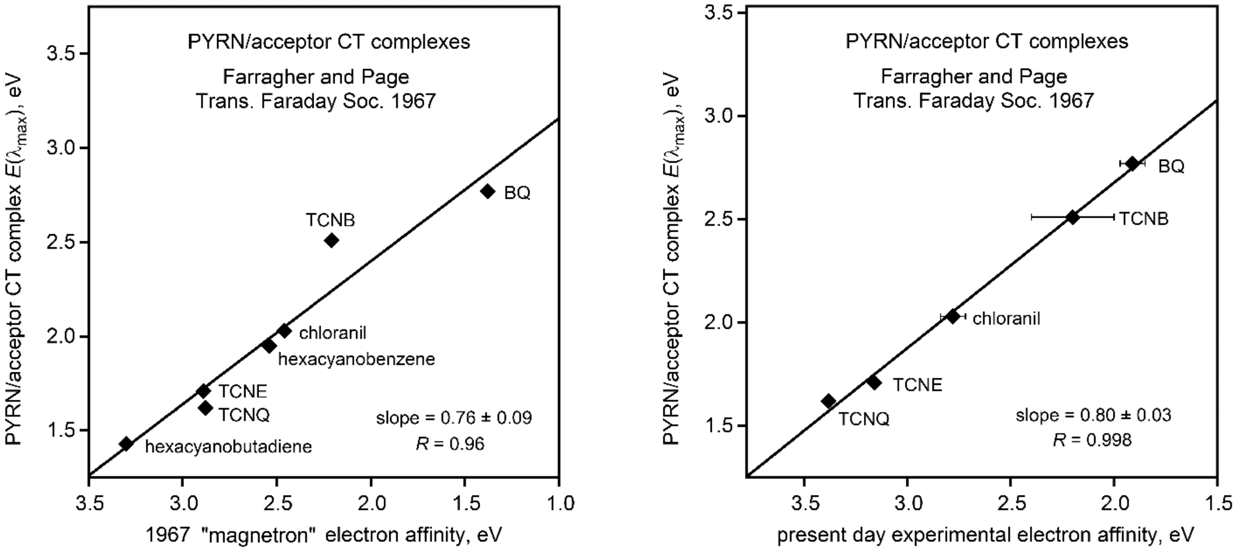

Figure S31. Plots of $E(\lambda_{max})$ vs. acceptor *EA*s for CT complexes of PYRN with various electron acceptors reported in ref. [50] (the solvent was not specified). Both plots were prepared for this work (there is no corresponding plot in ref. [50]). The plot on the left was prepared using the *EA*s in the original paper, which were determined by the magnetron method and reported in the same paper as the $E(\lambda_{max})$ values. The plot on the right was made using the most recent and most precise adiabatic *EA*s, which are listed in Table S6. Note that the lengths and ranges of values on both axes are equal (i.e., these are square plots), to show visually and unequivocally that the slopes are less than unity. Using the linear least-squares fit to the data (in eV) for the plot on the right (i.e., $E(\lambda_{max}) = (4.28 \pm 0.09) - (0.80 \pm 0.03)(EA)$), the interpolated *EA* of hexacyanobenzene is 2.9(1) eV, which is significantly smaller than the 3.53 eV DFT predicted value reported by Schaefer et al. in ref. [62].

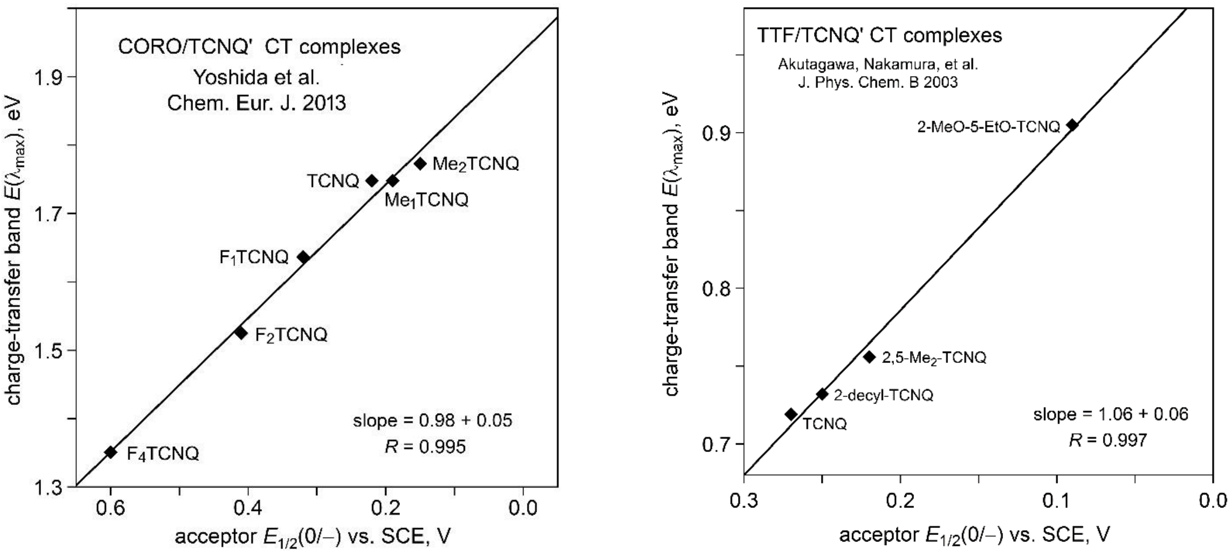

Figure S32. Plots made for this work of acceptor $E_{1/2}(0/-)$ vs. $E(\lambda_{max})$ values for CT complexes of CORO with various TCNQ derivatives reported in ref. [63] (left) and CT complexes of TTF with TCNQ derivatives reported in ref. [64] (right). Note that the lengths and ranges of values on both axes are equal (i.e., these are square plots), to show visually and unequivocally that the slopes of the plots are close to unity. Note also that the units for the slopes are eV/V. These 1:1 correlations were not obvious in the plots shown in refs. [63] and [64] because the the $E_{1/2}(0/-)$ values were plotted against the frequency of the charge-transfer band in cm^{-1}, not against the energy of the charge transfer band in eV.

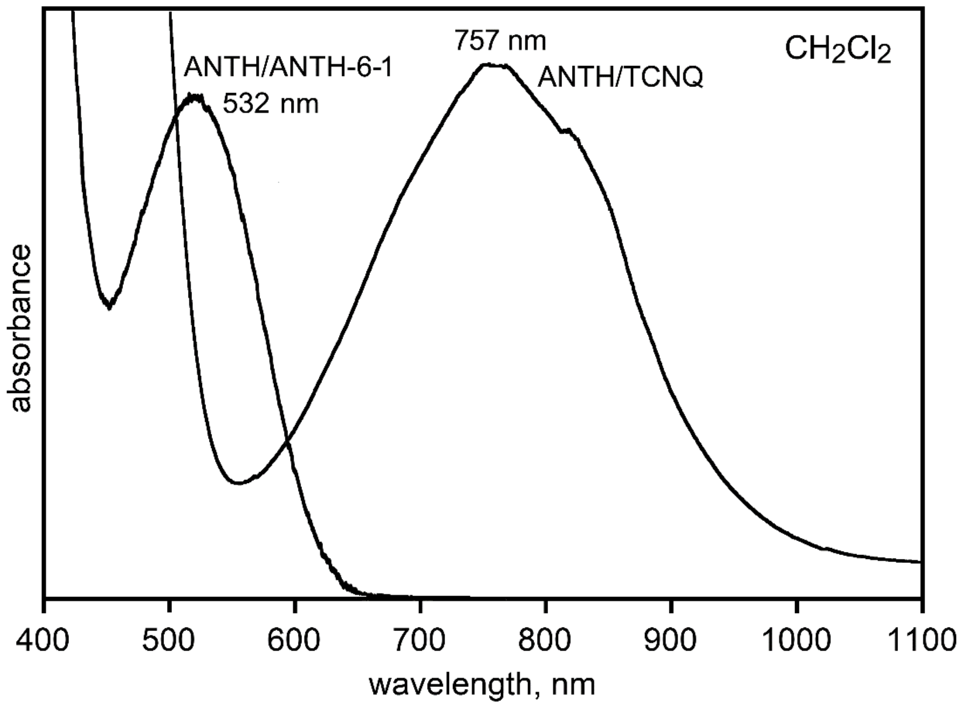

Figure S33. Electronic spectra of the ANTH/ANTH-6-1 and ANTH/TCNQ CT complexes in CH_2Cl_2. The lowest-energy CT band may be higher than 800 nm. Charge-transfer complexes of ANTH with other acceptors (e.g., TCNE) show an overlapped "doublet" set of CT bands (ref. [24]). The λ_{max} value for ANTH/TCNQ in THF solution is 795 nm (ref. [58]).

Table S8. Donor/acceptor interplanar distances defined in two different ways[a]

Co-crystal structure	range of A→D(LSP) OOPs; average A→D(LSP) OOP, Å[b]	half the perpendicular distance between successive donor LSPs or successive acceptor LSPs, Å
ANTH/(ANTH-5-1)$_2$	3.43–3.54; 3.49	3.51
ANTH/ANTH-6-1	3.47–3.66; 3.55	3.48
ANTH/ANTH-6-2	3.45–3.69; 3.52<br>3.49-3.62; 3.54	3.49
(CORO)$_2$/ANTH-6-1	3.43–3.75; 3.61	3.60
PERY/ANTH-6-1	3.48–3.66; 3.55	3.54
PYRN/AZUL-5-1	3.58–3.65; 3.61	3.60
PYRN/(ANTH-6-1)$_2$	3.36–3.73; 3.56	—[c]
PYRN/PYRN-6-2	3.41–3.59; 3.50	—[d]
	3.47–3.71; 3.58	—[d]
PYRN/(TRPH-6-1)$_2$	3.37–3.73; 3.54	3.56[e]
ANTH/NAPH(F)$_8$	3.31–3.40; 3.35	3.38

[a] All values from this work except for ANTH/NAPH(F)$_8$ (ref. [65]), PYRN/AZUL5-1 (ref. [6]), and PYRN/(ANTH-6-1)$_2$ (ref. [22]); LSP = least-squares plane of C(sp^2) atoms; OOP = perpendicular out-of-plane distance (i.e., from the least squares plane of the donor C atoms). [b] These are the values listed in Table 4 in the main text. [c] Not applicable for this structure. [d] Not readily defined due to the complexity of the structure (two unique PYRN donors and two unique PYRN-6-2 acceptors). [e] This is the OOP of the centroid of the nearly-planar hexagon on TRPH-6-1 that maximally overlaps with the π system of PYRN (i.e., the perpendicular distance of that centroid from the LSP of the PYRN C(sp^2) atoms).

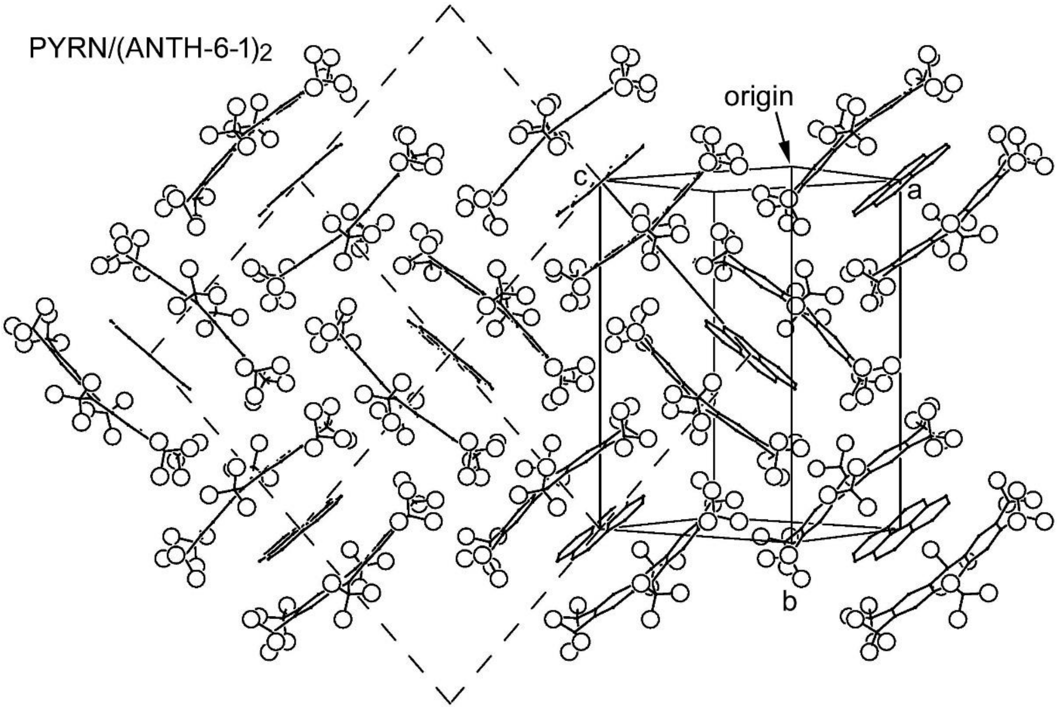

Figure S34. The structure of PYRN/(ANTH-6-1)$_2$, recently published in ref. [22] (H atoms omitted for clarity; the larger spheres are F atoms; a unit cell outline is shown with solid lines). The discrete {ANTH-6-1/PYRN/ANTH-6-1} ({A/D/A}) complexes of nearly-parallel molecules are packed in herringbone sandwich layers in crystallographic 101 planes such that the PYRN centroids (⊙) are rigorously co-planar. The {A/D/A} complexes in each layer form a pseudo-square net depicted with dashed lines: all ⊙···⊙ distances are 12.37 Å and the ⊙···⊙···⊙ angles that define the nearly-square rhombus are 81.4 and 98.6°. The PYRN least-square planes (LSPs) are tilted 84.6° from the 101 plane; the PYRN LSPs in neighboring {A/D/A} sandwiches in the 101 plane are tilted 81.4° relative to one another. The layers of {A/D/A} complexes above and below the layer shown are displaced by *b*/2 (the figure is oriented so that the *b* axis is the long axis of the page).

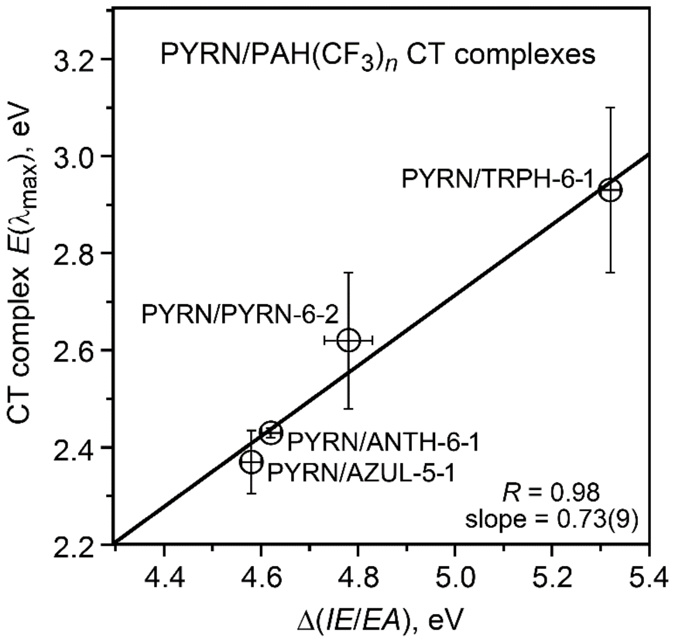

Figure S35. Plot of $E(\lambda_{max})$ vs. $\Delta(IE/EA)$ for the four PYRN/PAH$(CF_3)_n$ CT complexes studied in this work ($\Delta(IE/EA) = IE$(PYRN) − EA(PAH$(CF_3)_n$)). See Table 1 for details. Note that the lengths and ranges of values on both axes are equal (i.e., this is a square plot), to show visually and unequivocally that the slope of the linear least-squares line is significantly less than unity.

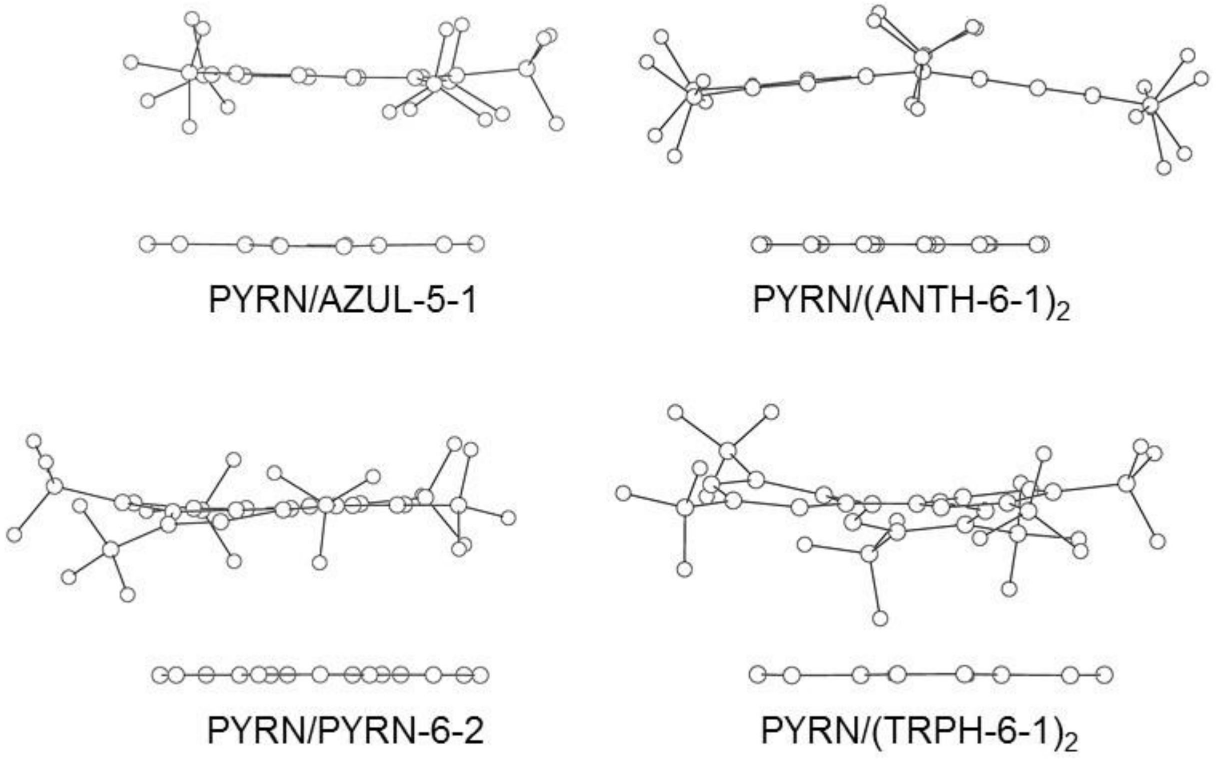

Figure S36. Side views of the structures of PYRN/AZUL-5-1 (ref. [6]), PYRN/(ANTH-6-1)$_2$ (ref. [22]), PYRN/PYRN-6-2, and PYRN/(TRPH)$_2$, showing the non-planarity of the PAH(CF_3)$_6$ acceptors in the latter three structures. The drawings are approximately, but not exactly, to scale.

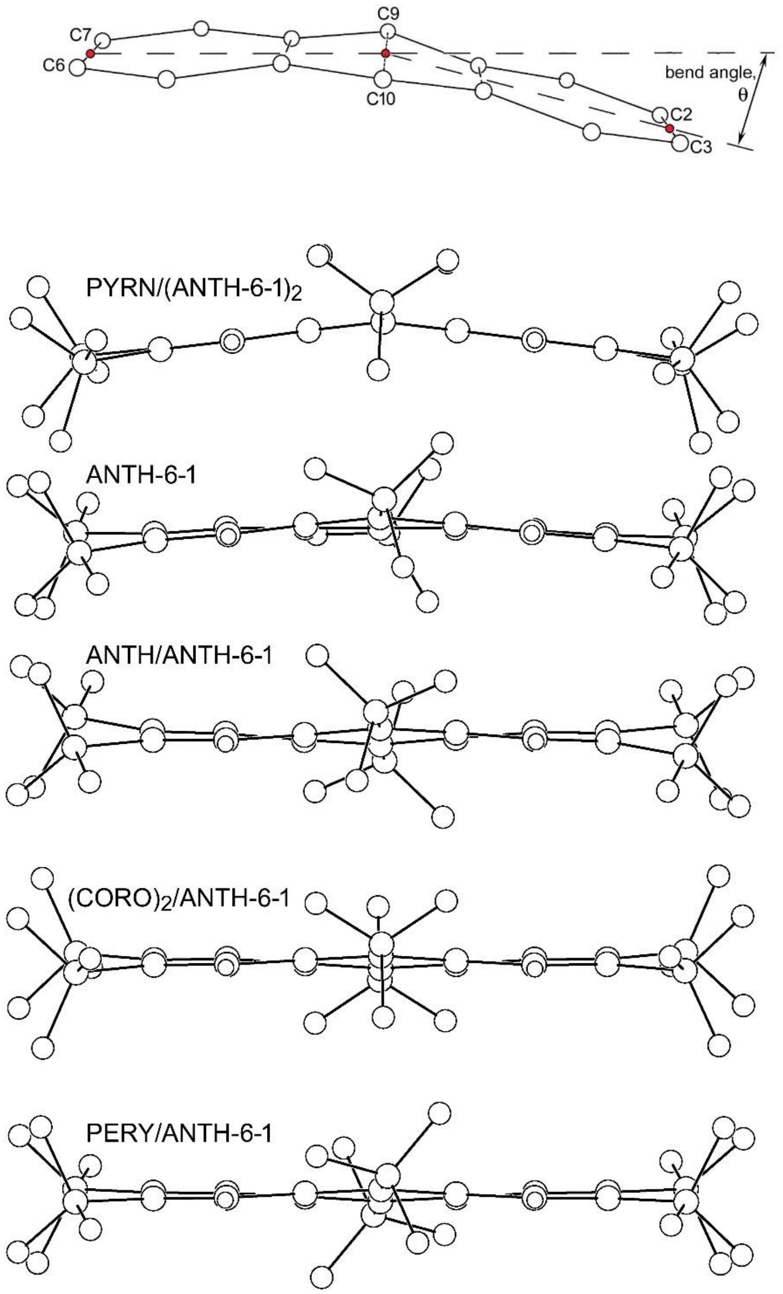

Figure S37. Definition of the bend angle θ (the red dots are centroids) and flat projections perpendicular to the least-squares planes of the ANTH-6-1 aromatic cores in the X-ray structures of (top to bottom) PYRN/(ANTH-6-1)$_2$ (ref. [22]), ANTH-6-1 (ref. [22]), ANTH/ANTH-6-1, (CORO)$_2$/ANTH-6-1, and PERY/ANTH-6-1. The bend angles θ are (top to bottom) 13.4°, 7.4°, 0.0°, 0.0°, and 0.0°.

DFT optimized PYRN/ANTH-6-1	$PYRN/(ANTH\text{-}6\text{-}1)_2$ X-ray structure

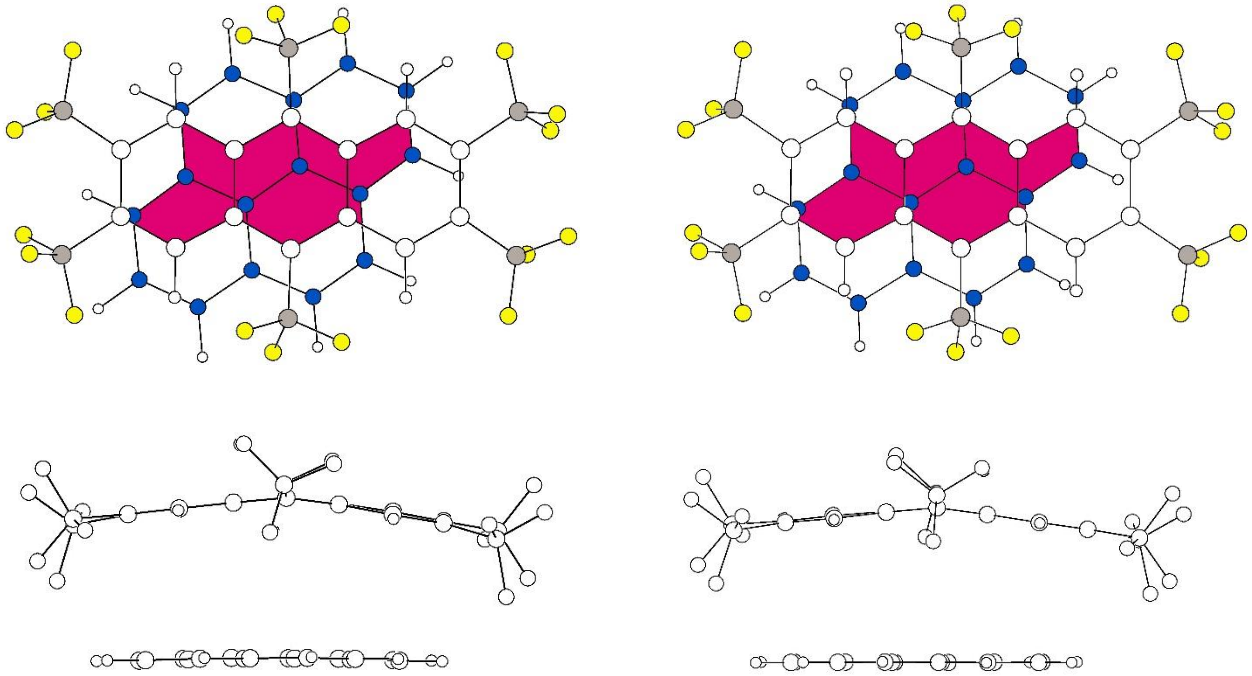

ANTH-6-1 bend angle θ = 15.1°	ANTH-6-1 bend angle θ = 13.4°
central CF_3 groups eclipsed	central CF_3 groups staggered
A→D(LSP) π–π OOPs 3.39–3.73 (ave.3.57 Å)	A→D(LSP) π–π OOPs 3.36–3.73 (ave. 3.56 Å)
LSP tilt angle = 1.4°	LSP tilt angle = 1.6°
rotation of D/A major axes = 35.3°	rotation of D/A major axes = 33.0°

Figure S38. Comparison of the DFT optimized and X-ray structures of the PYRN/ANTH-6-1 charge-transfer complexes. The ANTH-6-1 bend angle θ is defined in Figure S37. The abbreviation A→D(LSP) π–π OOPs denotes perpendicular displacements from the least-squares plane of the PYRN C(sp^2) atoms to the ten ANTH-6-1 C(sp^2) atoms that overlap the PYRN π system. The LSP tilt angle is the dihedral angle between the least-squares planes of the PYRN and the ANTH-6-1 C(sp^2) atoms. The X-ray structure of $PYRN(ANTH\text{-}6\text{-}1)_2$ was published in ref. [22].

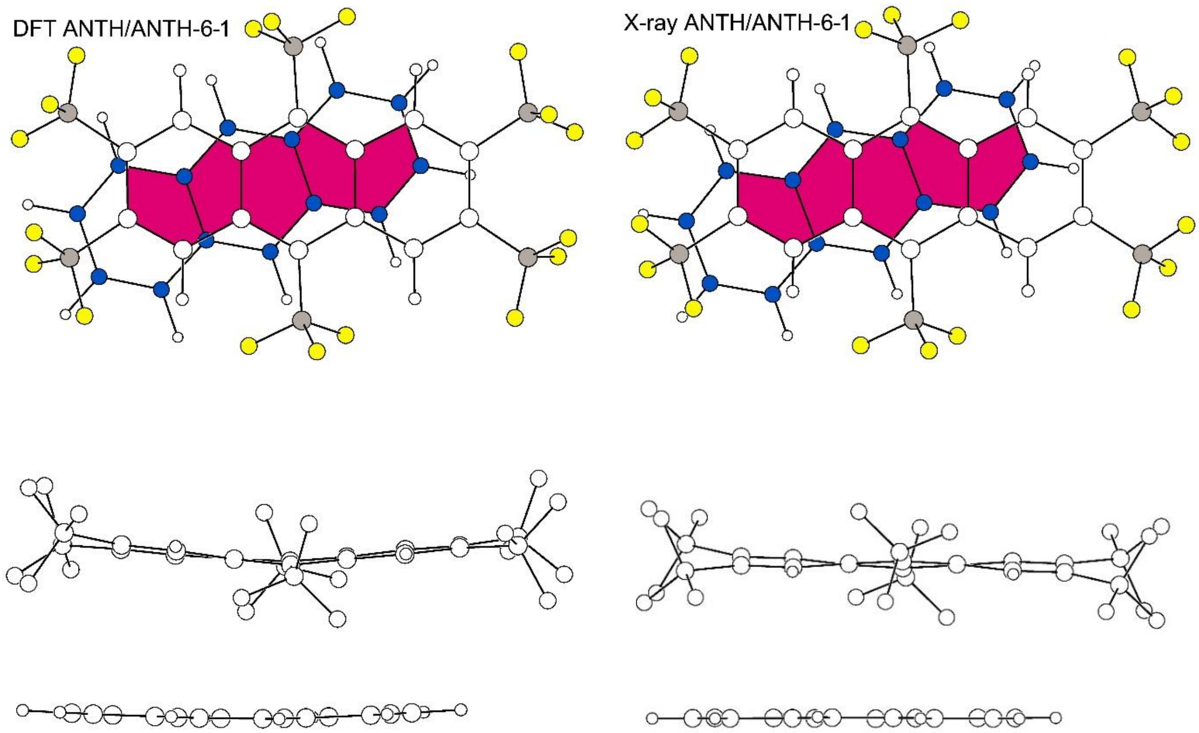

ANTH-6-1 bend angle θ = 10.9°	ANTH-6-1 bend angle θ = 0.0°
central CF_3 groups partially eclipsed	central CF_3 groups staggered
A→D(LSP) π–π OOPs 3.39–3.70 (ave.3.50 Å)	A→D(LSP) π–π OOPs 3.48–3.66 (ave. 3.55 Å)
LSP tilt angle = 0.4°	LSP tilt angle = 2.4°
rotation of D/A major axes = 19.0°	rotation of D/A major axes = 20.7°

Figure S39. Comparison of the DFT optimized and X-ray structures of the ANTH/ANTH-6-1 charge-transfer complexes. The ANTH-6-1 bend angle θ is defined in Figure S37. The abbreviation The abbreviation A→D(LSP) π–π OOPs denotes denotes the perpendicular displacements from the least-squares plane of the ANTH donor C(sp^2) atoms to the ANTH-6-1 C(sp^2) atoms that overlap the ANTH donor π system (five in the DFT complex, six in the X-ray structure). The LSP tilt angle is the dihedral angle between the least-squares planes of the ANTH donor and the ANTH-6-1 acceptor C(sp^2) atoms.

DFT optimized CORO/ANTH-6-1 | $(CORO)_2/(ANTH\text{-}6\text{-}1)_2$ X-ray structure

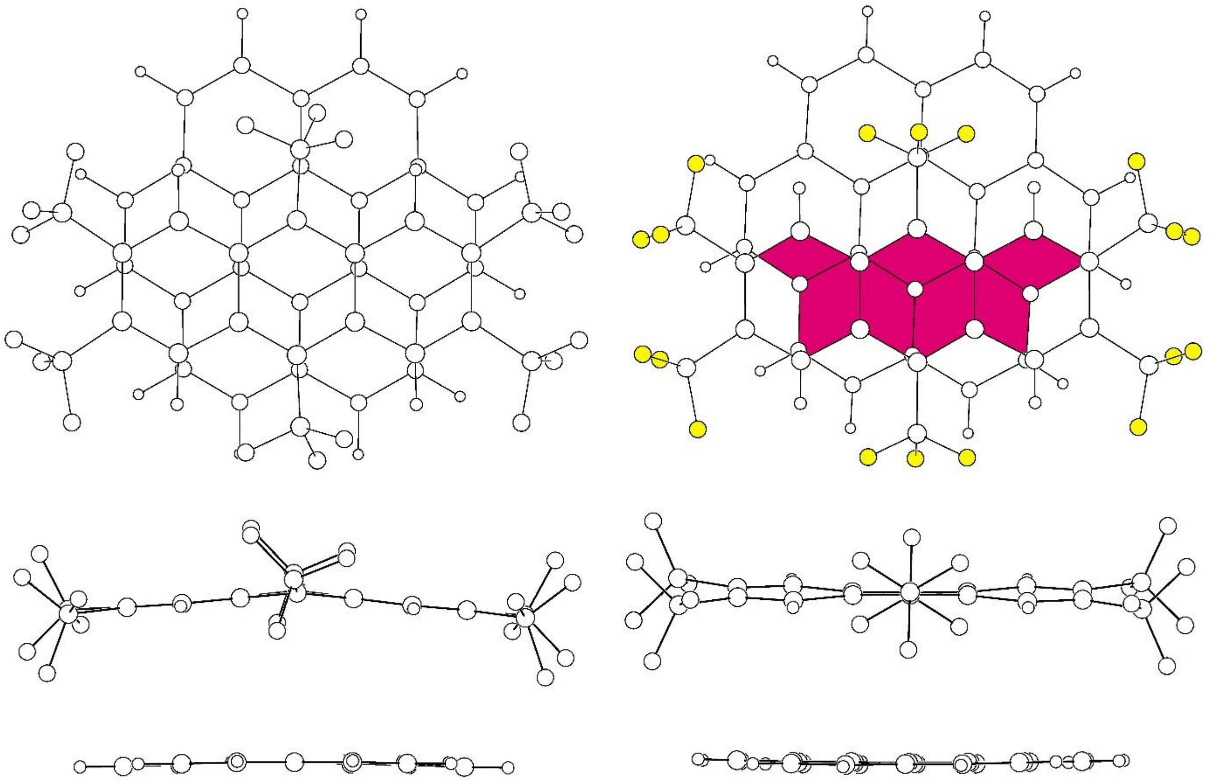

ANTH-6-1 bend angle θ = 11.4°
central CF_3 groups eclipsed
A→D(LSP) π–π OOPs 3.29–3.73 (ave.3.52 Å)
LSP tilt angle = 0.8°
rotation of D/A major axes = 0.4°

ANTH-6-1 bend angle θ = 0.0°
central CF_3 groups staggered
A→D(LSP) π–π OOPs 3.43–3.75 (ave. 3.61 Å)
LSP tilt angle = 4.9°
rotation of D/A major axes = 2.6°

Figure S40. Comparison of the DFT optimized and X-ray structures of the CORO/ANTH-6-1 charge-transfer complexes. The ANTH-6-1 bend angle θ is defined in Figure S37. The abbreviation A→D(LSP) π–π OOPs denotes the perpendicular displacements from the least-squares plane of the CORO donor $C(sp^2)$ atoms to the eleven (11) ANTH-6-1 $C(sp^2)$ atoms that overlap the CORO donor π system. The LSP tilt angle is the dihedral angle between the least-squares planes of the CORO donor and the ANTH-6-1 acceptor $C(sp^2)$ atoms.

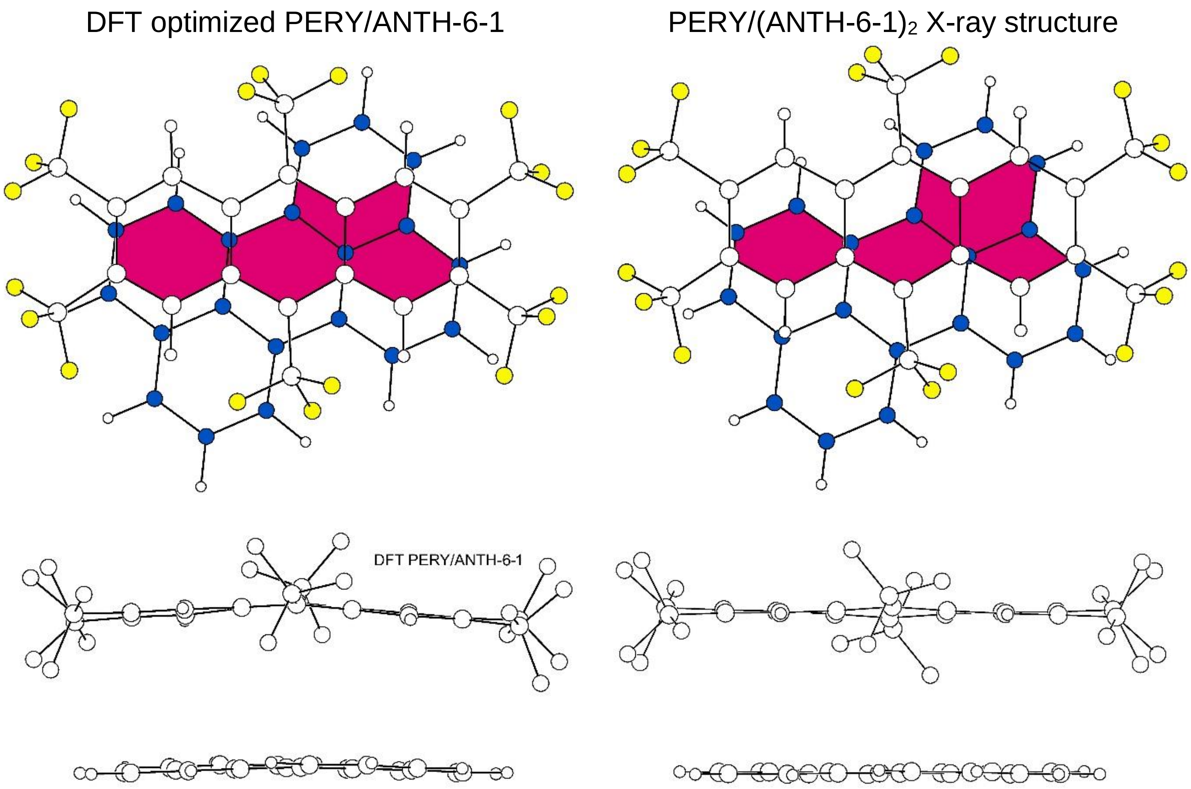

ANTH-6-1 bend angle θ = 9.0°
central CF_3 groups partially eclipsed
A→D(LSP) π–π OOPs 3.34–3.67 (ave.3.47 Å)
LSP tilt angle = 0.8°
rotation of D/A major axes = 24.0°

ANTH-6-1 bend angle θ = 0.0°
central CF_3 groups staggered
A→D(LSP) π–π OOPs 3.48–3.66 (ave. 3.55 Å)
LSP tilt angle = 1.0°
rotation of D/A major axes = 23.2°

Figure S41. Comparison of the DFT optimized and X-ray structures of the PERY/ANTH-6-1 charge-transfer complexes. The ANTH-6-1 bend angle θ is defined in Figure S37. The abbreviation A→D(LSP) π–π OOPs denotes the perpendicular displacements from the least-squares plane of the PERY donor $C(sp^2)$ atoms to the nine ANTH-6-1 $C(sp^2)$ atoms that overlap the PERY donor π system. The LSP tilt angle is the dihedral angle between the least-squares planes of the PERY donor and the ANTH-6-1 acceptor $C(sp^2)$ atoms.

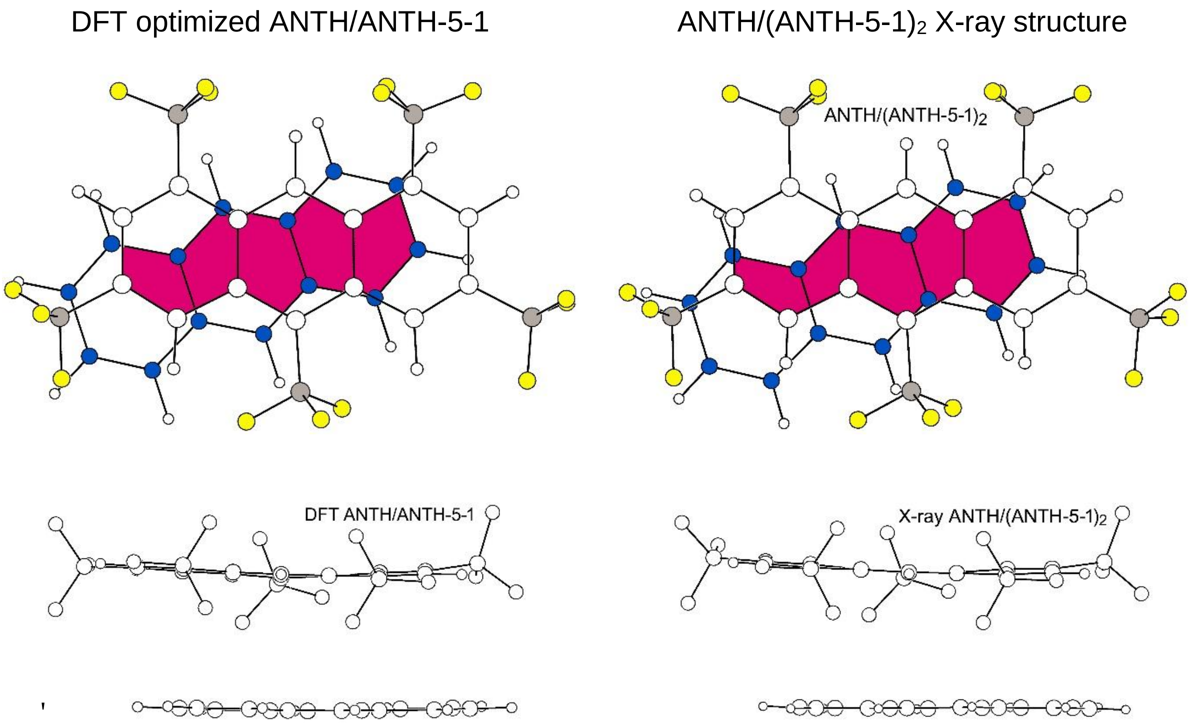

ANTH-6-1 bend angle θ = 5.3°
A→D(LSP) π–π OOPs 3.38–3.50 (ave.3.44 Å)
LSP tilt angle = 1.3°
rotation of D/A major axes = 18.0°

ANTH-6-1 bend angle θ = 5.0°
A→D(LSP) π–π OOPs 3.43–3.54 (ave. 3.49 Å)
LSP tilt angle = 2.2°
rotation of D/A major axes = 17.1°

Figure S42. Comparison of the DFT optimized and X-ray structures of the ANTH/ANTH-5-1 charge-transfer complexes. The ANTH-5-1 bend angle θ is defined in Figure S37. The abbreviation A→D(LSP) π–π OOPs denotes the perpendicular displacements from the least-squares plane of the ANTH donor C(sp^2) atoms to the six (DFT) or seven (X-ray) ANTH-5-1 C(sp^2) atoms that overlap the ANTH donor π system. The LSP tilt angle is the dihedral angle between the least-squares planes of the ANTH donor and the ANTH-6-1 acceptor C(sp^2) atoms.

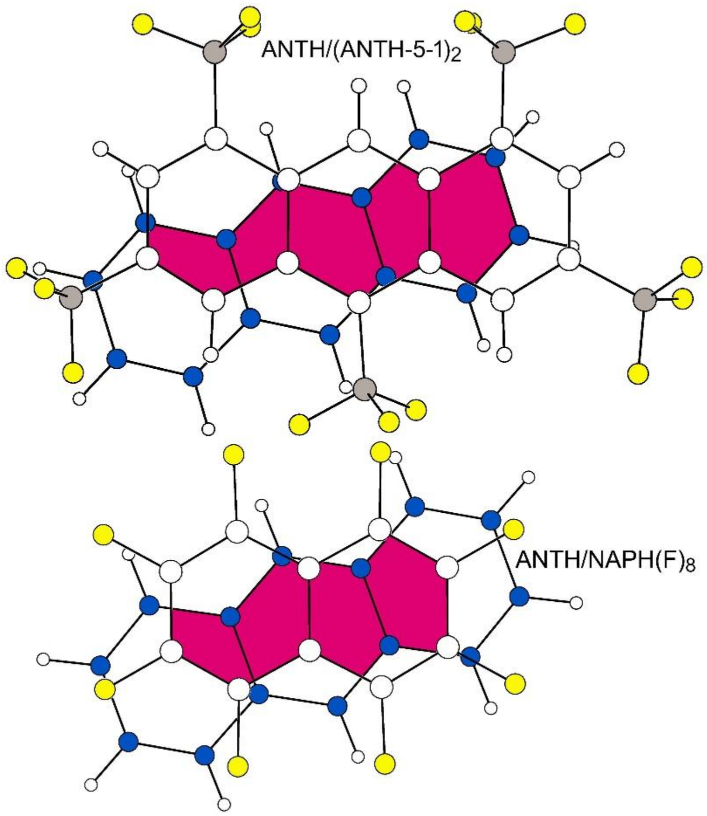

Figure S43. Comparison of the X-ray structures of ANTH/(ANTH-5-1)$_2$ and ANTH/NAPH(F)$_8$ (ref. [65]). Both structures consist of hexagonal arrays of stacks of nearly-parallel donors and acceptors. The rotations of the major axes of ANTH donors and the acceptors with respect to one another are 17.1 and 19.9°, respectively. The D/A LSP dihedral angles are 2.2 and 2.7°, respectively Although the smaller π system of NAPH(F)$_8$ results in a smaller region of π–π overlap in ANTH/NAPH(F)$_8$, the numbers of acceptor C(sp^2) atoms situated within the boundary of the donor π system in the parallel projections shown here only differ by one atom (7 in ANTH/(ANTH-5-1)$_2$ and 6 in ANTH/NAPH(F)$_8$). One important difference between the two structures is that the perpendicular D/A interplanar separations are 3.49 in ANTH/(ANTH-5-1)$_2$ and 3.35 Å in ANTH/NAPH(F)$_8$.

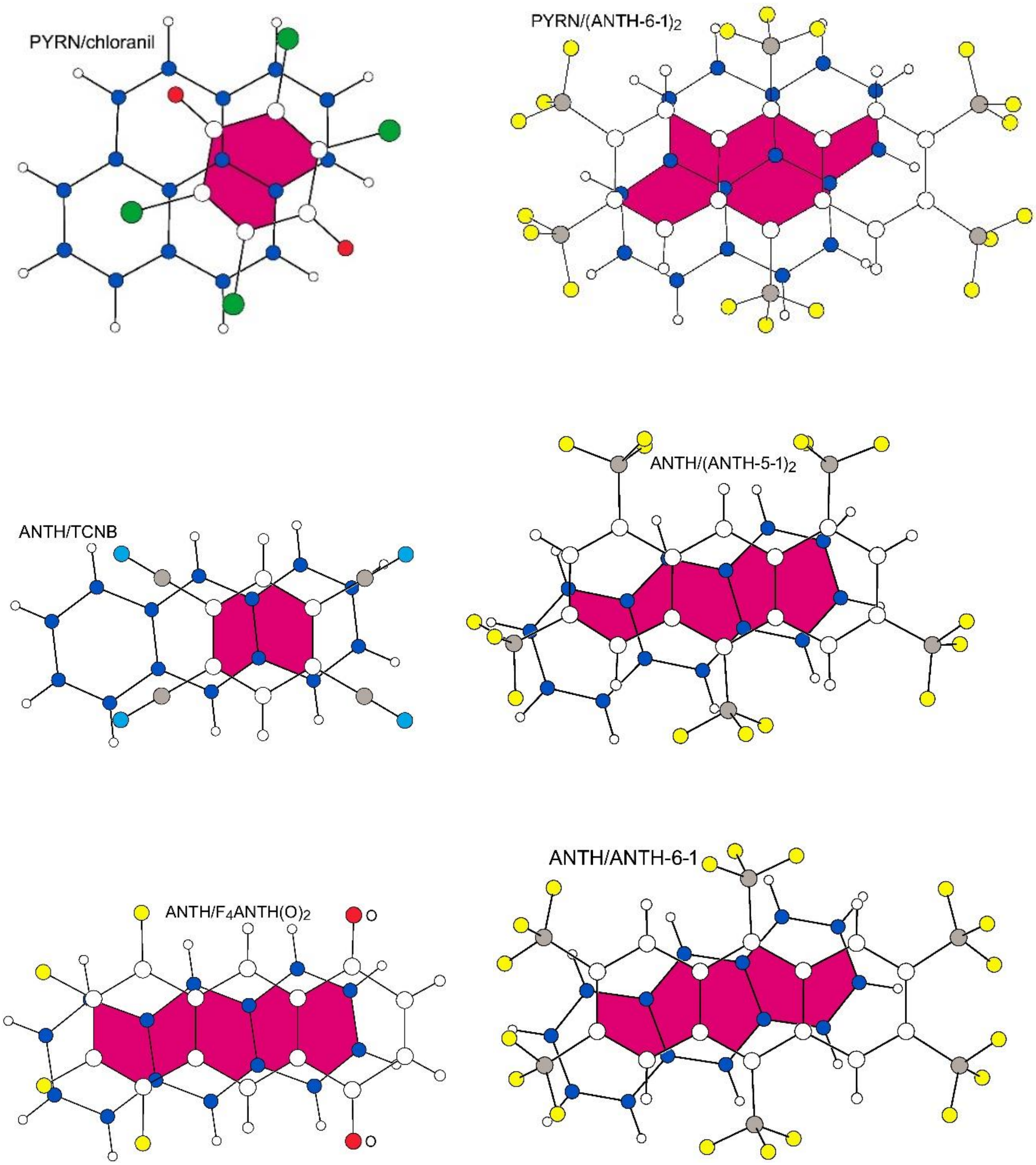

Figure S44. Parallel projection drawings showing the D/A π–π overlap in the X-ray structures of PYRN/chloroanil (top left, ref. [66]), PYRN/(ANTH-6-1)$_2$ (top right, ref. [22]), ANTH/TCNB (middle left, ref. [67]), ANTH/(ANTH-5-1)$_2$ (middle right), ANTH/F$_4$ANTH(O)$_2$ (lower left, ref. [68]; F$_4$ANTH(O)$_2$ = 5,6,7,8,-tetrafluoro-1,4-anthraquinone), and ANTH/ANTH-6-1 (lower right). Chloranil is a common name for tetrachlorobenzoquinone. In each drawing the least-squares plane of the PAH donor C(sp^2) atoms is in the plane of the page. The drawings are to scale.

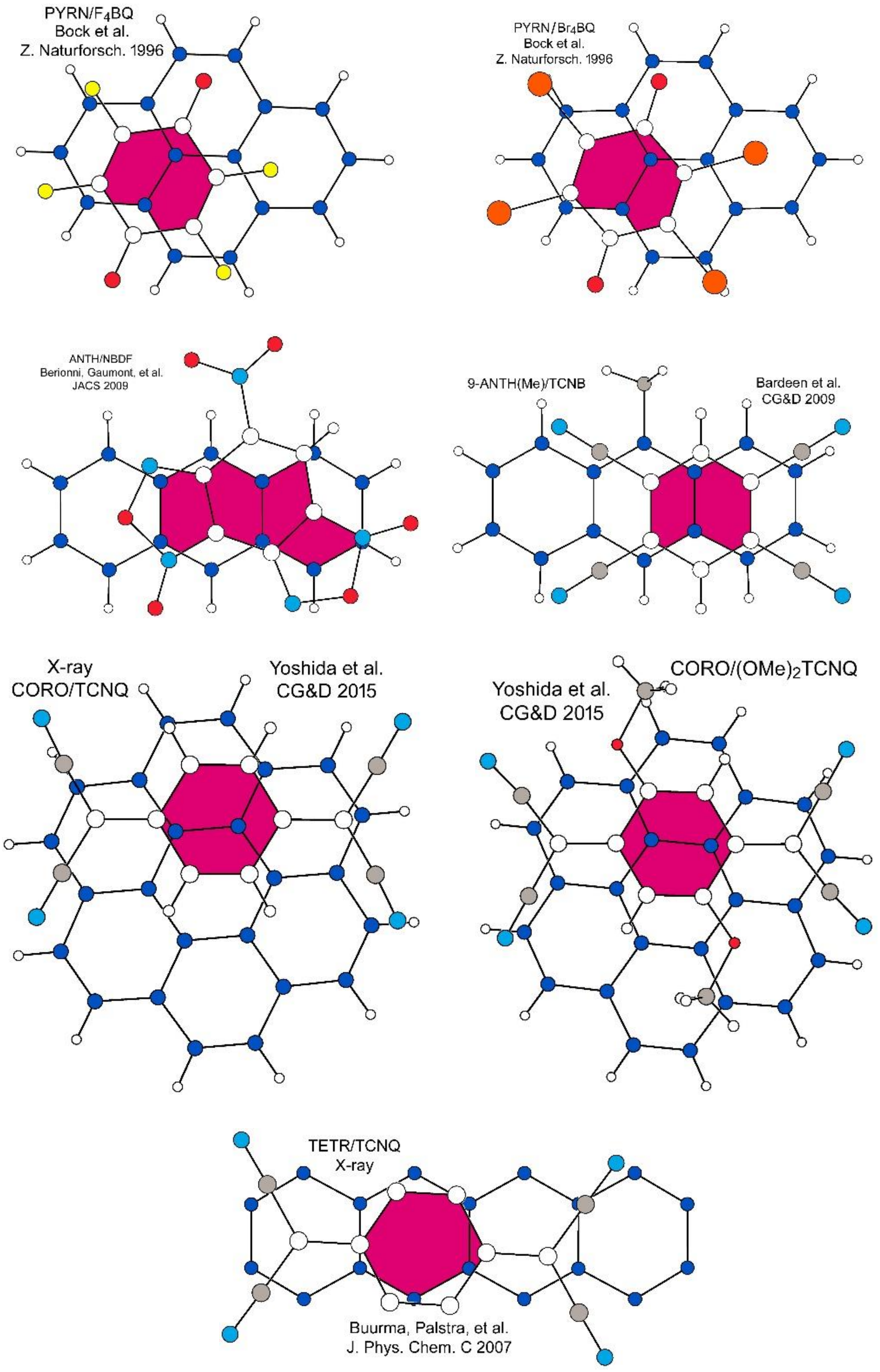

Figure S45. Parallel projection drawings showing the D/A π–π overlap in the X-ray structures of PYRN/F_4BQ (ref. [69]), PYRN/Br_4BQ (ref. [69]), ANTH/NBDF (ref. [23]), 9-ANTH(Me)/TCNB (ref. [70]), CORO/TCNQ (ref. [71]), CORO/$(OMe)_2$TCNQ (ref. [71]), and TETR/TCNQ (ref. [72]; H atoms omitted for clarity). See Table S6 for abbreviations. The drawings are not all to scale.

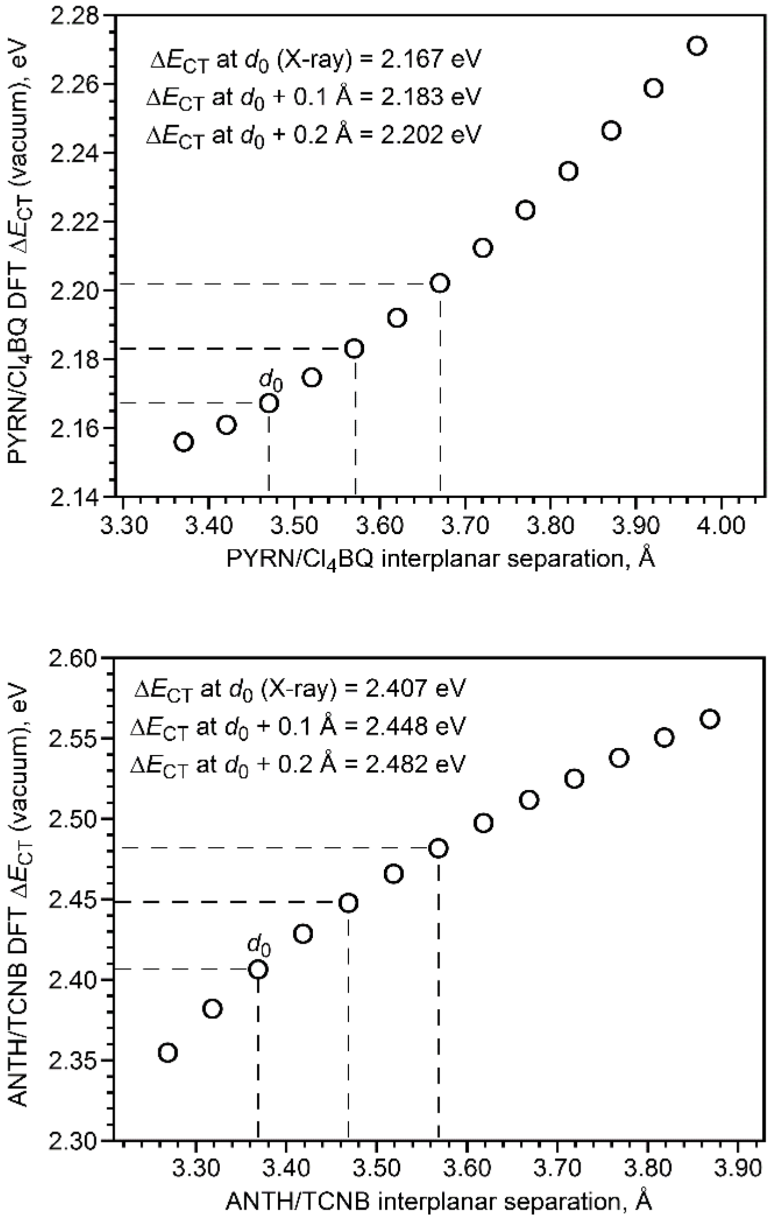

Figure S46. DFT-predicted D/A charge transfer energy (ΔE_{CT}) as a function of donor/acceptor interplanar separation. The distance d_0 in the upper plot is the D/A interplanar distance in the X-ray structure of PYRN/Cl_4BQ (ref. [66]; see also Figure S44). The distance d_0 in the lower plot is the D/A interplanar distance in the X-ray structure of ANTH/TCNB (ref. [67]; see also Figure S44).

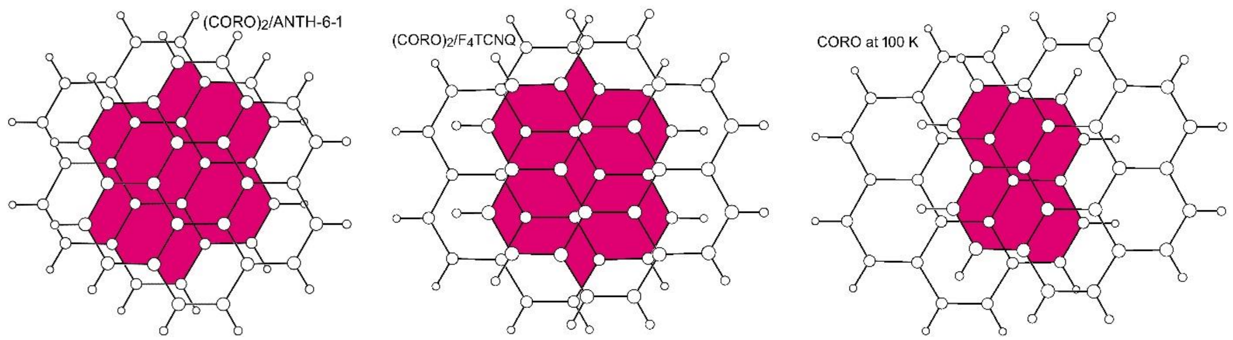

OOPs: Overlapped C(sp^2) atoms with the π system of neighboring molecule (range [#C atoms; average])

3.41–3.49 Å [15; 3.45 Å]	3.42–3.50 Å [12; 3.45 Å]	3.38–3.41 Å [12; 3.40 Å]
major slip: 0.81 Å (11%)	0.15 Å (2.1%)	0.38 Å (5.2%)
minor slip: 1.17 Å (21%)	1.73 Å (30%)	2.91 Å (51%)

Figure S47. CORO/CORO π–π overlap in $(CORO)_2$/ANTH-6-1 (left, this work), in $(CORO)_2/F_4TCNQ$ (middle, refs. [73-74]), and pure CORO at 100 K (right, ref. [74]). The $(CORO)_2$ pair in $(CORO)_2$/ANTH-6-1 exhibits both major (11%) and minor (21%) axis slipage and a greater number of π-overlapped C atoms (15) than the $(CORO)_2$ pair in $(CORO)_2/F_4TCNQ$ (12), which exhibits almost no major axis slippage and 30% minor axis slippage, although the areas of overlap are approximately the same. The neighboring parallel molecules in the structure of pure CORO are on average closer together than in the other two cases (3.40 Å vs. 3.45 Å) but with a much greater minor axis slippage (51%), fewer C atom overlaps, and a much smaller area of π–π overlap.

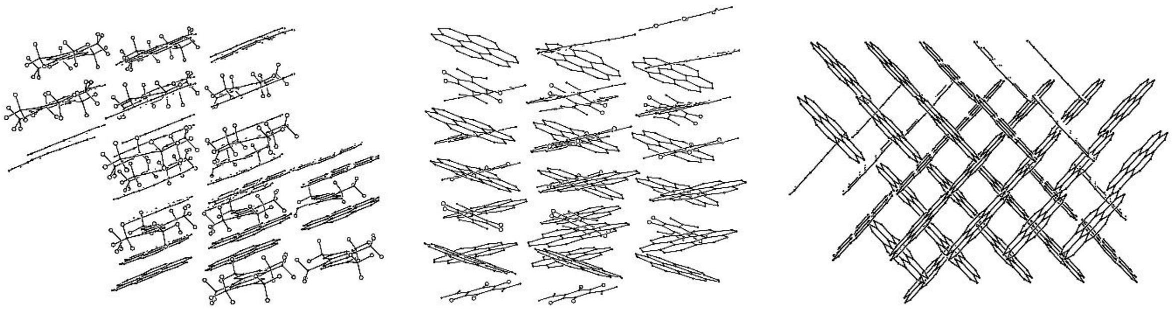

parallel molecules stack-to-stack (ω = 0°) ω = 33.2° ω = 86.3° (herringbone)

Figure S48. Molecular stacking and packing in the structures of $(CORO)_2$/ANTH-6-1 (left, this work), $(CORO)_2/F_4TCNQ$ (middle, refs. [73-74]), and pure CORO (right, ref. [74]).

Supporting Information References

[1] S. Toyota, Y. Watanabe, H. Yoshida, M. Ōki, Synthesis and Structure of 1,4-Dimethoxy and 1,4-Dimethyl-9-(trifluoromethyl)triptycenes, *Bull. Chem. Soc. Japan* **1995**, *68*, 2751–2756.

[2] M. Mintas, H. Gusten, P. G. Williard, Synthesis and [4 + 4] photodimerization of 9-trifluoromethylanthracene, *J. Photochem Photobiol A: Chem.* **1989**, *48*, 341–344.

[3] S. Yamada, K. Kinoshita, S. Iwama, T. Yamazaki, T. Kubota, T. Yajima, Development of novel synthetic routes to bis(perfluoroalkyl)-substituted anthracene derivatives, *RSC Adv.* **2013**, *3*, 6803–6806.

[4] K. P. Castro, "Trifluoromethylated Fullerenes and Polycyclic Aromatic Hydrocarbons and Anaerobically Milled Silicon Nanoparticles," Ph.D. Dissertation thesis, Colorado State University **2015**.

[5] I. V. Kuvychko, K. P. Castro, S. H. M. Deng, X.-B. Wang, S. H. Strauss, O. B. Boltalina, Taming Hot CF_3 Radicals: Incrementally Tuned Families of Polyarene Electron Acceptors for Air-Stable Molecular Optoelectronics, *Angew. Chem. Int. Ed.* **2013**, *52*, 4871–4874.

[6] T. T. Clikeman, E. V. Bukovsky, I. V. Kuvychko, L. K. San, S. H. M. Deng, X.-B. Wang, Y.-S. Chen, S. H. Strauss, O. B. Boltalina, Poly(trifluoromethyl) azulenes: structure and acceptor properties, *Chem. Commun.* **2014**, *50*, 6263–6266.

[7] B. M. Schmidt, S. Seki, B. Topolinski, K. Ohkubo, S. Fukuzumi, H. Sakurai, D. Lentz, Electronic Properties of Trifluoromethylated Corannulenes, *Angew. Chem. Int. Ed.* **2012**, *51*, 11385–11388.

[8] I. V. Kuvychko, T. T. Clikeman, C. Dubceac, Y.-S. Chen, M. A. Petrukhina, S. H. Strauss, A. A. Popov, O. V. Boltalina, Understanding Polyarene Trifluoromethylation with Hot CF_3 Radicals using Corannulene, *Eur. J. Org. Chem.* **2018**, 4233–4245.

[9] B. M. Schmidt, B. Topolinski , M. Yamada , S. Higashibayashi, M. Shionoya, H. Sakurai, D. Lentz, Fluorinated and Trifluoromethylated Corannulenes, *Chem. Eur. J.* **2013**, *19*, 13872–13880.

[10] I. V. Kuvychko, S. N. Spisak, Y. S. Chen, A. A. Popov, M. A. Petrukhina, S. H. Strauss, O. V. Boltalina, A Buckybowl with a Lot of Potential: C_5-$C_{20}H_5(CF_3)_5$, *Angew. Chem. Int. Ed.* **2012**, *51*, 4939–4942.

[11] S. Yamada, S. Iwama, K. Kinoshita, T. Yamazaki, T. Kubota, T. Yajima, Facile synthetic protocols for perfluoroalkyl-substituted diazapentaphenes, *Tetrahedron* **2014**, *70*, 6749–6756.

[12] B. M. Schmidt, A. K. Meyer, D. Lentz, Solid state structures of fluorine-rich fluoranthenes, *CrystEngComm* **2017**, *19*, 1328–1333.

[13] S. Furuta, M. Kuroboshi, T. Hiyama, A Facile Synthesis of Trifluoromethyl- and 3,3,3-Trifluoropropenyl- Substituted Aromatic Compounds by the Oxidative Desulfurization-Fluorination of the Corresponding Carbodithioates, *Bull. Chem. Soc. Japan* **1999**, *72*, 805–819.

[14] K. Hosokawa, K. Inukai, Synthesis of b-Trifluoromethylnaphthalene Derivatives, *Nippon Kagaku Kaishi* **1972**, 383–386.

[15] L. K. San, E. V. Bukovsky, I. V. Kuvychko, A. A. Popov, S. H. Strauss, O. V. Boltalina, Single-Step Gas-Phase Polyperfluoroalkylation of Naphthalene Leads to Thermodynamic Products, *Chem. Eur. J.* **2014**, *20*, 4373–4379.

[16] L. K. San, S. N. Spisak, C. Dubceac, S. H. M. Deng, I. V. Kuvychko, M. A. Petrukhina, X.-B. Wang, A. A. Popov, S. H. Strauss, O. V. Boltalina, Experimental and DFT Studies of the Electron-Withdrawing Ability of Perfluoroalkyl (R_F) Groups: Electron Affinities of PAH(R_F)$_n$ Increase Significantly with Increasing R_F Chain Length, *Chem. Eur. J.* **2018**, *24*, 1441–1447.

[17] C. G. Krespan, B. C. McKusick, T. L. Cairns, Bis-(polyfluoroalkyl)-acetylenes. II. Bicyclooctatrienes Through 1,4-Addition of Bis-(polyfluoroalkyl)-acetylenes to Aromatic Rings, *J. Am. Chem. Soc.* **1961**, *83*, 3428–3432.

[18] Y. Kobayashi, I. Kumadaki, S. Sato, N. Hara, E. Chikami, Studies on Organic Fluorine Compounds. VII. Trifluoromethylation of Aromatic Compounds, *Chem. Pharm. Bull.* **1970**, *18*, 2334–2339.

[19] J. Schwaben, N. Münster, T. Breuer, M. Klues, K. Harms, G. Witte, U. Koert, Synthesis and Solid-State Structures of 6,13-Bis(trifluoromethyl)- and 6,13-Dialkoxypentacene, *Eur. J. Org. Chem.* **2013**, 1639–1643.

[20] S. Yamada, K. Kinoshita, S. Iwama, T. Yamazaki, T. Kubota, T. Yajima, K. Yamamoto, S. Tahara, Synthesis of perfluoroalkylated pentacenes and evaluation of their fundamental physical properties, *Org. Biomol. Chem.* **2017**, *15*, 2522–2535.

[21] K. P. Castro, T. T. Clikeman, N. J. DeWeerd, E. V. Bukovsky, K. C. Rippy, I. V. Kuvychko, G. L. Hou, Y.-S. Chen, X.-B. Wang, S. H. Strauss, O. V. Boltalina, Incremental Tuning Up of Fluorous Phenazine Acceptors, *Chem. Eur. J.* **2016**, *22*, 3930–3936.

[22] N. J. DeWeerd, E. V. Bukovsky, K. P. Castro, I. V. Kuvychko, A. A. Popov, S. H. Strauss, O. V. Boltalina, Steric and electronic effects of CF_3 conformations in acene(CF_3)$_n$ derivatives, *J. Fluorine Chem.* **2019**, *221*, 1–7.

[23] G. Berionni, P. A. Bertelle, J. Marrot, R. Goumont, X-ray Structure of a CT Complex Relevant to Diels-Alder Reactivity of Anthracenes, *J. Am. Chem. Soc.* **2009**, *131*, 18224–18225.

[24] H. Kuroda, M. Kobayashi, M. Kinoshita, S. Takemoto, Semiconductive Properties of Tetracyanoethylene Complexes and Their Absorption Spectra, *J. Chem. Phys.* **1962**, *36*, 457–462.

[25] M. Krishnamurthy, U. Muralikrishna, Solvent Dependent Electronic Absorption Band Shifts of Charge-Transfer Complex Formed between Piperidine and Tetrachloro-1,4-benzoquinone, *Spectroscopy Lett.* **1988**, *21*, 277–283.

[26] R. Beukers, A. Szent-Györgyi, On Charge Transfer Spectra, *Recl. Trav. Chim. Pays-Bas* **1962**, *81*, 255–268.

[27] M. C. R. Cockett, H. Ozeki, K. Okuyhama, K. Kimura, Vibronic coupling in the ground cationic state of naphthalene: A laser threshold photoelectron [zero kinetic energy (ZEKE)-photoelectron] spectroscopic study, *J. Chem. Phys.* **1993**, *98*, 7763–7772.

[28] J. Schiedt, W. J. Knott, K. Le Barbu, E. W. Schlag, R. Weinkauf, Microsolvation of similar-sized aromatic molecules: Photoelectron spectroscopy of bithiophene-, azulene-, and naphthalene-water anion clusters, *J. Chem. Phys.* **2000**, *113*, 9470–9478.

[29] Y. Ling, C. Lifshitz, Time-Dependent Mass Spectra and Breakdown Graphs. 19. Fluoranthene, *J. Phys. Chem.* **1995**, *99*, 11074–11080.

[30] J. Michl, Electronic structure of non-alternant hydrocarbons: Their analogues and derivatives: XVIII. The electronic spectrum and electron affinity of fluoranthene, *J. Mol. Spectros.* **1969**, *30*, 66–76.

[31] J. W. Hager, S. C. Wallace, Two-laser photoionization supersonic jet mass spectrometry of aromatic molecules, *Anal. Chem.* **1988**, *60,* 5–10.

[32] M. Tschurl, U. Boesl, S. Gilb, The Electron Affinity of Phenanthrene, *J. Chem. Phys.* **2006**, *125*, article 194310.

[33] R. S. Becker, E. Chen, Extension of Electron Affinities and Ionization Potentials of Aromatic Hydrocarbons, *J. Chem. Phys.* **1966**, *45*, 2403–2410.

[34] M. Tschurl, U. Boesl, Photodetachment-photoelectron spectroscopy of jet-cooled chrysene, *Int. J. Mass Spectrom.* **2006**, *249*, 364–369.

[35] N. Ando, M. Mitsui, A. Nakajima, Comprehensive photoelectron spectroscopic study of anionic clusters of anthracene and its alkyl derivatives: Electronic structures bridging molecules to bulk, *J. Chem. Phys.* **2007**, *127,* article 234305.

[36] G. D. Chen, R. G. Cooks, Electron affinities of polycyclic aromatic hydrocarbons determined by the kinetic method, *J. Mass Spectrom.* **1995**, *30*, 1167–1173.

[37] N. Ando, S. Kokubo, M. Mitsui, A. Nakajima, Photoelectron spectroscopy of pyrene cluster anions, $(\text{pyrene})_n^-$ (n=1–20), *Chem. Phys. Lett.* **2004**, *389*, 279–283.

[38] M. Meot-Ner, Ion thermochemistry of low volatility compounds in the gas phase. 3. Polycyclic aromatics: Ionization energies, proton, and hydrogen affinities. Extrapolations to graphite, *J. Phys. Chem.* **1980**, *84*, 2716–2723.

[39] M. A. Duncan, A. M. Knight, Y. Negishi, S. Nagao, Y. Nakamura, A. Kato, A. Nakajima, K. Kaya, Production of jet-cooled coronene and coronene cluster anions and their study with photoelectron spectroscopy, *Chem. Phys. Lett.* **1999**, *309*, 49–54.

[40] M. I. Shchuka, A. L. Motyka, M. R. Topp, Two-photon threshold ionization spectroscopy of perylene and van der waals complexes, *Chem. Phys. Lett.* **1989**, *164*, 87–95.

[41] J. Schiedt, R. Weinkauf, Photodetachment photoelectron spectroscopy of mass selected anions: anthracene and the anthracene–H_2O cluster, *Chem. Phys. Lett.* **1997**, *266*, 201–205.

[42] M. Mitsui, N. Ando, A. Nakajima, Mass Spectrometry and Photoelectron Spectroscopy of Tetracene Cluster Anions, (Tetracene)$_n^-$ (n = 1−100): Evidence for the Highly Localized Nature of Polarization in a Cluster Analogue of Oligoacene Crystals, *J. Phys. Chem. A* **2007**, *111*, 9644–9648.

[43] L. Crocker, T. B. Wang, P. Kebarle, Electron Affinities of Some Polycyclic Aromatic Hydrocarbons, Obtained from Electron-Transfer Equilibria, *J. Am. Chem. Soc.* **1993**, *115*, 7818–7822.

[44] NIST Webbook.

[45] E. S. Pysh, N. C. Yang, Polarographic Oxidation Potentials of Aromatic Compounds, *J. Am. Chem. Soc.* **1963**, *85*, 2124–2130.

[46] J. R. Aranzaes, M.-C. Daneil, D. Astruc, Metallocenes as references for the determination of redox potentials by cyclic voltammetry. Permethylated iron and cobalt sandwich complexes, inhibition by polyamine dendrimers, and the role of hydroxy-containing ferrocenes, *Can. J. Chem.* **2006**, *84*, 288–299.

[47] J. M. Masnovi, E. A. Seddon, J. K. Kochi, Electron transfer from anthracenes. Comparison of photoionization, charge-transfer excitation and electrochemical oxidation, *Can. J. Chem.* **1984**, *62*, 2552–2559.

[48] T. Heinis, S. L. Chowdhury, S. Scott, P. Kebarle, Electron Affinities of Benzo-, Naphtho-, and Anthraquinones Determined from Gas-Phase Equilibria Measurements, *J. Am. Chem. Soc.* **1988**, *110*, 400–407.

[49] G. Paul, P. Kebarle, Electron Affinities of Cyclic Unsaturated Dicarbonyls: Maleic Anhydrides, Maleimides, and Cyclopentenedione, *J. Am. Chem. Soc.* **1989**, *111*, 464–470.

[50] A. L. Farragher, F. M. Page, Experimental Determination of Electron Affinities. Part II.—Electron Capture by Some Cyanocarbons and Related Compounds, *Trans. Faraday Soc.* **1967**, *63*, 2369–2378.

[51] G.-Z. Zhu, L.-S. Wang, Communication: Vibrationally resolved photoelectron spectroscopy of the tetracyanoquinodimethane (TCNQ) anion and accurate determination of the electron affinity of TCNQ, *J. Chem. Phys.* **2015**, *143*, article 221102.

[52] D. Khuseynov, M. T. Fontana, A. Sanov, Photoelectron spectroscopy and photochemistry of tetracyanoethylene radical anion in the gas phase, *Chem. Phys. Lett.* **2012**, *550*, 15–18.

[53] D. A. Horke, G. M. Roberts, J. R. R. Verlet, Excited States in Electron-Transfer Reaction Products: Ultrafast Relaxation Dynamics of an Isolated Acceptor Radical Anion *J. Phys. Chem,. A* **2011**, *115*, 8369–8374.

[54] J. Calbo, R. Viruela, E. Ortí, J. Aragó, Relationship between Electron Affinity and Half-Wave Reduction Potential: A Theoretical Study on Cyclic Electron-Acceptor Compounds, *ChemPhysChem* **2016**, *17*, 3881–3890.

[55] M. Lotfi, R. M. G. Roberts, Correlation of thermodynamic stabilities of charge transfer complexes of anthracene and chloranil with those of tetra-cyanoethylene, *Tetrahedron* **1979**, *35*, 2123–2129.

[56] B. K. Seal, H. Sil, D. C. Mukherjee, Independent Determination of Equilibrium-Constant and Molar Extinction Coefficient of Molecular-Complexes From Spectrophotometric Data By a Graphical-Method, *Spectrochem. Acta Part A* **1982**, *38*, 289–292.

[57] M. Chowdhury, S. Basu, Charge transfer interaction between tetrachlorophthalic anhydride and aromatics, *Trans. Faraday Soc.* **1960**, *56*, 355–339.

[58] C. J. Eckhardt, R. R. Pennelly, Selection Rules for the Charge Transfer Transitions in the Symmetrical Tetracyano-*p*-quinodimethane-Anthracene Molecular Complex, *J. Am. Chem. Soc.* **1976**, *98*, 2034–2040.

[59] R. Foster, T. J. Thomson, Interaction of Electron Acceptors with Bases. Part 10 – Complexes of Polycyanobenzenes with Electron Donors, *Trans. Faraday Soc.* **1963**, *59*, 2287–2295.

[60] G. Briegleb, J. Czekalla, Electron Transition by Means of Light Absorption and Emission in Electron Donor-Acceptor Complexes, *Angew. Chem.* **1960**, *72*, 401–413.

[61] A. Zweig, J. E. Lehnsen, W. G. Hodgson, W. H. Jura, π-System Properties of Pyromellitonitrile, *J. Am. Chem. Soc.* **1963**, *85*, 3937–3939.

[62] X. Zhang, Q. Li, J. B. Ingels, A. C. Simmonett, S. E. Wheeler, Y. Xie, R. B. King, H. F. Schaefer III, F. A. Cotton, Remarkable electron accepting properties of the simplest benzenoid cyanocarbons: hexacyanobenzene, octacyanonaphthalene and decacyanoanthracene, *Chem. Commun.* **2006**, 758–760.

[63] Y. Yoshida, Y. Shimizu, T. Yajima, G. Maruta, S. Takeda, Y. Nakano, T. Hiramatsu, H. Kageyama, H. Yamochi, G. Saito, Molecular Rotors of Coronene in Charge-Transfer Solids, *Chem. Eur. J.* **2013**, *19*, 12313–12324.

[64] T. Akutagawa, M. Uchigata, T. Hasegawa, T. Nakamura, K. A. Nielsen, J. O. Jeppesen, T. Brimert, J. Becher, Langmuir–Blodgett Films of Charge-Transfer Complexes between an Amphiphilic Monopyrrolo-TTF and TCNQ Derivatives, *J. Phys. Chem. B* **2003**, *107*, 13929–13938.

[65] J. C. Collings, K. P. Roscoe, R. L. Thomas, A. S. Batsanov, L. M. Stimson, J. A. K. Howard, T. B. Marder, Arene-perfluoroarene interactions in crystal engineering. Part 3. Single-crystal structures of 1 : 1 complexes of octafluoronaphthalene with fused-ring polyaromatic hydrocarbons, *New J. Chem.* **2001**, *25*, 1410–1417.

[66] K. Prout, I. J. Tickle, Molecular Complexes. Part XXII. Crystal and Molecular Structure of the Molecular Complex of Pyrene and Chloranil, *J. Chem. Soc. Perkin Trans. 2* **1973**, 1212–1215.

[67] J. Lefebvre, G. Odou, M. Muller, A. Mierzejewski, T. Luty, Characterization of an Orientational Disorder in Two Charge-Transfer Complexes: Anthracene-Tetracyanobenzene (A-TCNB) and Naphthalene--Tetracyanobenzene (N-TCNB), *Acta Crystallogr., Sect. B: Struct. Sci.* **1989**, *45*, 323–336.

[68] H. Chen, F. Gao, E. D. Yao, Q. Chen, Y. G. Ma, Rapid mechanochemical preparation of a sandwich-like charge transfer complex, *CrystEngComm* **2013**, *15*, 4413-4416.

[69] H. Bock, M. Sievert, H. Schödel, M. Kleine, Interactions in crystals .88. Donor/acceptor complexes of alkylbenzenes, pyrene or perylene and tetrahalogen-*p*-benzoquinones: Structures and properties, *Z. Naturforsch. B* **1996**, *51*, 1521–1537.

[70] R. O. Al-Kaysi, A. M. Müller, R. J. Frisbee, C. J. Bardeen, Formation of Cocrystal Nanorods by Solid-State Reaction of Tetracyanobenzene in 9-Methylanthracene Molecular Crystal Nanorods, *Cryst. Growth Des.* **2009**, *9*, 1780–1785.

[71] Y. Yoshida, Y. Kumagai, M. Mizuno, G. Saito, Structure−Property Relationship of Supramolecular Rotators of Coronene in Charge-Transfer Solids, *Cryst. Growth Des.* **2015**, *15*, 1389−1394.

[72] A. J. C. Buurma, O. D. Jurchescu, I. Shokaryev, J. Baas, A. Meetsma, G. A. de Wijs, R. A. de Groot, T. T. M. Palstra, Crystal Growth, Structure, and Electronic Band Structure of Tetracene−TCNQ, *J. Phys. Chem. C* **2007**, *111*, 3486–3489.

[73] Y. Yoshida, Y. Kumagai, M. Mizuno, K. Isomura, Y. Nakamura, H. Kishida, G. Saito, Improved Dynamic Properties of Charge-Transfer-Type Supramolecular Rotor Composed of Coronene and F_4TCNQ, *Cryst. Growth Des.* **2015**, *15*, 5513−5518.

[74] O. Kataeva, M. Khrizanforov, Y. Budnikova, D. Islamov, T. Burganov, A. Vandyukov, K. Lyssenko, B. Mahns, M. Nohr, S. Hampel, M. Knupfer, Crystal Growth, Dynamic and Charge Transfer Properties of New Coronene Charge Transfer Complexes, *Cryst. Growth Des.* **2016**, *16*, 331−338.
